# Natural Products as Alternative Choices for P-Glycoprotein (P-gp) Inhibition

**DOI:** 10.3390/molecules22060871

**Published:** 2017-05-25

**Authors:** Saikat Dewanjee, Tarun K. Dua, Niloy Bhattacharjee, Anup Das, Moumita Gangopadhyay, Ritu Khanra, Swarnalata Joardar, Muhammad Riaz, Vincenzo De Feo, Muhammad Zia-Ul-Haq

**Affiliations:** 1 Advanced Pharmacognosy Research Laboratory, Department of Pharmaceutical Technology, Jadavpur University, Raja S C Mullick Road, Kolkata 700032, India; tarunkduaju@gmail.com (T.K.D.); bniloy5@gmail.com (N.B.); ritukhanra@yahoo.co.in (R.K.); swarnalatajoardar@yahoo.in (S.J.); 2 Department of Pharmaceutical Technology, ADAMAS University, Barasat, Kolkata 700126, India; anup.das82@gmail.com; 3 Department of Bioechnology, ADAMAS University, Barasat, Kolkata 700126, India; gangopadhyaymoumita75@gmail.com; 4 Department of Pharmacy, Shaheed Benazir Bhutto University, Sheringal 18050, Pakistan; pharmariaz@gmail.com; 5 Department of Pharmacy, Salerno University, Fisciano, 84084 Salerno, Italy; 6 Environment Science Department, Lahore College for Women University, Jail Road, Lahore 54600, Pakistan

**Keywords:** P-glycoprotein (P-gp), multi drug resistance (MDR), ABC transporters, P-gp inhibitors, chemotherapy, xenobiotics

## Abstract

Multidrug resistance (MDR) is regarded as one of the bottlenecks of successful clinical treatment for numerous chemotherapeutic agents. Multiple key regulators are alleged to be responsible for MDR and making the treatment regimens ineffective. In this review, we discuss MDR in relation to P-glycoprotein (P-gp) and its down-regulation by natural bioactive molecules. P-gp, a unique ATP-dependent membrane transport protein, is one of those key regulators which are present in the lining of the colon, endothelial cells of the blood brain barrier (BBB), bile duct, adrenal gland, kidney tubules, small intestine, pancreatic ducts and in many other tissues like heart, lungs, spleen, skeletal muscles, etc. Due to its diverse tissue distribution, P-gp is a novel protective barrier to stop the intake of xenobiotics into the human body. Over-expression of P-gp leads to decreased intracellular accretion of many chemotherapeutic agents thus assisting in the development of MDR. Eventually, the effectiveness of these drugs is decreased. P-gp inhibitors act by altering intracellular ATP levels which are the source of energy and/or by affecting membrane contours to increase permeability. However, the use of synthetic inhibitors is known to cause serious toxicities. For this reason, the search for more potent and less toxic P-gp inhibitors of natural origin is underway. The present review aims to recapitulate the research findings on bioactive constituents of natural origin with P-gp inhibition characteristics. Natural bioactive constituents with P-gp modulating effects offer great potential for semi-synthetic modification to produce new scaffolds which could serve as valuable investigative tools to recognize the function of complex ABC transporters apart from evading the systemic toxicities shown by synthetic counterparts. Despite the many published scientific findings encompassing P-gp inhibitors, however, this article stand alones because it provides a vivid picture to the readers pertaining to Pgp inhibitors obtained from natural sources coupled with their mode of action and structures. It provides first-hand information to the scientists working in the field of drug discovery to further synthesise and discover new P-gp inhibitors with less toxicity and more efficacies.

## 1. Introduction

Living organisms through constant evolution have developed defense mechanisms against persistent attacks from environmental toxins as it is necessary for them to avoid the pernicious effects of these cytotoxic compounds. Among the several defense mechanisms involved the one that pumps out toxic substances (through efflux pumps) from the intracellular space of the cells is documented both in bacterial and mammalian cells. Overexpression of these pumps in cancer cells is a key regulator of drug resistance during cancer chemotherapy. In mammalian cells, this MDR phenotype was first discovered ~40 years ago, by Ling and co-workers [1].

Ling’s group noted that ovary cells of Chinese hamster that showed resistance to colchicine also displayed resistance towards a broad spectrum of cytotoxic agents, however, very surprisingly the shared little or minimal similarity in their chemical structures or modes of cytotoxicity with colchicine. This phenomenon later became known as MDR. Through molecular cloning and functional characterization studies, this resistant phenotype was linked with a 170 kDa surface glycoprotein, which they named as permeability glycoprotein (P-gp; subfamily B, member 1: ABCB1; also known as MDR1) for its capability to impede the cellular permeability of cytotoxic drugs [2].

**Figure 1:**
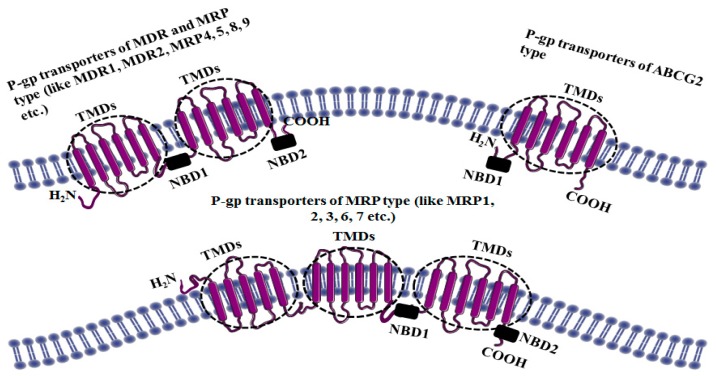
Basic structures of different types of P-gp transporters [2].

Later on, it was understood that MDR1/P-gp alone cannot be responsible for every type of MDR and this eventually led to the discovery of other related transporters, especially breast cancer–resistance protein (BCRP, also known as ABCG2) and MDR-associated protein (MRP1 also known as ABCC1). Amino acid sequence analysis indicated that all these MRPs comprise multiple transmembrane domains (TMDs) and intracellularly confined ATP binding cassettes (ABCs) or nucleotide binding domains (NBDs) (Figure 1).

These transmembrane domains act as the channels whereby animal cells utilize the intracellularly placed ABCs to hydrolyze ATP to provide energy to expel the cytotoxic drugs out through TMDs thus reducing intracellular drug concentrations to a sub-lethal level. The availability of different types of ABC transporter proteins and the broad substrate specificity shown by these might explain the complexity faced during the past couple of decades in attempting to thwart ABC-mediated MDR in vivo. Although scientists have worked hard to develop drugs that either impede the function of efflux transporters or evade efflux, progress in this area has been slow. In spite of this, the urge to understand the underlying principles involved in MDR is still strong and thorough understanding of these transporters is an absolute must to find an alternative to synthetic drugs which are frequently associated with systemic toxicity.

Several protozoal parasites (*Plasmodium*, *Leishmania*, *Trypanosoma*) and some bacteria were observed to produce resistance against chemotherapeutic agents, such as quinolines, naphthoquinones, sesquiterpene lactones, and other anti-microbial agents. The underlying mechanism includes membrane glycoproteins that are orthologous to human P-gp. These ABC transporters can efflux their substrates via different mechanisms. These transporters can be modulated and activated via several natural and synthetic molecules with diverse mechanisms.

Many bioactive molecules of natural origin are already known for their promising therapeutic effects in various ailments. The use of natural products in P-gp inhibition is not new to the scientific community and it has been used in practice for more than three decades. Notably among those are fruits like grapes, citrus fruits [3], glycosides (picroside, etoposide, etc.) alkaloids (vinblastine, aconitum, vincristine, campothecin, irinotecan, etc.) [4], flavonoids and phenolics (quercetin, naringin, epigallocatechin, etc.) [5], terpenoids (citronellal, citral, safranal, etc.) [6], taxols like paclitaxel, anthracyclines (doxorubicine, daunorubicine, etc.) and epipodophyllotoxins (etoposide, teneposide, etc.) [5].

Natural products are known for their low toxicity and higher specificity towards P-gp [7]. Due to their low toxicity profile and high specificity, research on finding P-gp inhibitors is becoming a point of interest for modern researchers. Henceforth, the prime objective of this review is to provide a detailed overview of the various novel P-gp inhibitors from natural sources and information regarding their structures as well as mechanism of action. Additionally, in the subsequent sections an attempt is made to briefly summarize and understand P-gp tissue distribution and structure, its mechanism of action, pathophysiological and pharmacokinetic roles in MDR.

Although many scientific findings in the form of research papers and review articles have been published encompassing P-gp inhibitors, this review stands alone with respect to others in many aspects. A vast array of research papers were delved through to provide a vivid picture to the readers pertaining to Pgp inhibitors obtained from natural sources. The mechanism of action of most of the the reported phytocompounds are represented meticulously throughout the text along with the structures. This will give first-hand information to the medicinal chemists and scientists working in the field of drug discovery to further synthesise and discover new scaffolds with highest efficacy in the future.

## 2. Distribution and Functions of P-gp

Two members of the P-gp gene family, namely MDR1 and MDR3, exist in humans, whereas three members of this family, viz. MDR1A, MDR1B and MDR2, are found in animals [8]. The human MDR1 is widely distributed and is known to excrete a wide range of drugs across the cell membrane, whereas MDR3 shows limited expression. However, MDR3 shows its highest expression in the canalicular membranes of hepatocytes [9]. The contribution of human MDR3 in drug transport has been recently observed. Low rate of MDR3-mediated transport for most drugs explains why this protein has no role in MDR or hold any significant pharmacological importance [10].

The human MDR1 is ubiquitously expressed and is perhaps one of the most significant ABC transporters for drug disposal in humans and thus carries pharmacological importance. It has been identified as a primary cause of MDR. Functionally, the P-gp efflux transporter protects our body against orally ingested or airborne toxins, xenobiotics or drugs by excreting them into bile, urine and intestinal lumen thus inhibiting their impact in brain, testis and placenta. It is substantially involved in many drug interactions and thus carries some pharmacokinetic importance as well. Thus, in the subsequent sections we have summarized MDR1/P-gp tissue distribution, structure, its mechanism of action, pathophysiological and pharmacokinetic roles in MDR.

### 2.1. P-gp Distribution in Cancer Cells

Many studies have been carried out in recent years to investigate the expression of P-gp in solid tumors and haematologic cancers besides determination of its clinical importance [11]. Several techniques have been used so far for determining expression of the MDRI gene. Several of these, for example RNAase protection assay, northern blot, dot blot, in situ hybridization and RT-PCR were used to study the mRNA levels of the MDR1 gene. Other assays, including western blot, immune-histochemistry and flow cytometry, analyzed P-gp at their protein level using various monoclonal antibodies directed against extracellular or intracellular epitopes of the pump [12].

Additionally, in some studies flow cytometry technique coupled with well-known fluorescent substrates such as anthracyclines and rhodamine 123 (Rh 123) have been used for estimation of P-gp activity [13]. High P-gp expression is typically seen in tumors arising from tissues known to physiologically express the pump, such as carcinoma of the pancreas, colon, liver, adrenal gland, and kidney [14]. Intermediate P-gp expression has been observed during diagnosis stage in neuroblastomas [15], myelodysplatic syndromes [16], acute and chronic myeloid leukemias [17]. Usually, a low level of P-gp expression has been observed in tumors of the lung, ovary, breast, esophagus, and stomach [18,19]. However, some of these cases especially acute myeloid leukemias, breast tumors, lymphomas, and myelomas may present elevated levels of P-gp expression soon after chemotherapy which, thereby, leads to the development of acquired drug resistance [20,21].

### 2.2. P-gp Distribution in Normal Human Tissues

**Figure 2:**
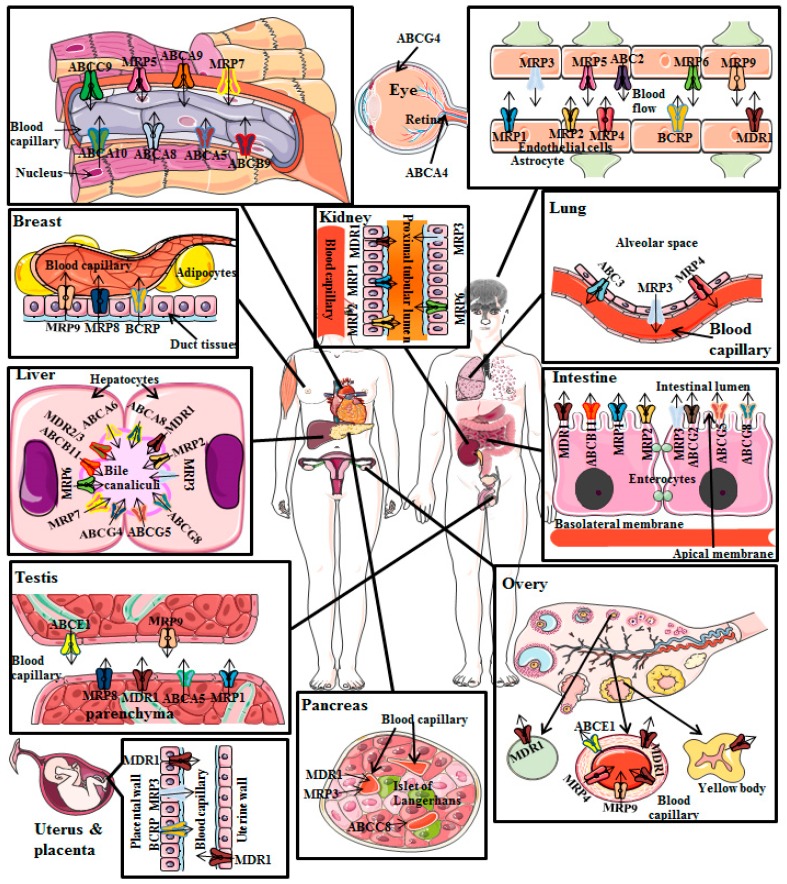
Overview of P-gp functional expressions throughout the body. Black lines indicate the location of ABCB1. Small arrows indicate the direction of ABCB1-mediated transport [9,10,22,23,24,25,26].

Besides its location in tumor cells and its role in resistance to chemotherapy, P-gp is also expressed in normal human tissues, as shown in Figure 2. Monoclonal antibody coupled immune-histochemical analyses revealed that P-gp is primarily and physiologically expressed at the apical or luminal membrane of normal tissues of several secretory organs like liver, adrenal gland, kidney and at the juncture of barrier tissues like BBB, blood-testis barrier, ovarian blood barrier and placental barrier [22].

In the liver, P-gp is found entirely on the bile canalicular of hepatocytes and on the apical surface of epithelial cells of small bile ducts. Its major function is the elimination of drugs and toxins into the bile. In pancreas, P-gp is located only on the upper surface of the epithelial cells of small ducts but not on the larger pancreatic ducts. In kidney, P-gp is mainly located on the upper surface of epithelial cells of the proximal convoluted tubules. High level of P-gp is generally found on the apical surfaces of superficial columnar epithelial cells of colon and jejunum. P-gp is also known to be diffusely distributed on the cell surface of cortex and medulla of adrenal gland [23]. Some mononuclear peripheral blood cells, such as cytotoxic T cells and natural killer cells also express P-gp, thus, indicating that P-gp may have a role in cell-mediated cytotoxicity [24]. In addition, P-gp has been shown to be expressed and working in human hematopoietic stem cells suggesting that P-gp may lead to chemo resistance as well [25]. Following this tissue localization, P-gp acts in three main areas:

(i) P-gp restricts drugs’ entry after oral administration as a result of its presence in the apical membrane of enterocytes of intestine;
(ii) Once the drugs and/or xenobiotics have entered into the systemic circulation, P-gp induces elimination of drugs through urine and bile because of its presence in the canalicular membrane of hepatocytes and in the apical surface of kidney’s proximal convoluted tubular cells, respectively;
(iii) Additionally, P-gp decreases entry of drugs into sensitive tissues particularly in the BBB [26].

This localization strongly indicates a significant function of P-gp as an efflux pump, which limits the infiltration of drugs and/or xenobiotics into the central nervous system, thus acting as a major gatekeeper.

### 2.3. Distribution of Multidrug Efflux Systems in Microorganisms

**Figure 3:**
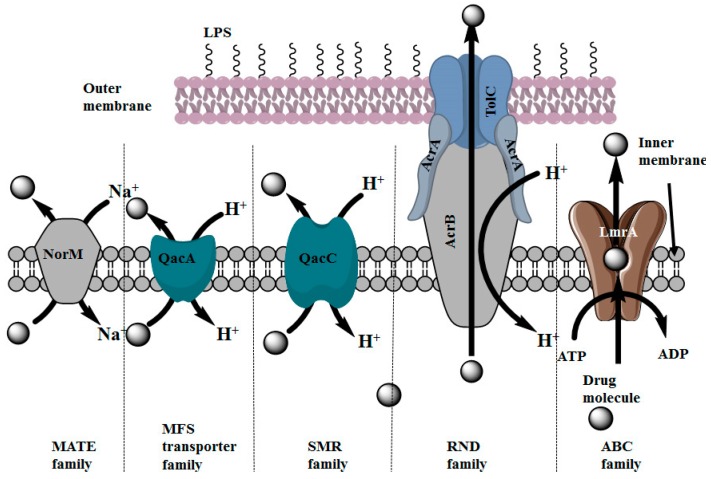
Schematic diagram representing different types of drug transporters present in microorganisms [27,28,29,30,31,32,33].

Multidrug transporters are also present in microorganisms like bacteria and fungi. Bacterial efflux pumps primarily classified into two major superfamilies which are: primary transporters and secondary transporters. ABC transporters are the primary transporters of the bacterial efflux system. These ABC transporters are widely present and ubiquitous to both prokaryote and eukaryote membrane systems [27]. In bacteria, the ABC transporters acquired high specificity for substrates, like antibiotics, vitamins, amino acids and sugars [28,29]. The ABC transporters are reported in Gram positive bacteria, where these transporters confer resistance to macrolides and bacitracin [30]. Bacterial efflux systems which are classified as secondary transporters include the following super-families: major facilitator superfamily (MFS), resistance nodulation division (RND) superfamily, small multidrug resistance (SMR) superfamily, multidrug and toxic compound extrusion (MATE) superfamily [31,32] (Figure 3). Of these efflux pump families, the RND and MFS efflux pumps are ubiquitous systems [33]. Examples of some natural molecules which inhibit microbial efflux pumps are mentioned later in this review. Many of these natural molecules shows promising drug efflux inhibitory activity on cancer cell as well as on microbes. These examples have established a relation between inhibitors of microbial and human ABC transporters. Futher research is needed to understand the possibilities of other inhibitors from natural sources in the inhibition of both the human as well as microbial ABC transporters.

## 3. Structure of P-gp

**Figure 4:**
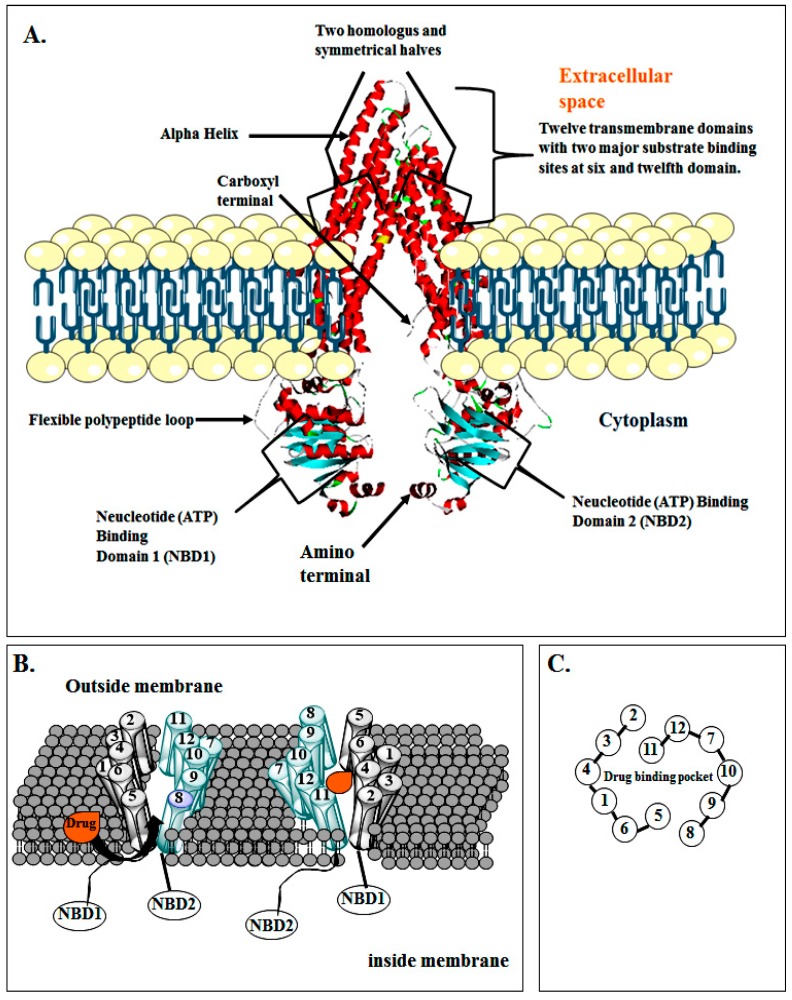
Schematic diagrams representing: (**A**) Structure of drug transporting P-gp; (**B**) drug binding pocket of P-gp; (**C**) drug binding pocket of P-gp surrounded by TMs [2,35,36,37,38,39,40,41,42].

P-gp, a 170-kDa ABC transporter, comprised of 1280 amino acids, is an energy-dependent drug efflux pump encoded by the human MDR1 gene [34]. Sequence analysis of amino acids obtained from cloned cDNAs and comparison with other ABC family members suggest that human P-gp comprises of two symmetrical amino (N)-and carboxyl (C)-terminal halves (cassettes) which are 43% identical [35] and each of which comprises of six TM domains that related to each other by an intracellular flexible linker polypeptide loop, about 80 amino acids in length with an ATP-binding motif as shown in Figure 4 [36].

Intracellularly, there are two ATP-binding domains, which are also known as nucleotide-binding domains (NBDs) which constitute the power units of P-gp. The NBDs are located in the cytoplasm and transfers energy to transport the substrates across the membranes. Each ATP-binding domain comprises three segments, namely Walker A, B, and signature C motifs. Walker A motif which contains a highly-conserved Lys residue of histidine permease has a direct role in binding of ATP [37] and a well conserved Asp residue within the walker B motif assists in binding of Mg^2+^ ion. P-gp requires both Mg^2+^-ATP-binding and ATP hydrolysis to function as a drug transporter. It has also been postulated that magnesium can work for stabilizing the ATP-binding site [38].

Signature C motifs is probably involved in accelerating the hydrolysis of ATP via some chemical transition [39] and is also thought to be involved in the transduction of the energy of ATP hydrolysis to the conformational changes in the TM domains needed for translocation of the substrate [40]. Unlike the ATP binding sites which are limited to Walker A units of ATP binding domains, many substrate binding sites were recognized throughout the transmembrane (TM) domain of P-gp. The key drug-binding sites reside in or near TM6 and TM12 [41]. Moreover, TM1, TM4, TM10, and TM11 are also involved in drug binding [42]. The amino acids in TM1 are included in the formation of a binding pocket that has a role in defining the appropriate size of the substrate of P-gp, whereas Gly residues in TMs 2 and 3 play an important role in assessment of the substrate specificity. The close proximity of TM2 with TM11 and TM5 with TM8 as shown in Figure 4 indicates that this area between the two segments must include the drug binding pocket at the cytoplasmic side of P-gp.

P-gp in its resting state shows “closed” conformation of NBD1 and NBD2. The intracellular/cytoplasmic ends of the TMDs (i.e., NBD1 and NBD2) are near to each other but remains open at the extracellular end of the molecule. A perplexing issue is how substrates pass this drug binding pocket when P-gp is in its active state. One observation is that two “drug binding pockets” are formed in the lipid bilayer, one between TM2 of TMD1 and TM11 of TMD2 at one side of the drug binding pocket, and the other between TM5 of TMD1 and TM8 of TMD2 at the opposite side, as mentioned earlier and shown in Figure 4.

If the drug binding pocket is hydrophilic and the substrates are hydrophobic by nature, entry of substrates into this type drug-binding pocket would be stopped and they would instead be injected into the lipid bilayer to close the gates. When the drug binding pocket gates bind with the substrate molecules, a significant conformational change follows and a transporting circle is instigated [43,44]. Such an arrangement may help in the formation of “hinges” needed for conformational changes during the transport cycle [45].

## 4. Mechanism by Which P-gp Induces MDR

**Figure 5:**
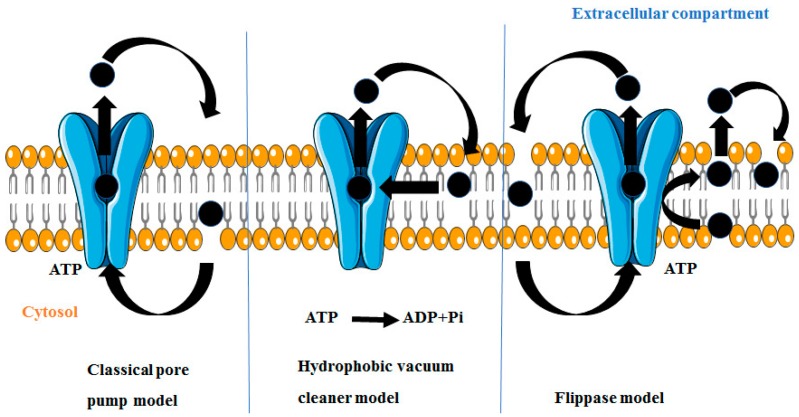
Different functional models of P-gp induced MDR [46,47,48].

Drugs or substrates can move through the cell membrane by filtration, simple diffusion, or by specialized transport, and the preliminary stage in the drug efflux is the identification of the drug by P-gp succeeded by ATP binding and its hydrolysis. Finally, the energy produced is used to efflux substrate out of the cell membrane by a central aperture. Until now, three models of P-gp-mediated drug efflux prevail, namely the “classical pore pump model,” “hydrophobic vacuum cleaner (HVC) model” and “flippase model” as shown in Figure 5.

### 4.1. Classical Pore Pump Model

In the classical-pump model, P-gp constructs a hydrophilic pathway and drugs are exchanged from the cytosol to the extracellular media through them middle of a pore, thus protecting the substrate from the hydrophobic lipid phase [46].

### 4.2. Hydrophobic Vacuum Cleaner Model

According to this model, P-gp binds directly and specifically with the hydrophobic substrates present at the inner side of the plasma membrane and expels them out of the cell by identifying them as xenobiotics. Due to hydrophobic nature of most of these substrates, it has been postulated that initially the substrates balances between the internal aqueous compartment and the inner membrane leaflet before P-gp comes in contact with the substrate. In a second step, ATP hydrolysis leads to conformational alterations of the transporter, which in the process removes substrates from inside to the external aqueous medium [47].

### 4.3. Flippase Model

This model suggests that P-gp interrupts the drug as it travels through the lipid membrane and flips the drug from the inner leaflet (inner side of the plasma membrane) towards outer leaflet (outer side of the plasma membrane) into the extracellular compartment against concentration gradient accompanied by ATP hydrolysis. Presently this is the most accepted model [48].

### 4.4. Mechanisms and Kinetics of P-glycoprotein Efflux

**Figure 6:**
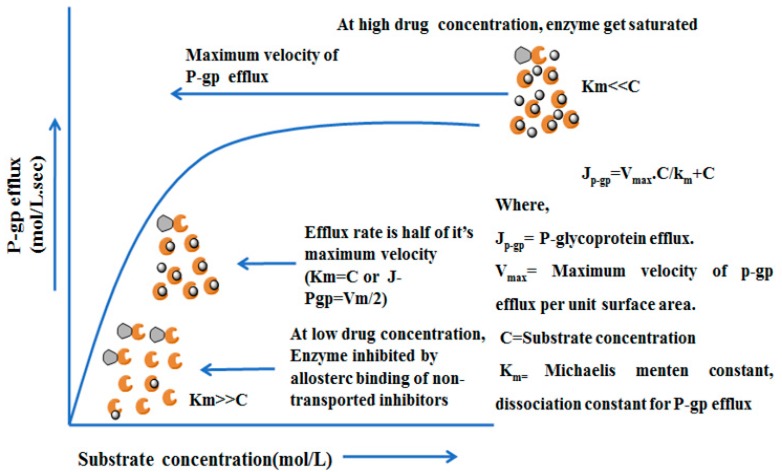
Schematic diagram represents P-gp efflux kinetics [49].

P-gp mediated efflux action follows an active transport mechanism process. In this process, ATP hydrolysis provides the driving force for extrusion of xenobiotics. Generally, the efflux occurs unidirectionally where the xenobiotic is thrown from within the cell into the outer extracellular space and transports only one molecule at a time. Thus, P-gp is also regarded as a uniporter carrier protein. When substrates try to bind with the protein transport site of P-gp for translocation, a competitive inhibitor arrives to vie with the substrate drug for discharge and occupy all the accessible protein transport sites leaving no opportunity for the P-gp and substrate interaction whereas on the other side non-competitive inhibitors neither bind to the transport sites nor are translocated by the efflux pumps and therefore are as well-known as non-transported inhibitors. They rather bind to an all osteric modulatory site and non-competitively inhibit the protein efflux. The mechanism of action of the competitive and non-competitive (non-transported) inhibitors apart from the P-gp efflux kinetics is depicted in Figure 6, as non-linear dose dependent kinetics, mixed-order kinetics or Michaelis-Menten kinetics [49].

## 5. P-Glycoprotein Inhibition

The nature of interaction of a particular compound with a receptor or protein detects it either as a P-gp inhibitor or substrate or an inducer. Based on their affinity, specificity and toxicity, P-gp inhibitors are classified into three generations (Table 1).

The first generation inhibitors are metabolites that already have some proven clinical use, viz verapamil (calcium channel blocker) and cyclosporin A (immunosuppressive drug), and were then tested against P-gp and was found to possess enzyme inhibitory activity. These drugs require high concentrations to inhibit P-gp and, thus were not approved as P-gp inhibitors [50,51]. Second-generation inhibitors are compounds without any prior reported curative potential and have a greater affinity for P-gp than first-generation inhibitors. The problem with these metabolites is that they are rapidly metabolized by the enzyme CYPA4, thereby changing their pharmacokinetics and decreasing their efficacy. It is pertinent to mention that these inhibitors are structured to have decreased toxicity than the first-generation inhibitors, despite inheriting some of the undesirable toxic characteristics which limit their pharmacological use [52,53]. Third generation inhibitors were discovered using the concept of combinatorial chemistry and structure-activity relationship (SAR) studies in order to identify P-gp inhibitors having high specificity and low toxicity. These P-gp inhibitors are approximately 10 times more potent than previous generations of inhibitors. The enzyme CYPA4 does not inhibit these compounds and therefore does not show changed pharmacokinetics [54].

P-gp inhibitors belonging to any one of the three generations exercise their effect by the following mechanisms (Table 2): (1) altering ATP hydrolysis pathway; (2) alteration in P-gp expression; and (3) reversible or competitive inhibition for a binding site. One of the most routine strategies inherited by conventional P-gp inhibitors is competition for drug binding sites. The presence of multiple binding sites on P-gp however makes it much difficult to design targeted inhibitors. Additionally, the various negative factors that don’t allow success are: (1) presence of unpredictability in the response rate related with P-gp inhibitors; (2) occurrence of drug induced toxicity due to pharmacokinetic interaction between the P-gp inhibitor and the other drugs; (3) altered metabolism or excretion; and (4) altering the basic role of drug expulsion by P-gp thus increasing the toxicity level of a co-administered drug in healthy tissues. Therefore, there is a dire need to identify new, more effective and non-toxic P-gp inhibitors [55,56].

### 5.1. Herbal Modulation of P-gp

Inhibition of P-gp by herbal constituents is an innovative technique for reversing drug resistance in chemotherapies [57]. Therefore, many efforts are currently being done to find natural compounds from plant sources that inhibit P-gp, reverse the MDR phenotype and sensitize the target cells to conventional chemotherapy without undesirable toxicological effects [58,59].

**Figure 7:**
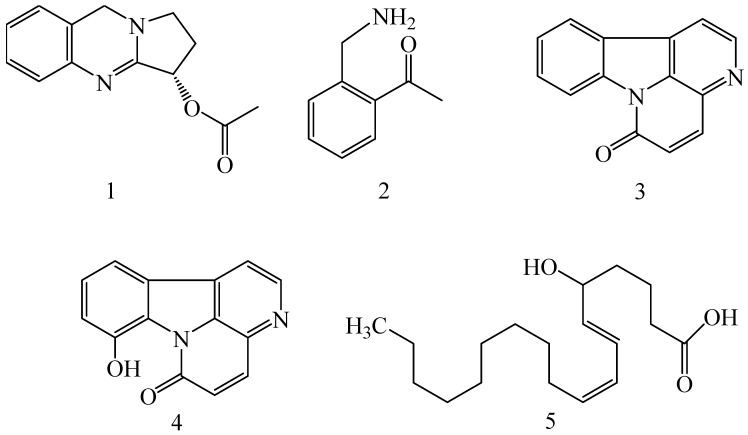

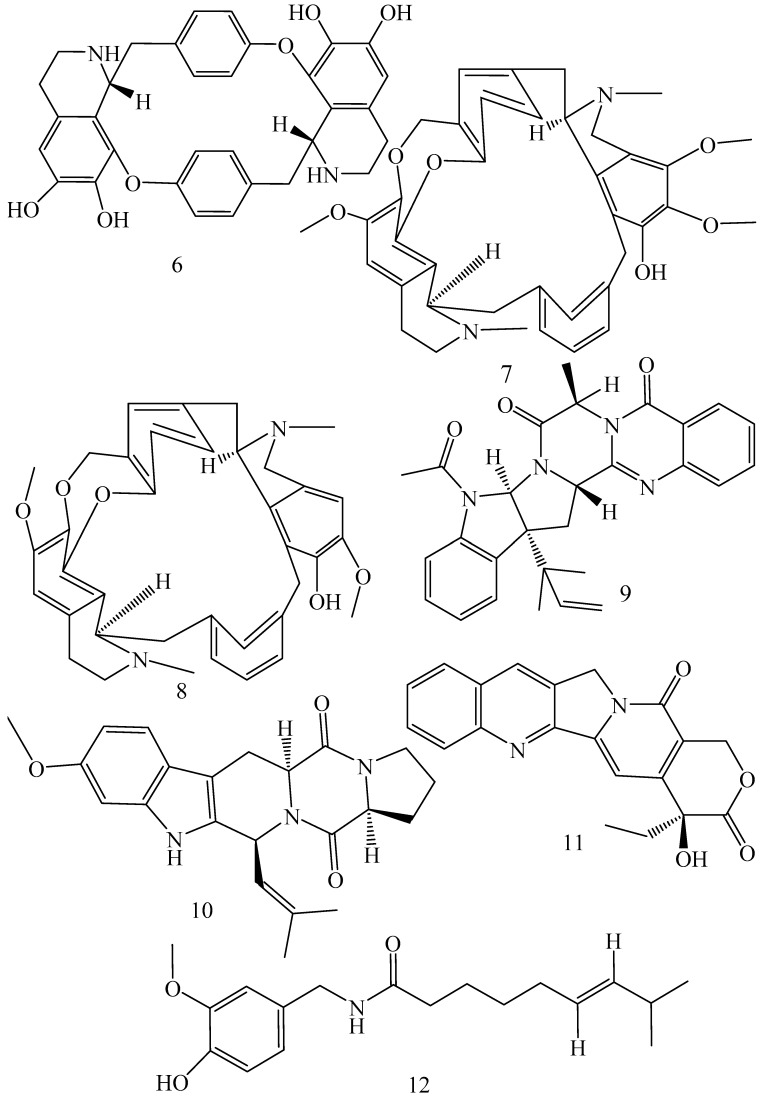

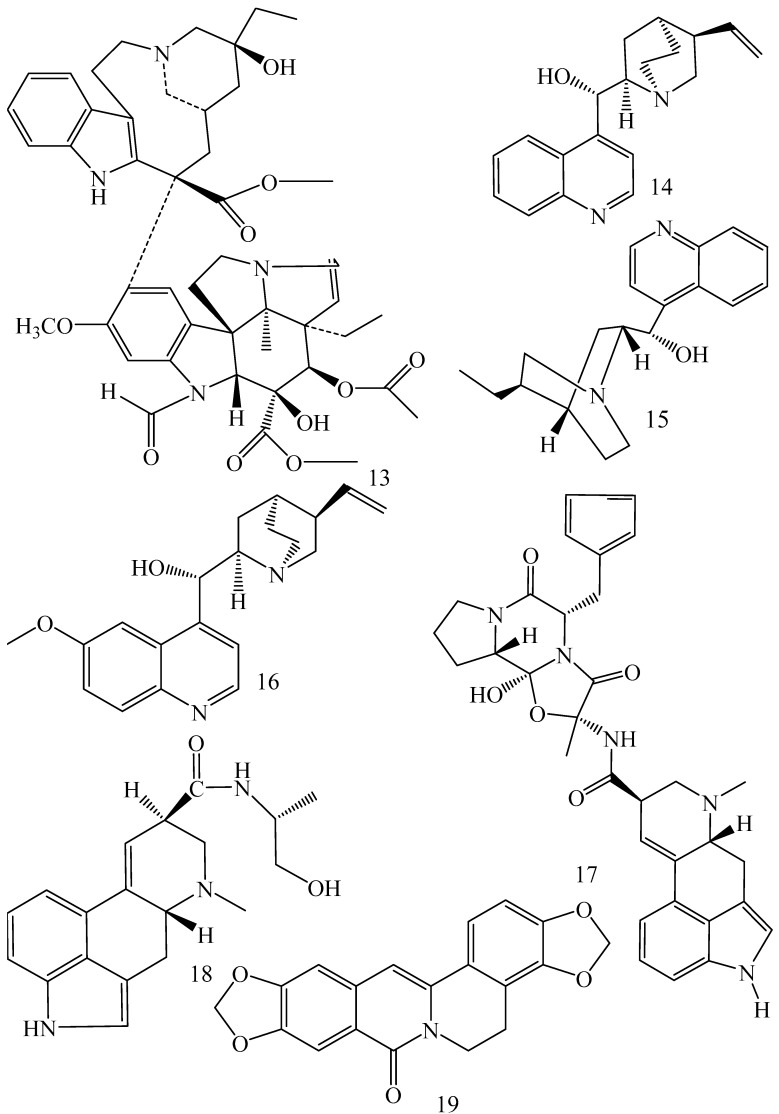

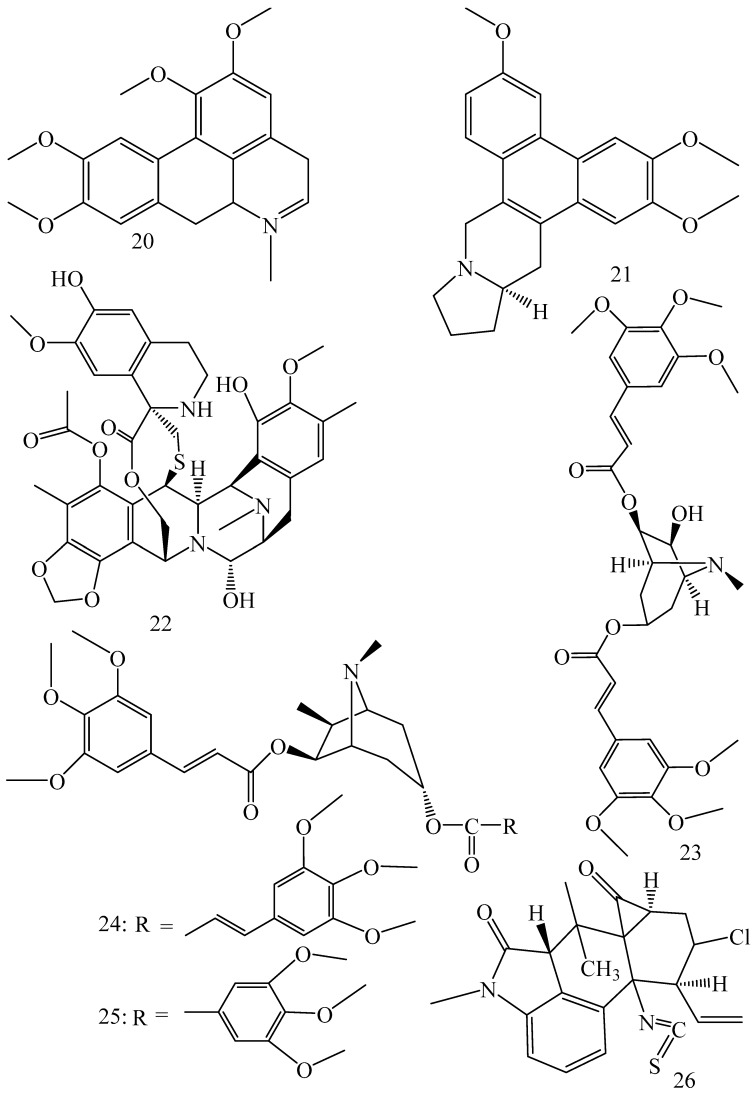

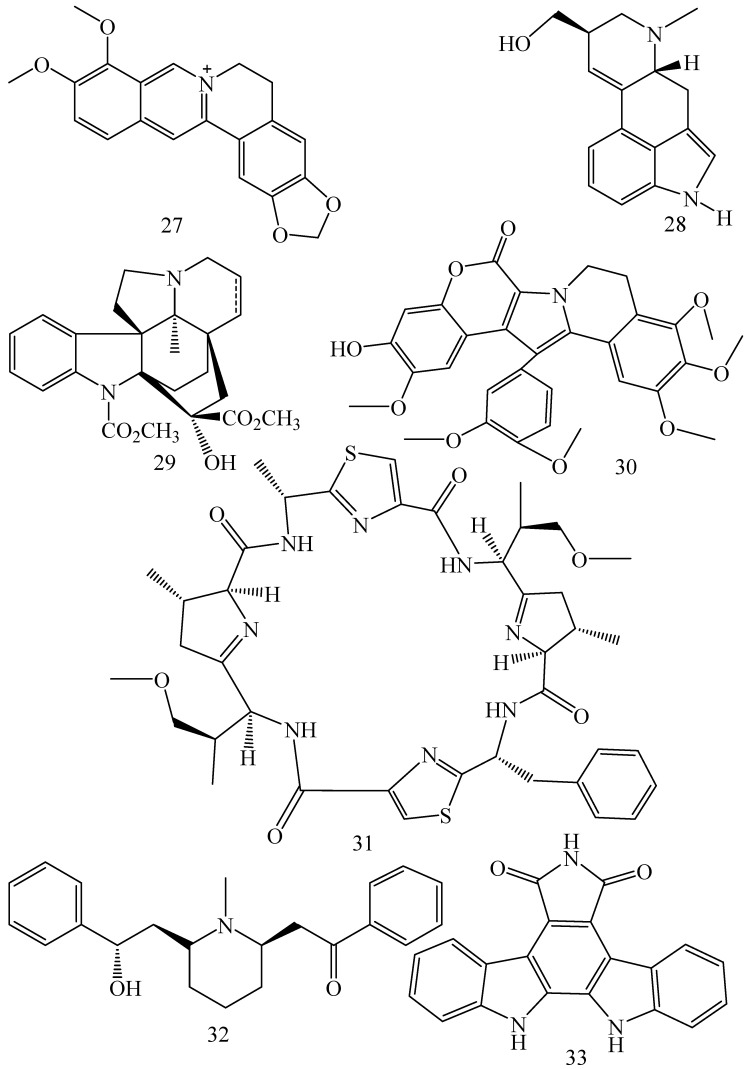

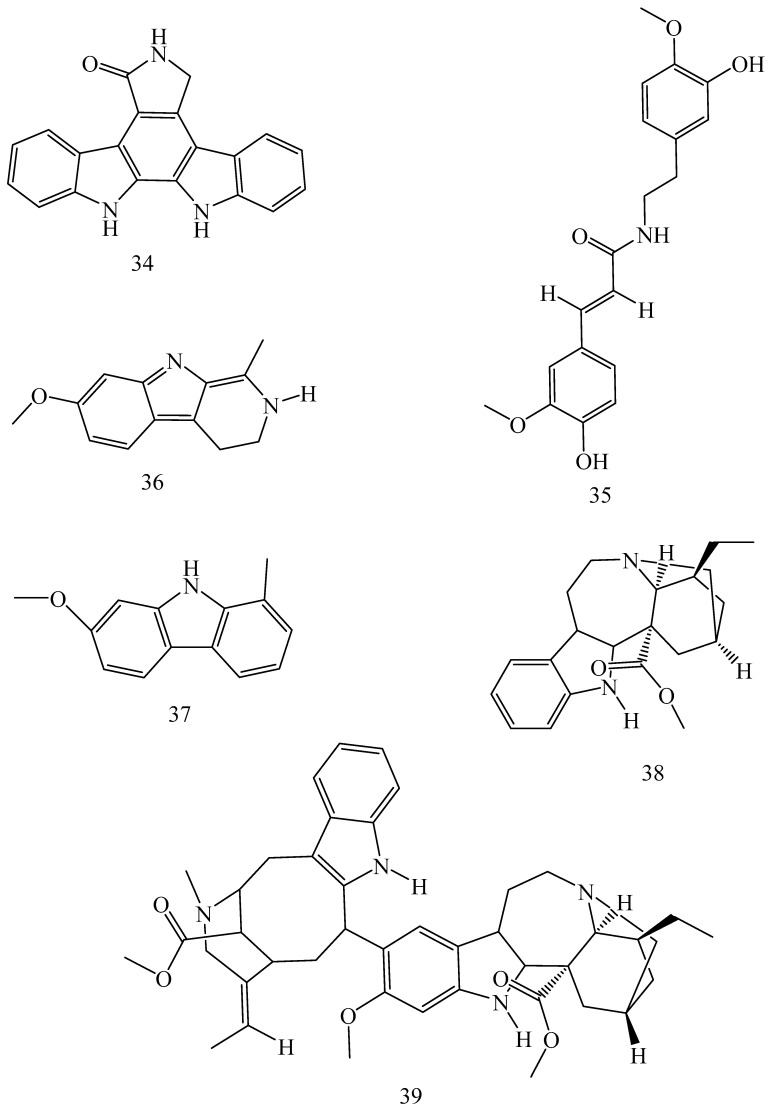

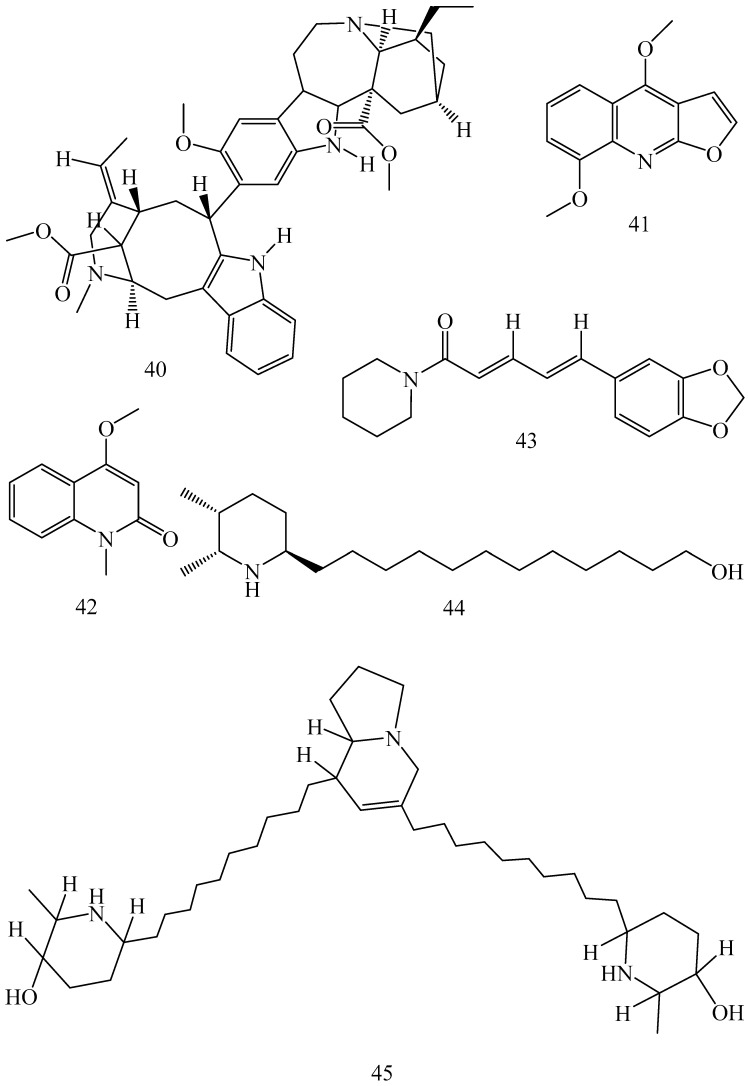

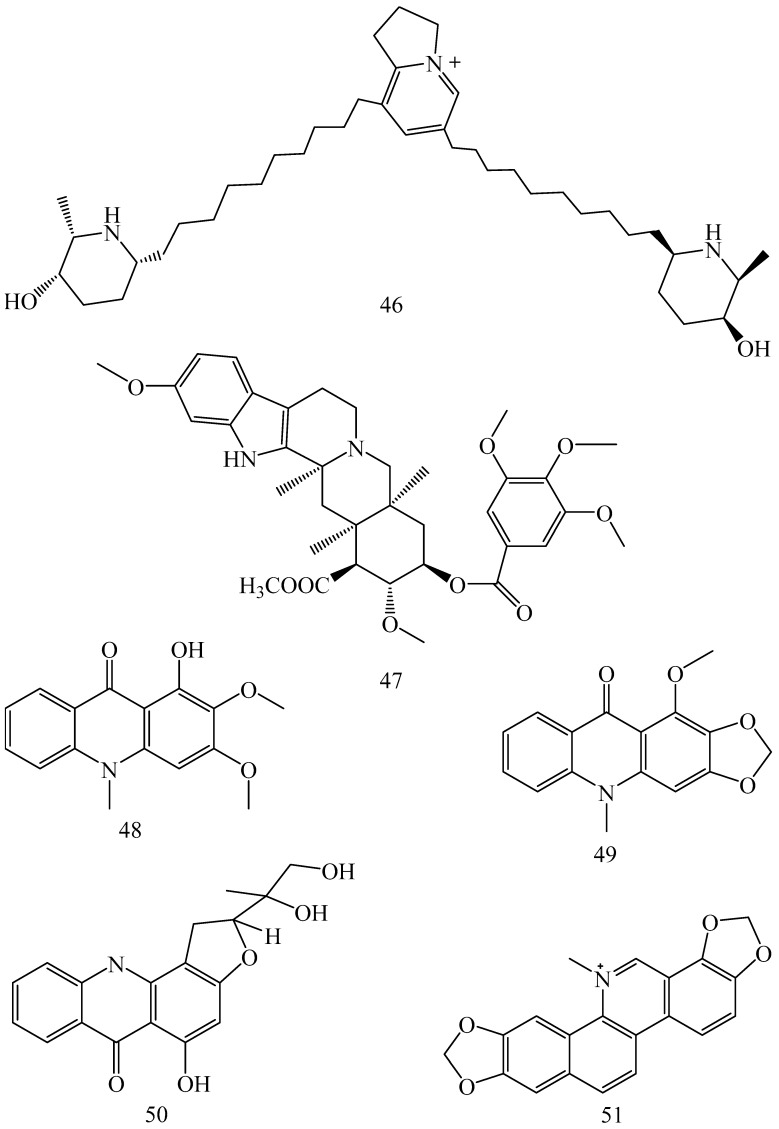

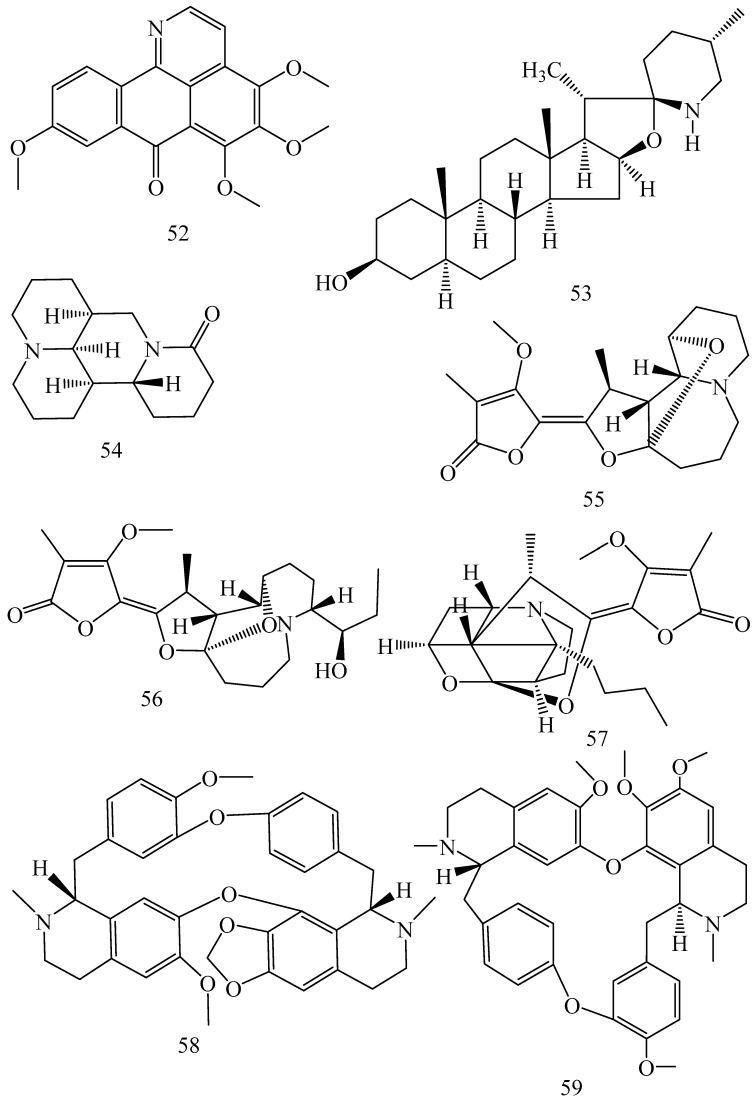

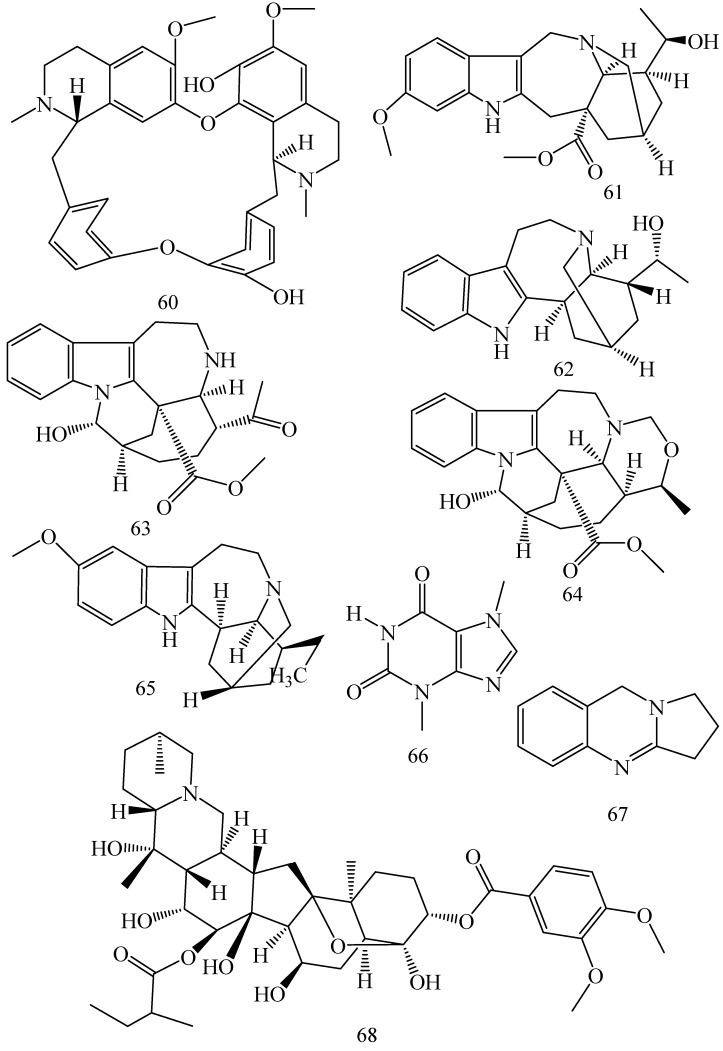

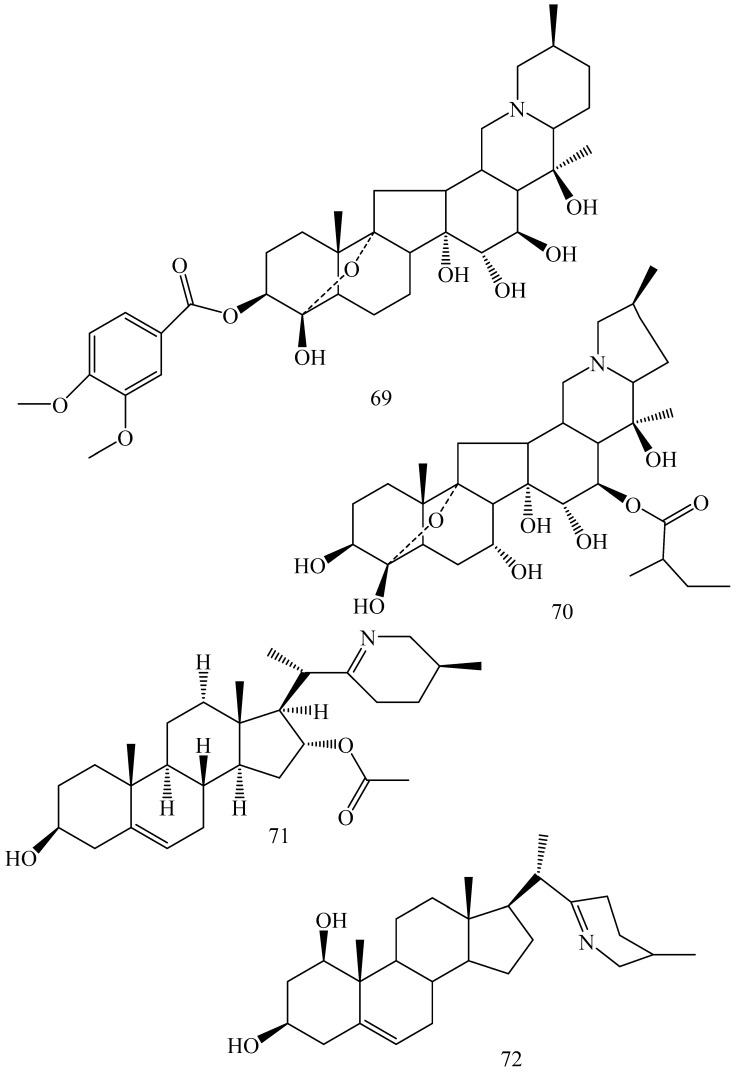

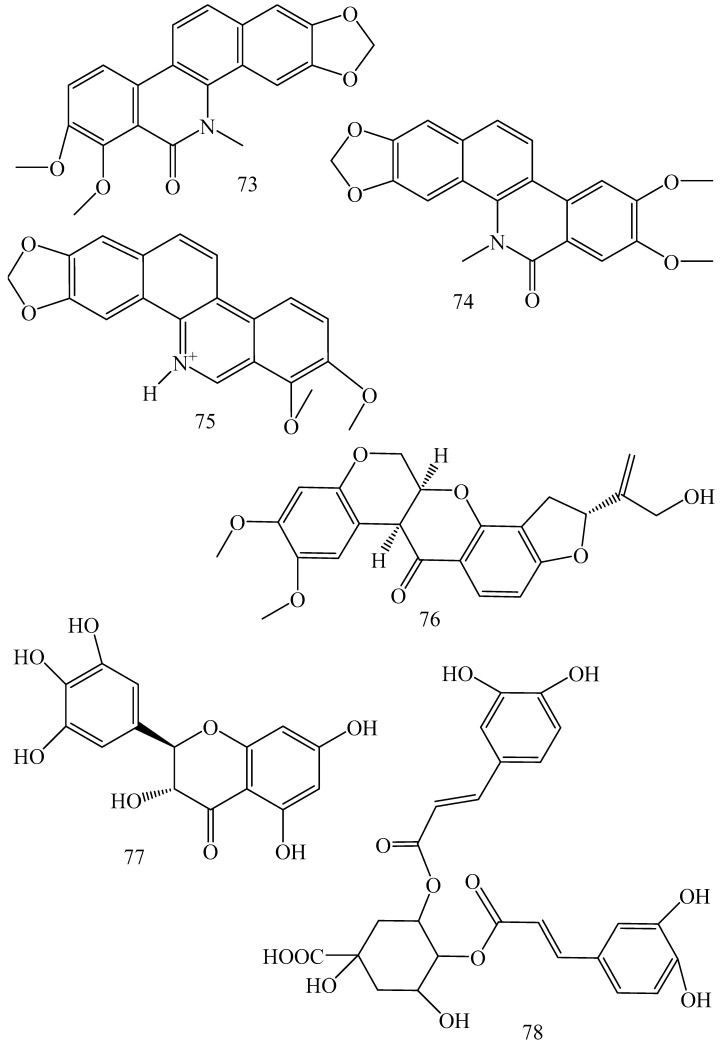

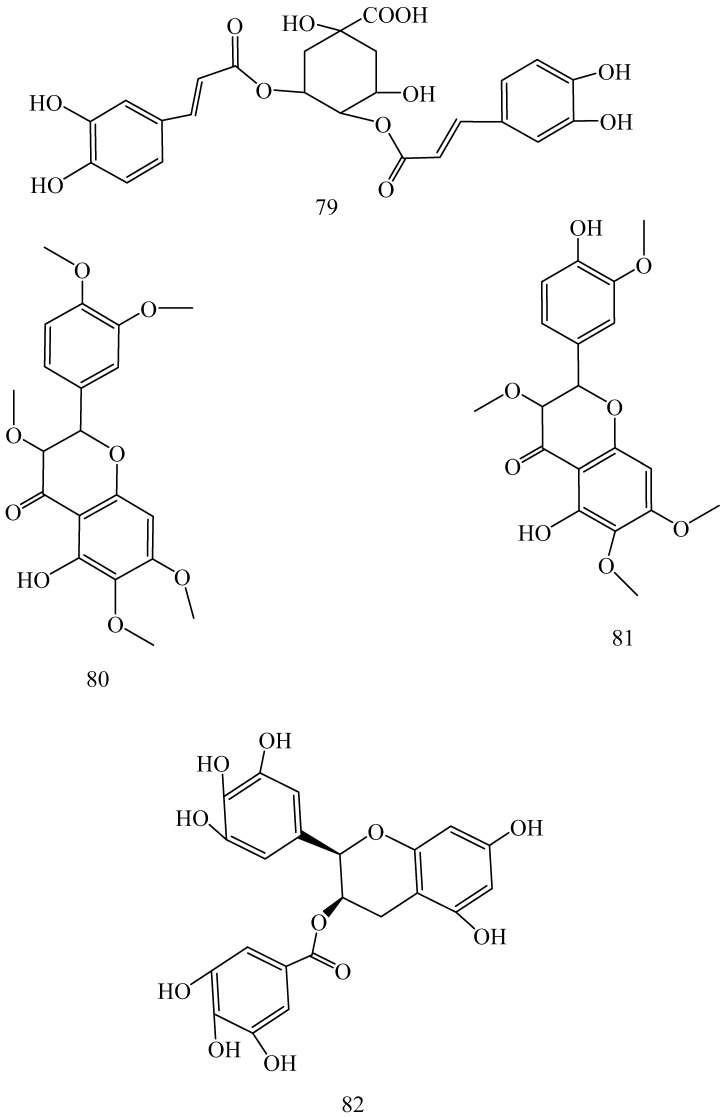

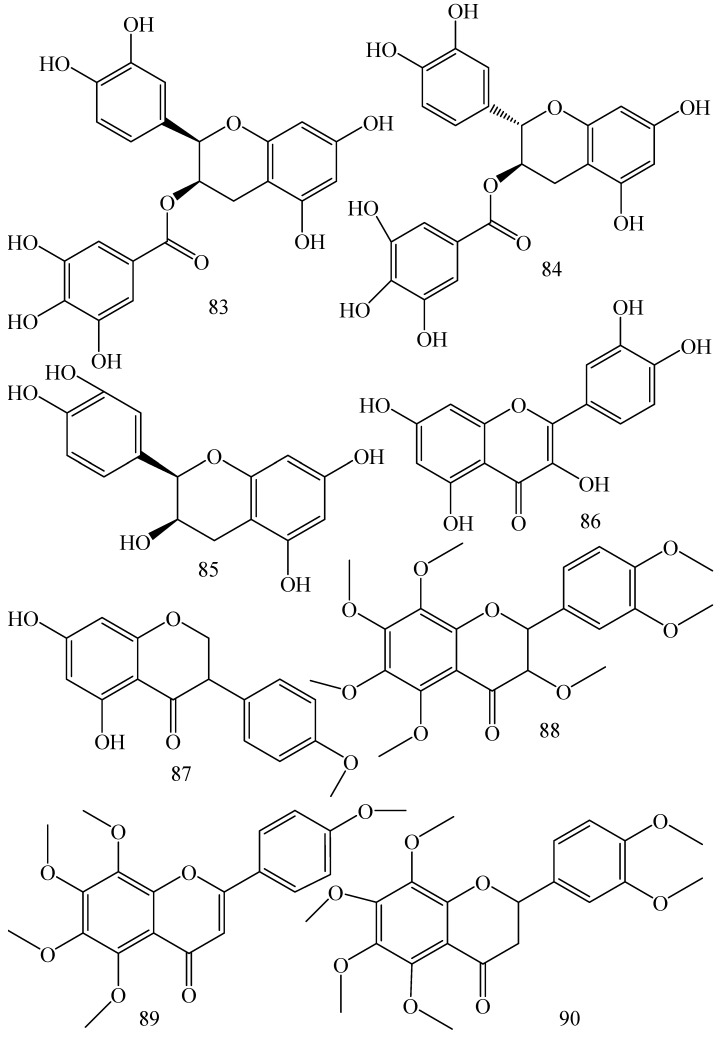

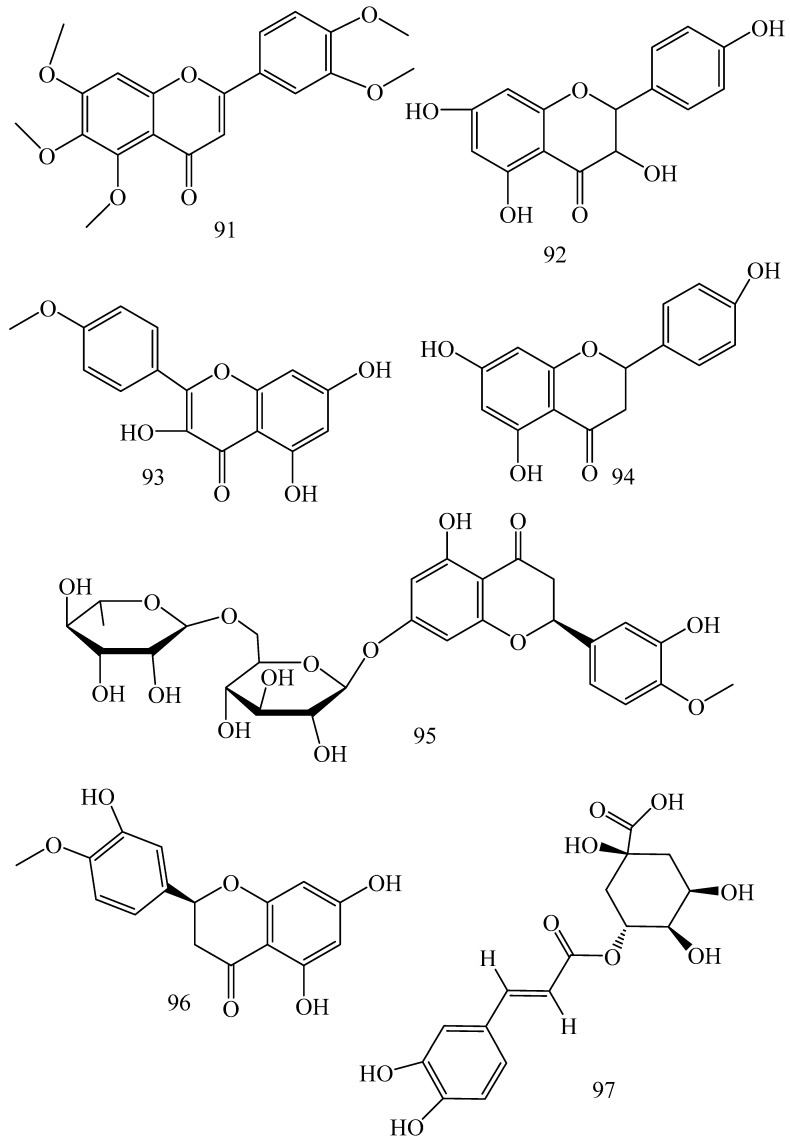

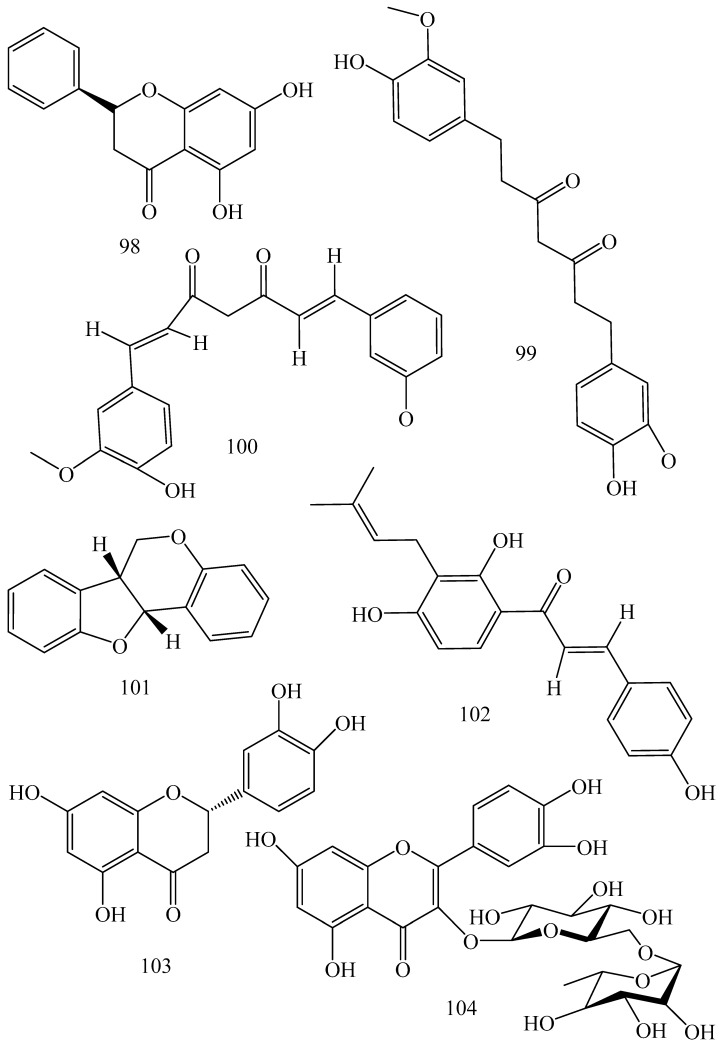

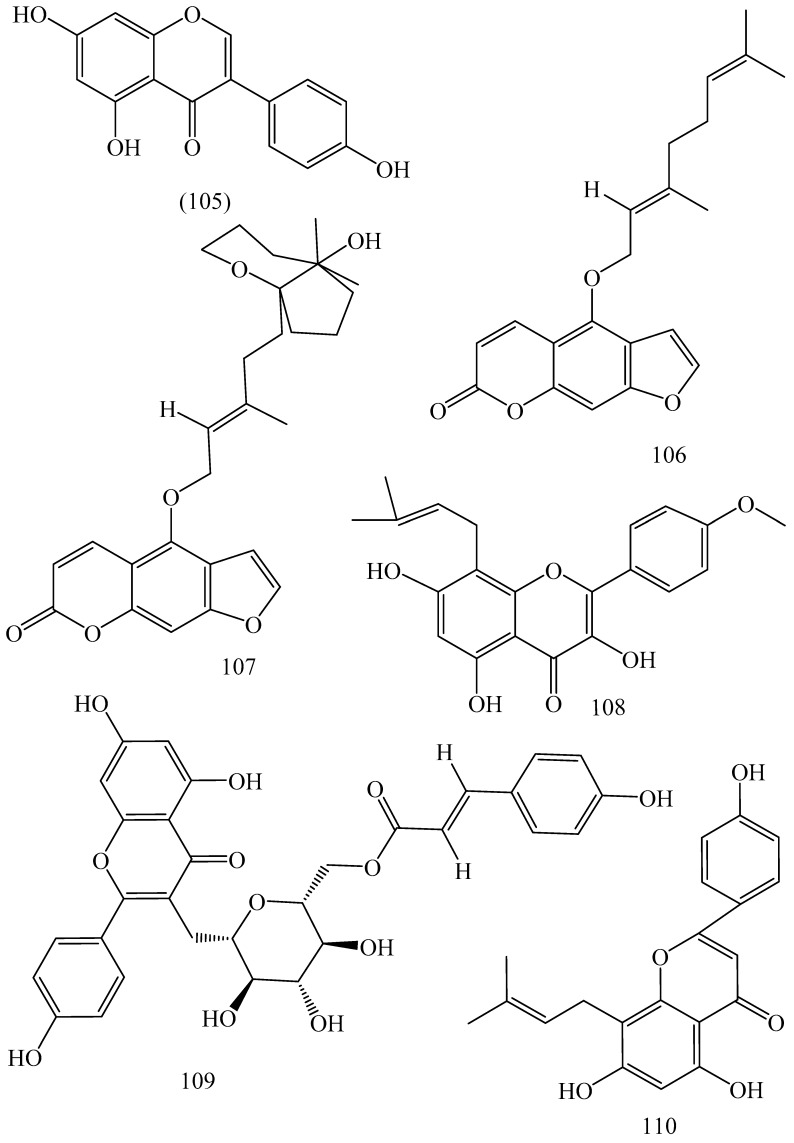

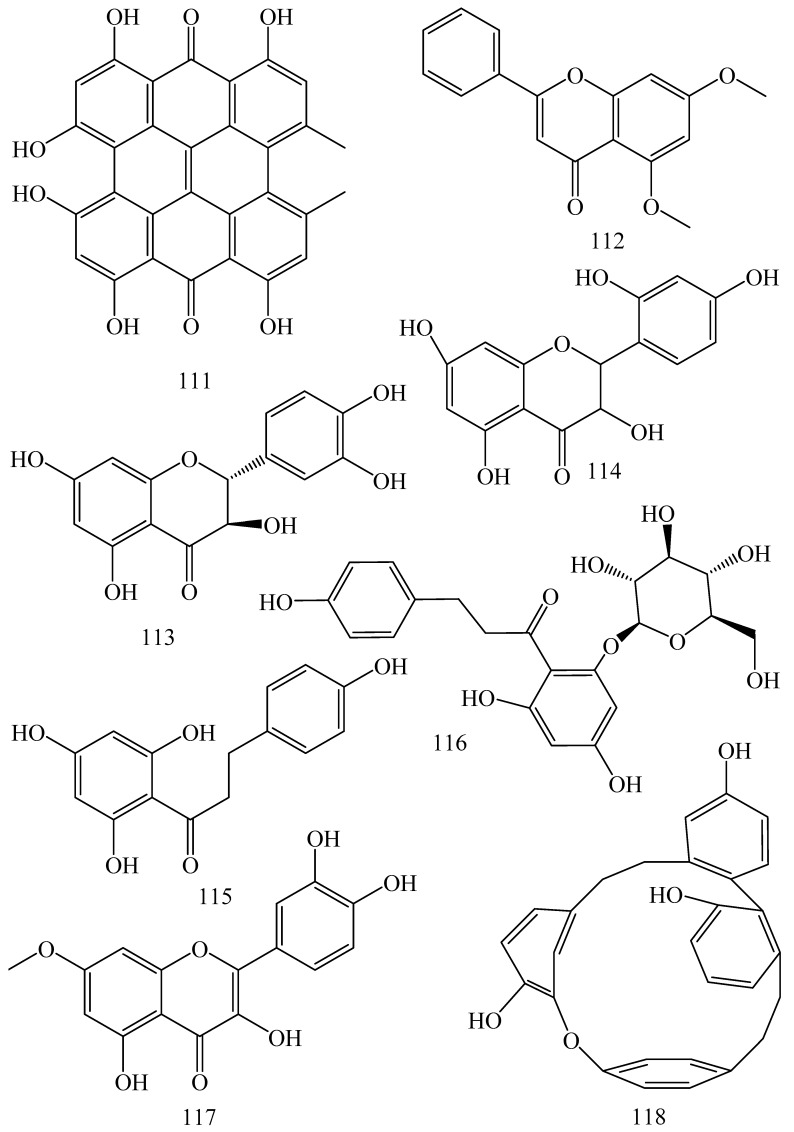

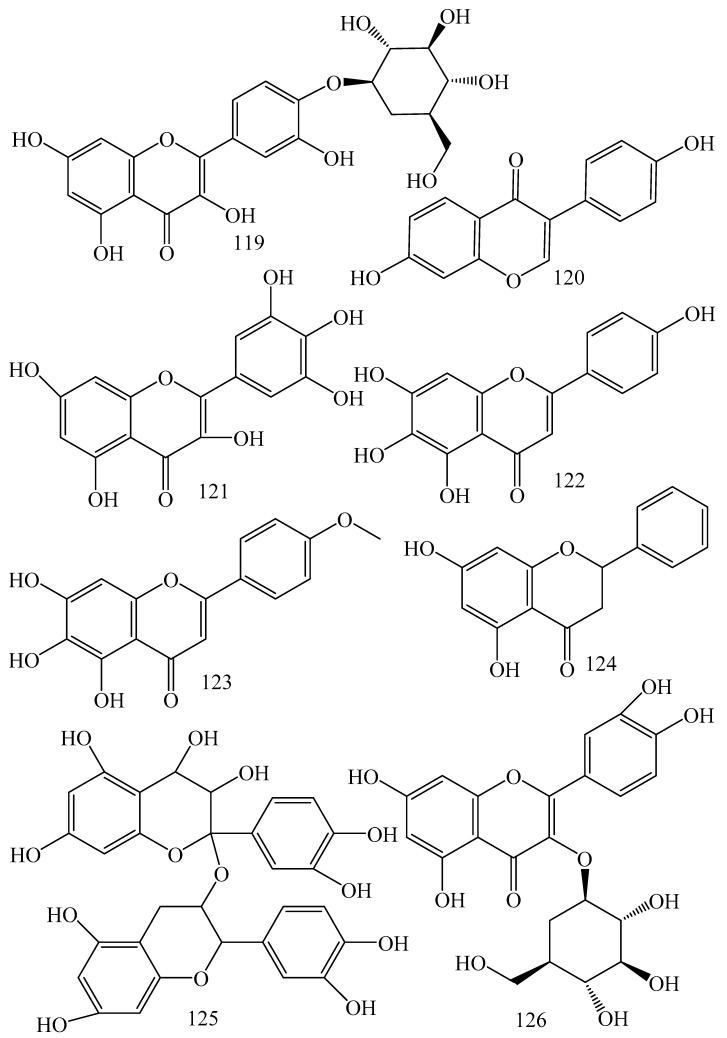

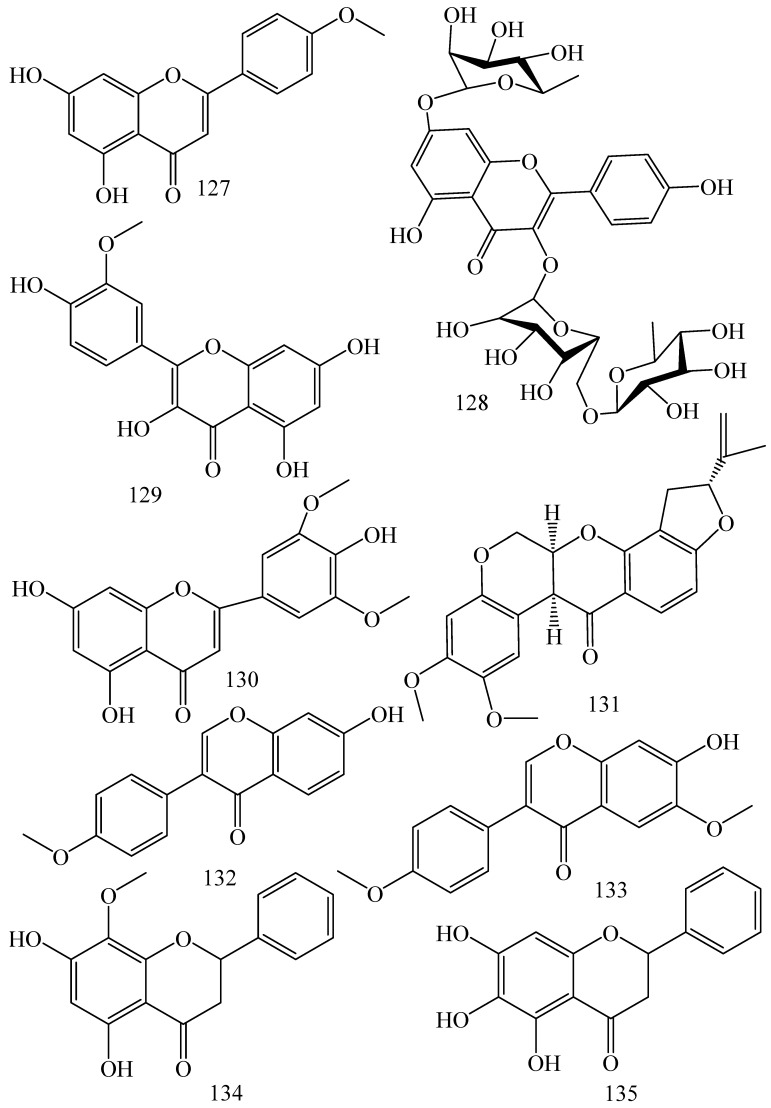

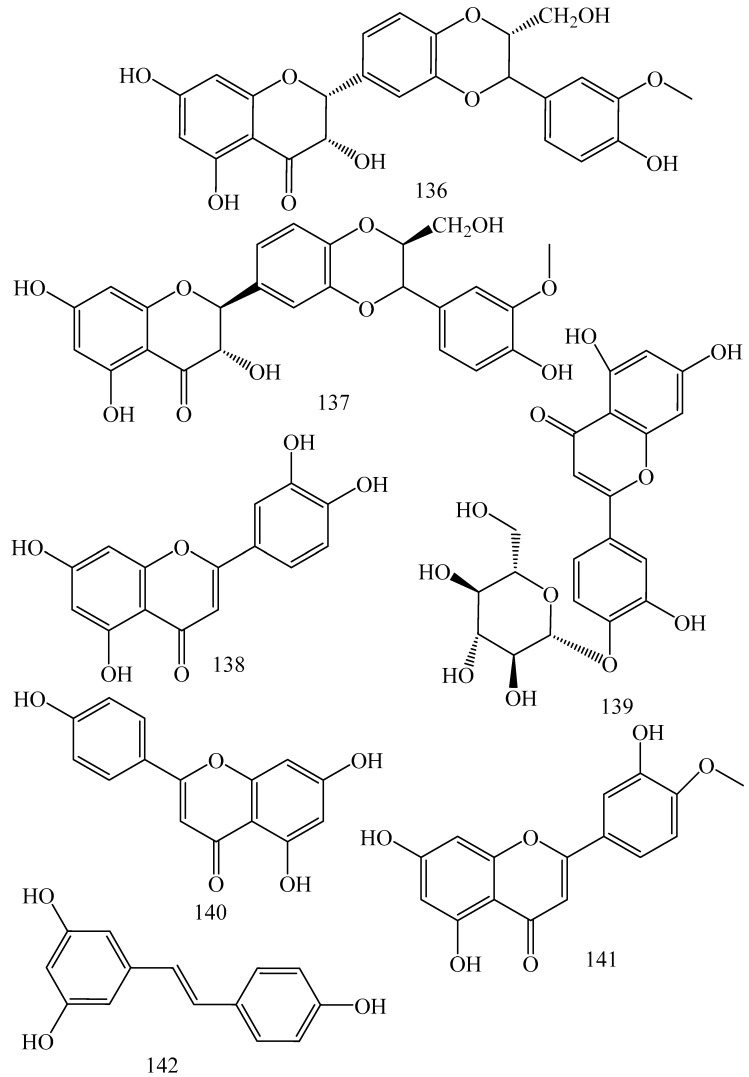

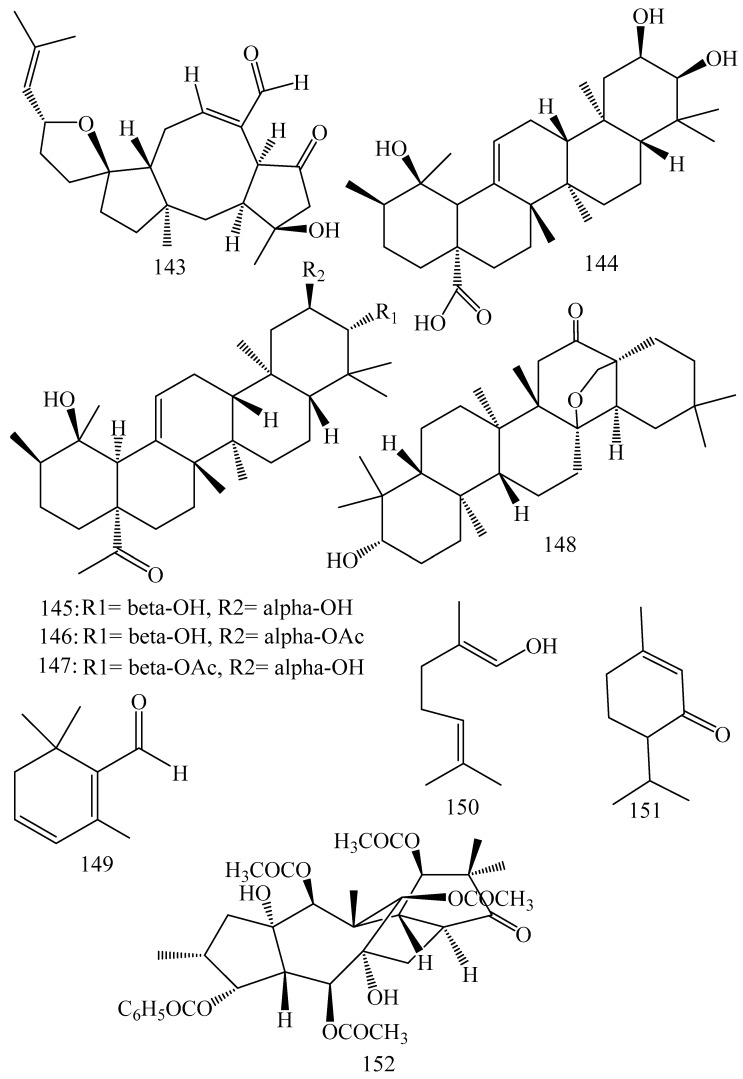

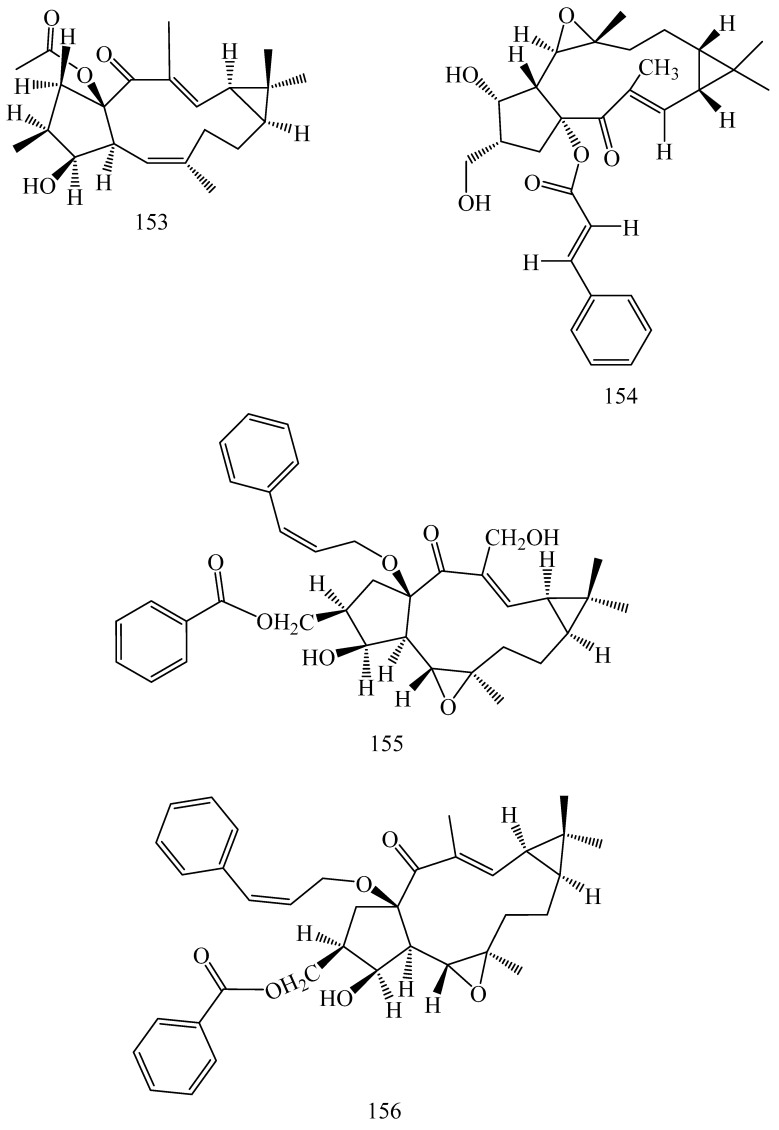

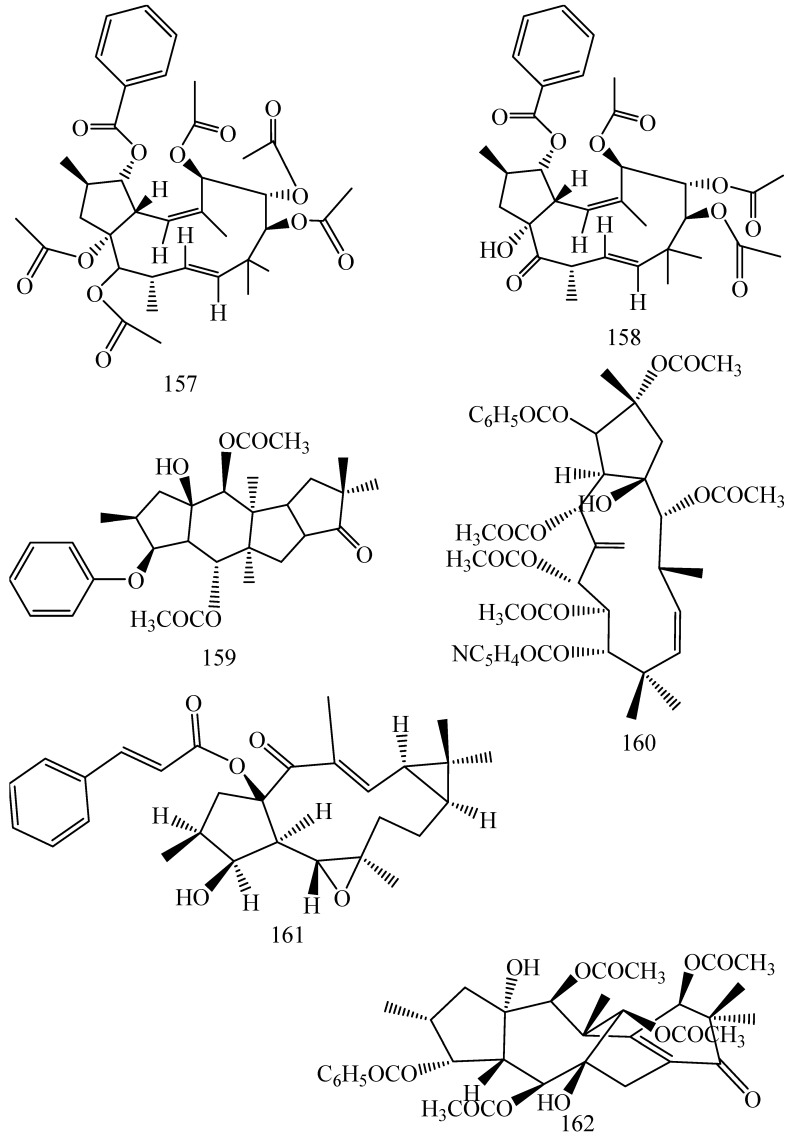

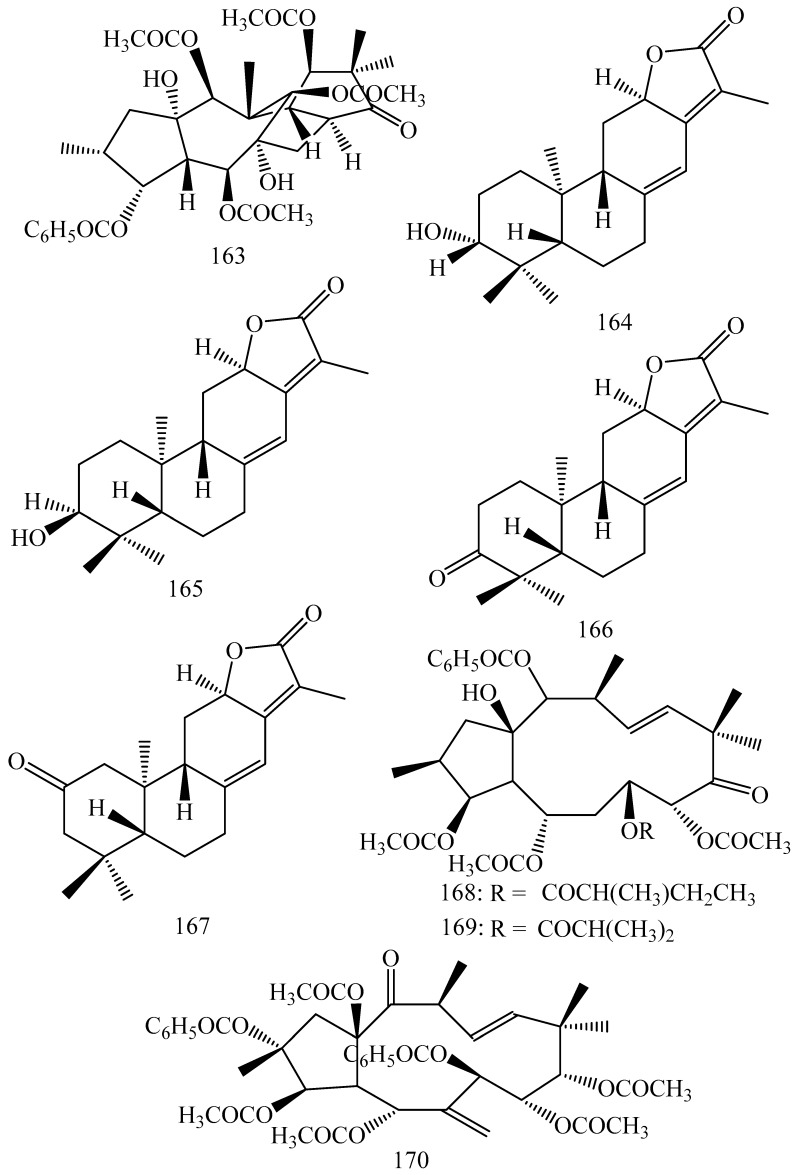

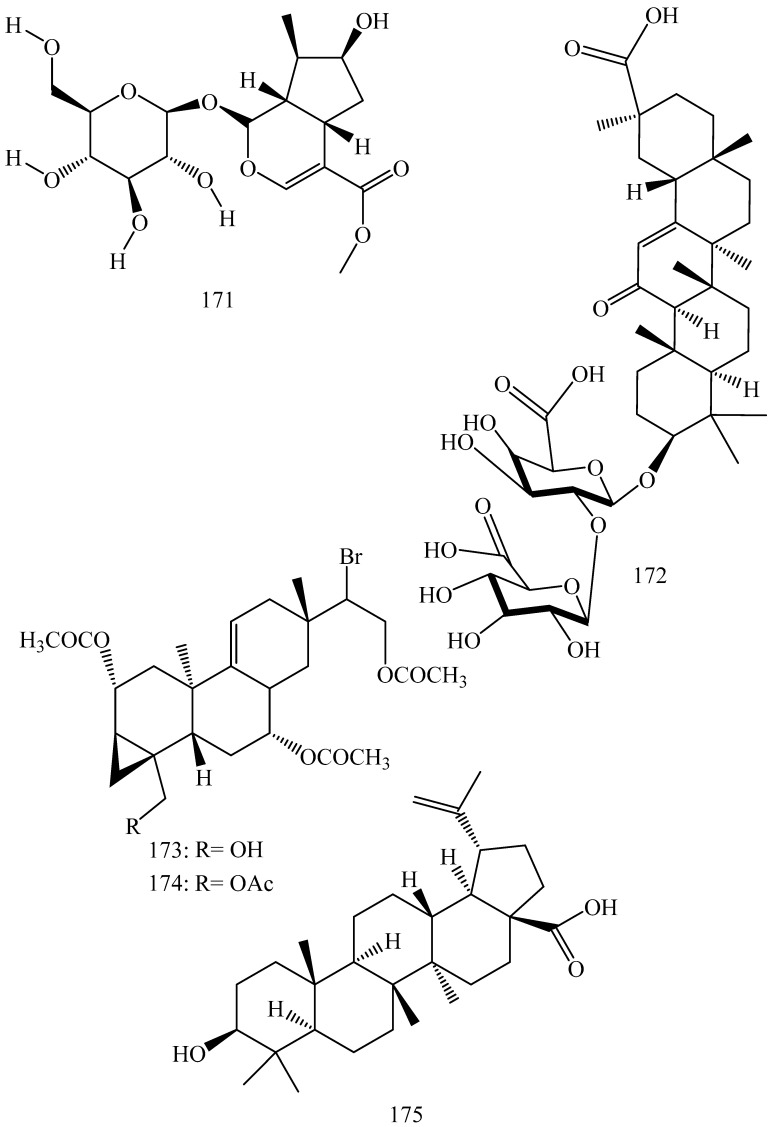

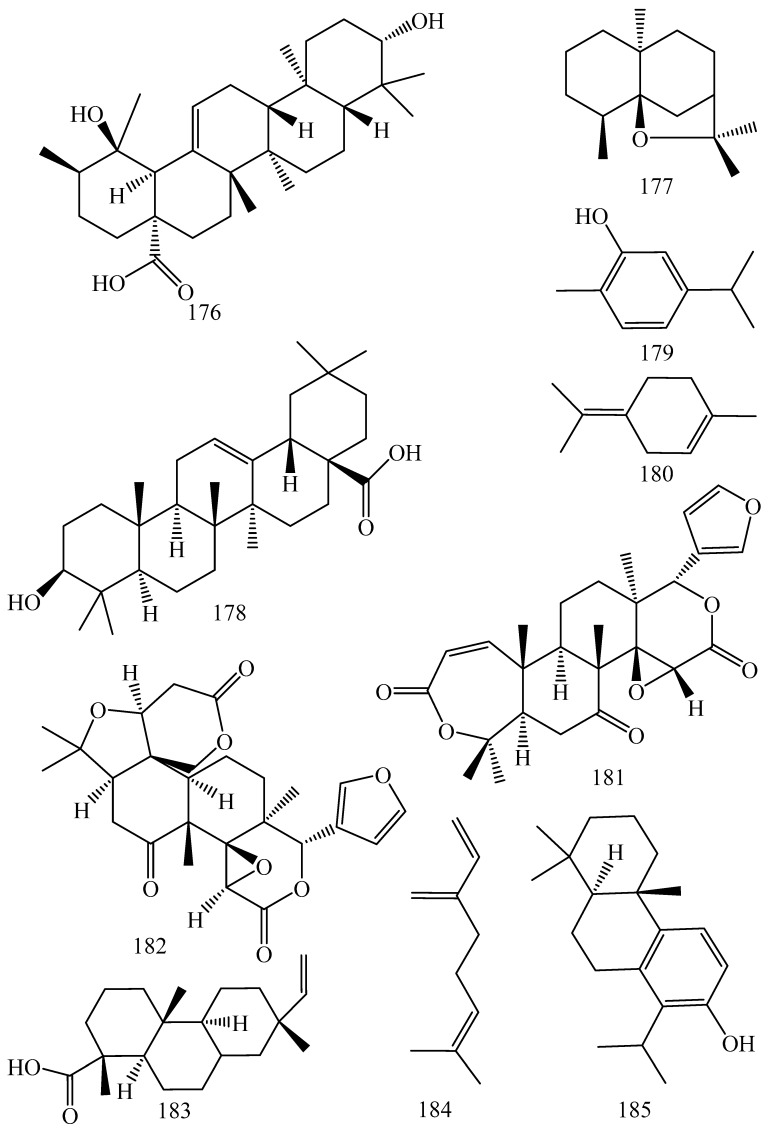

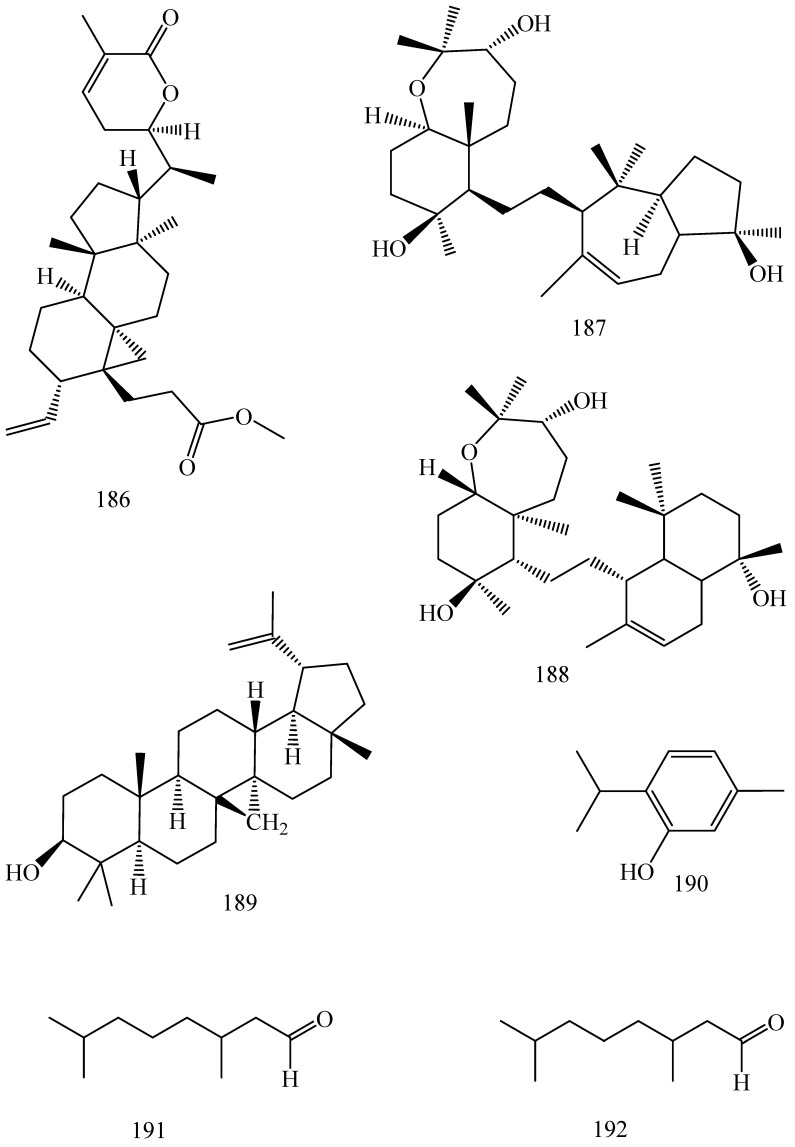

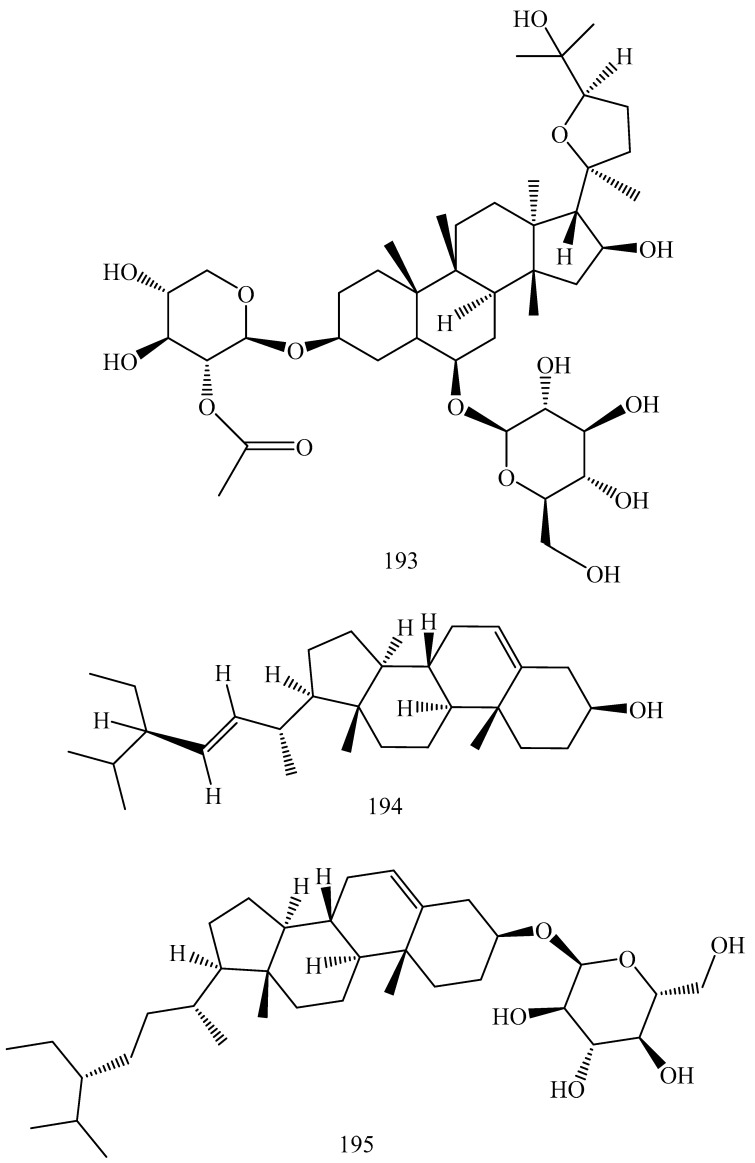

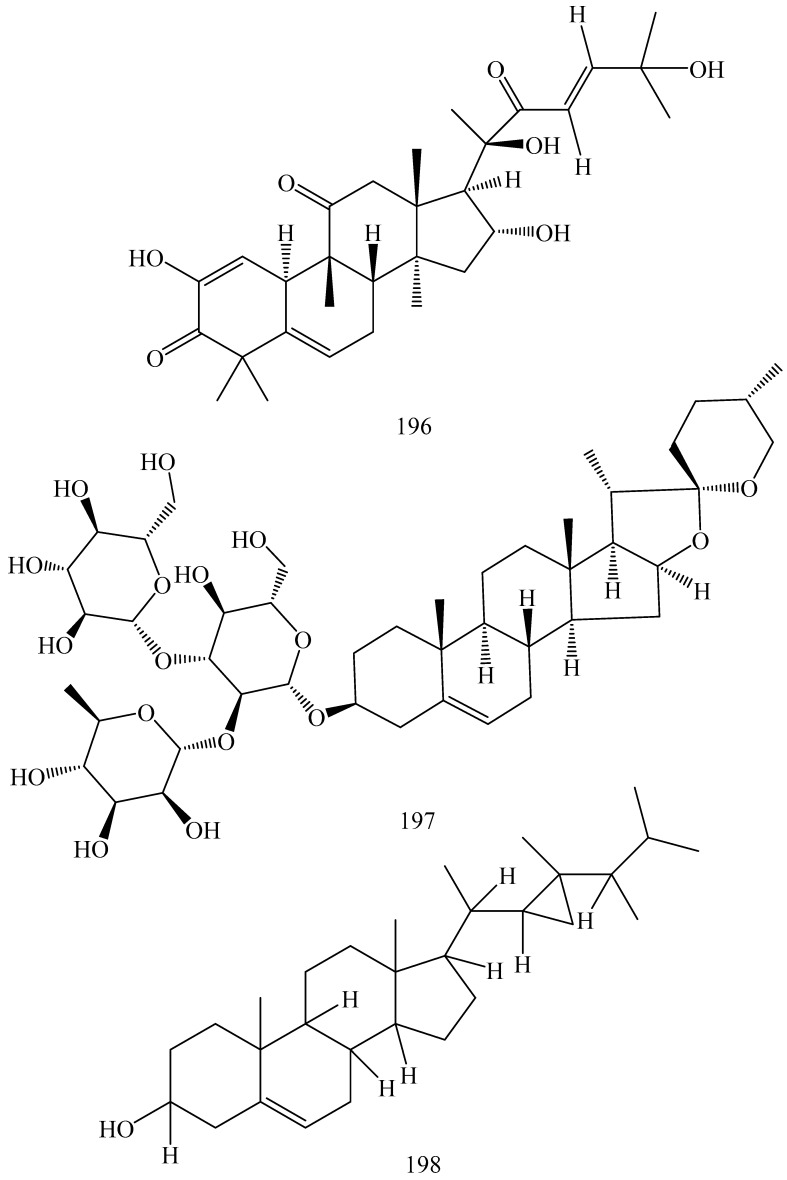

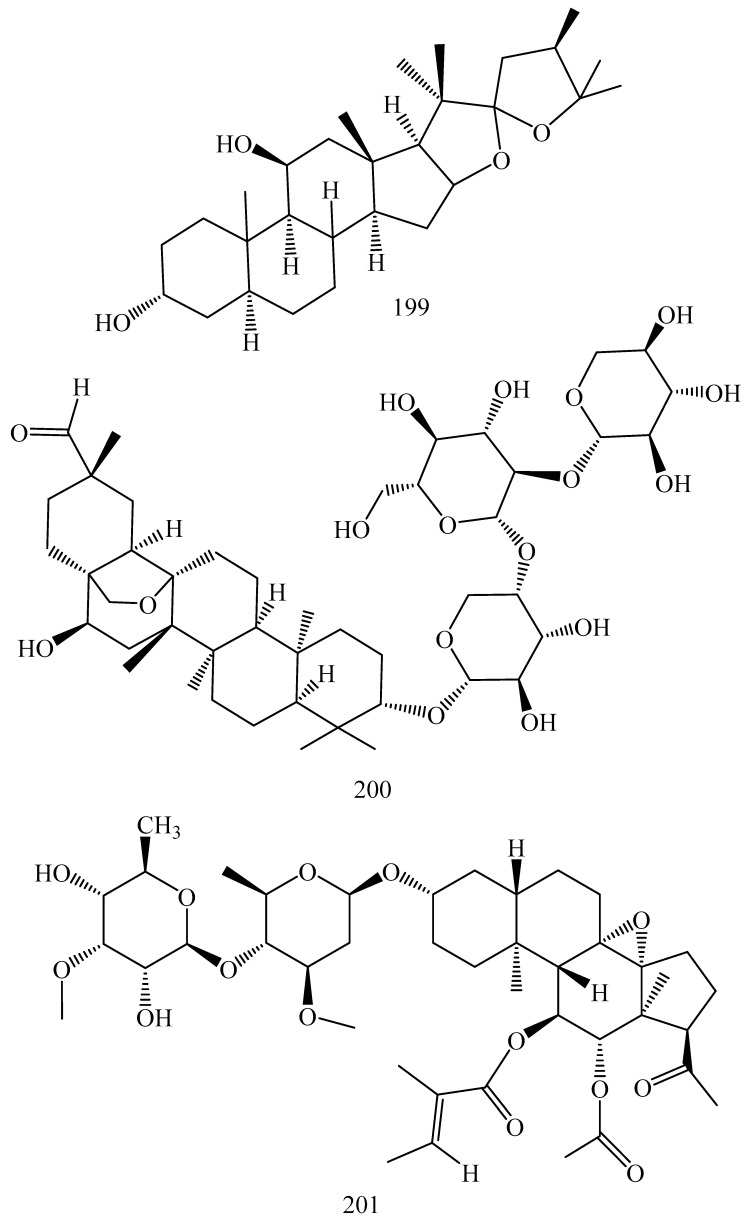

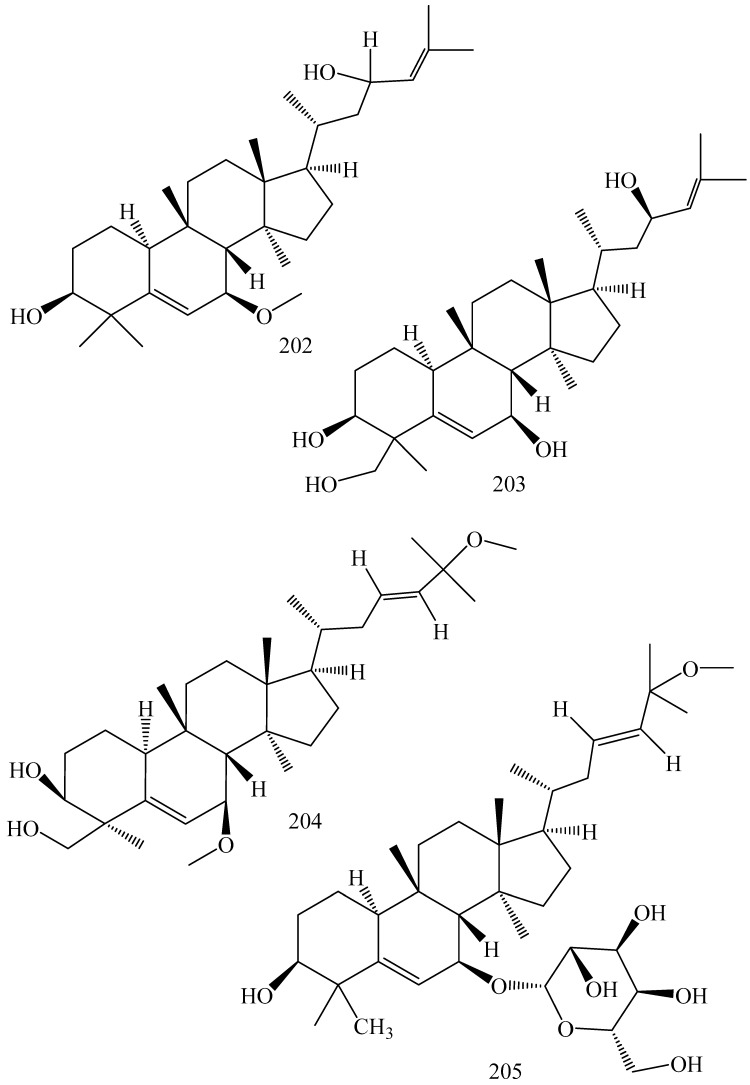

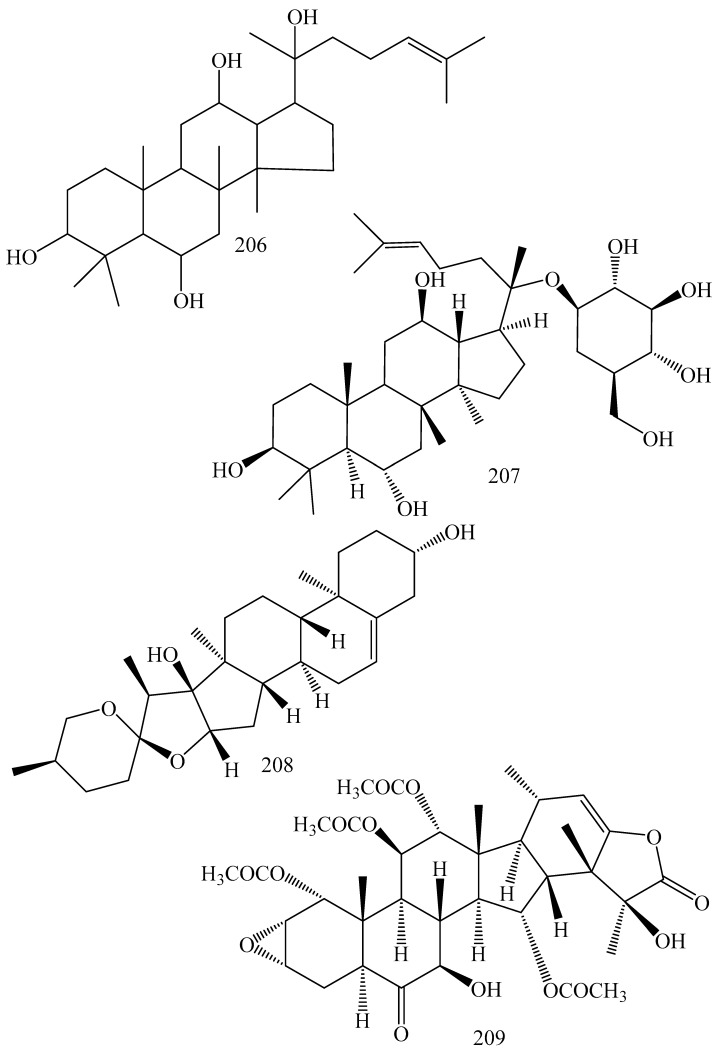

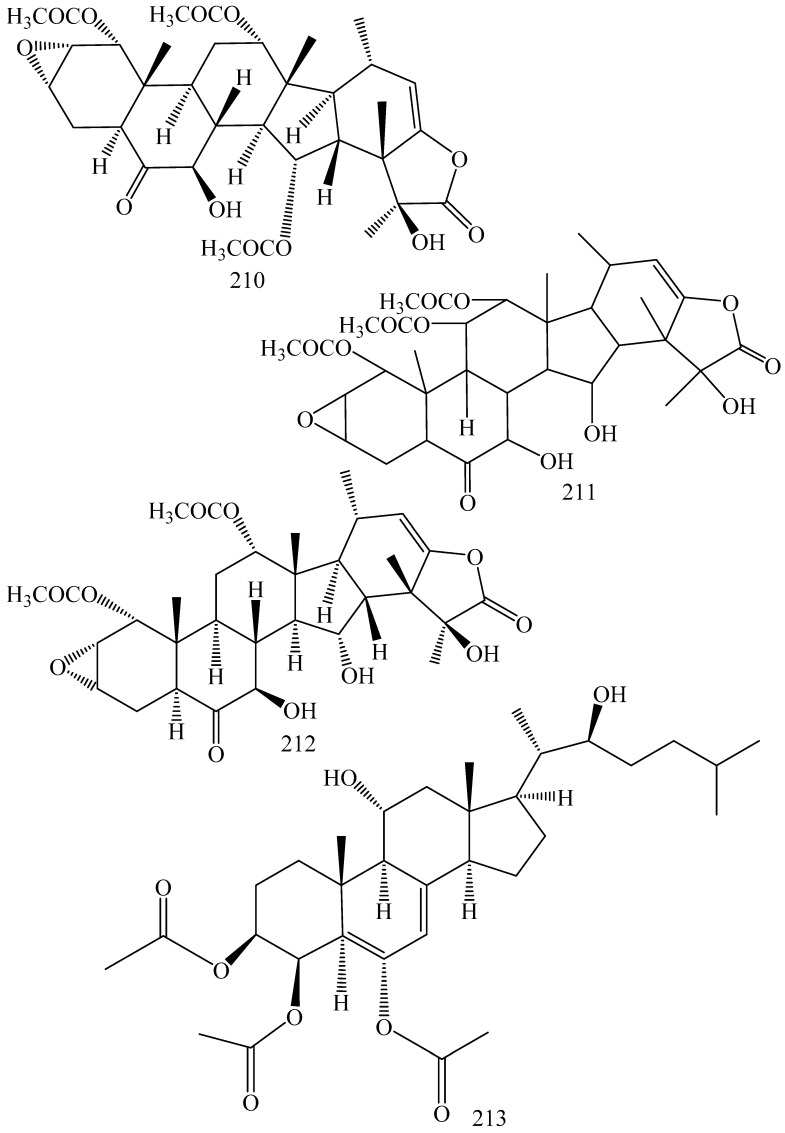

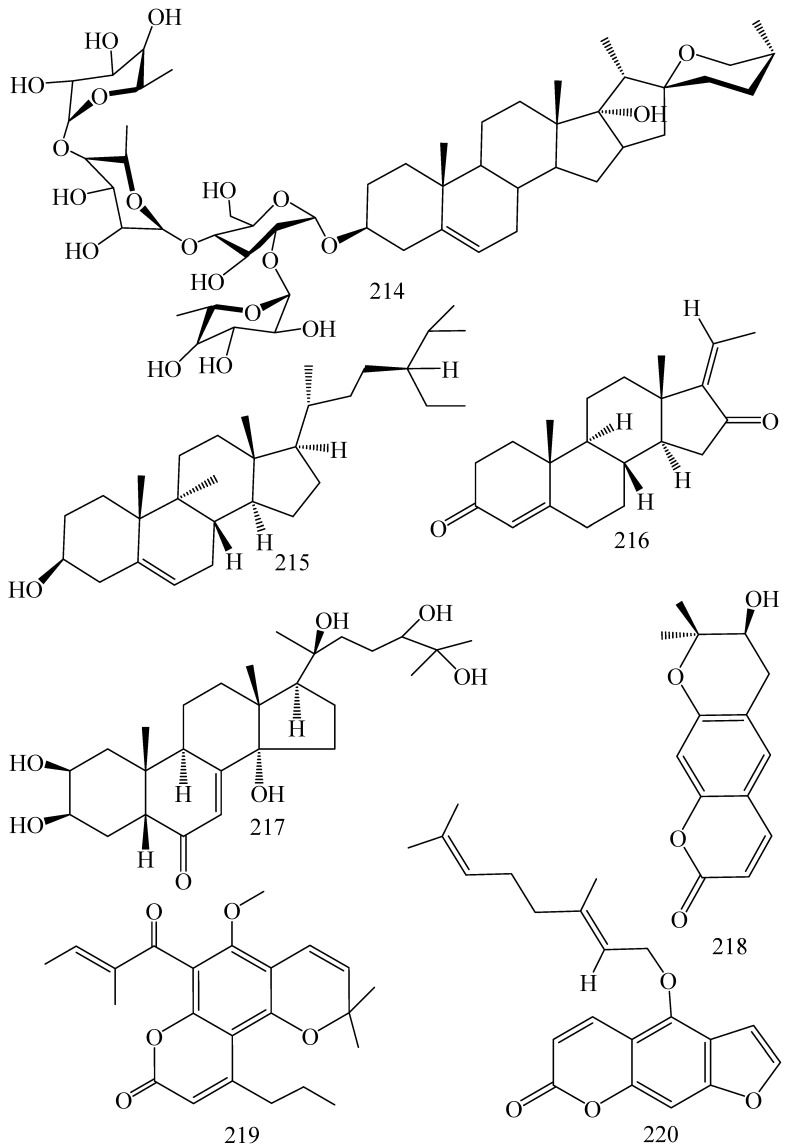

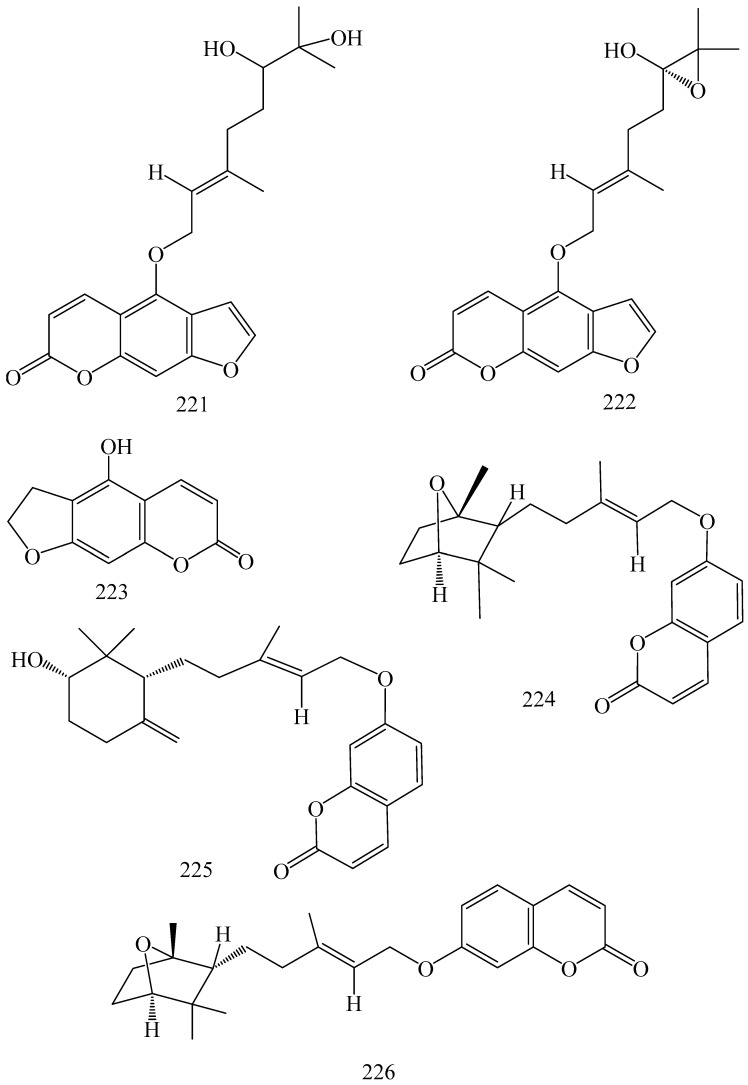

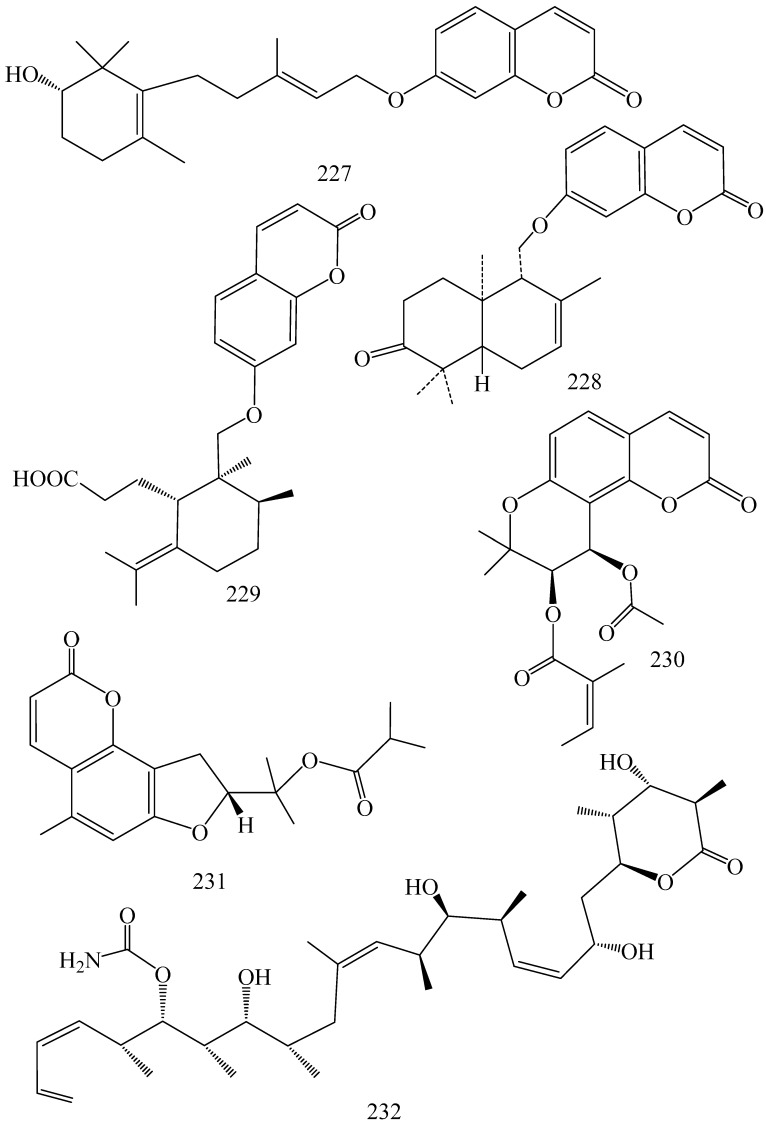

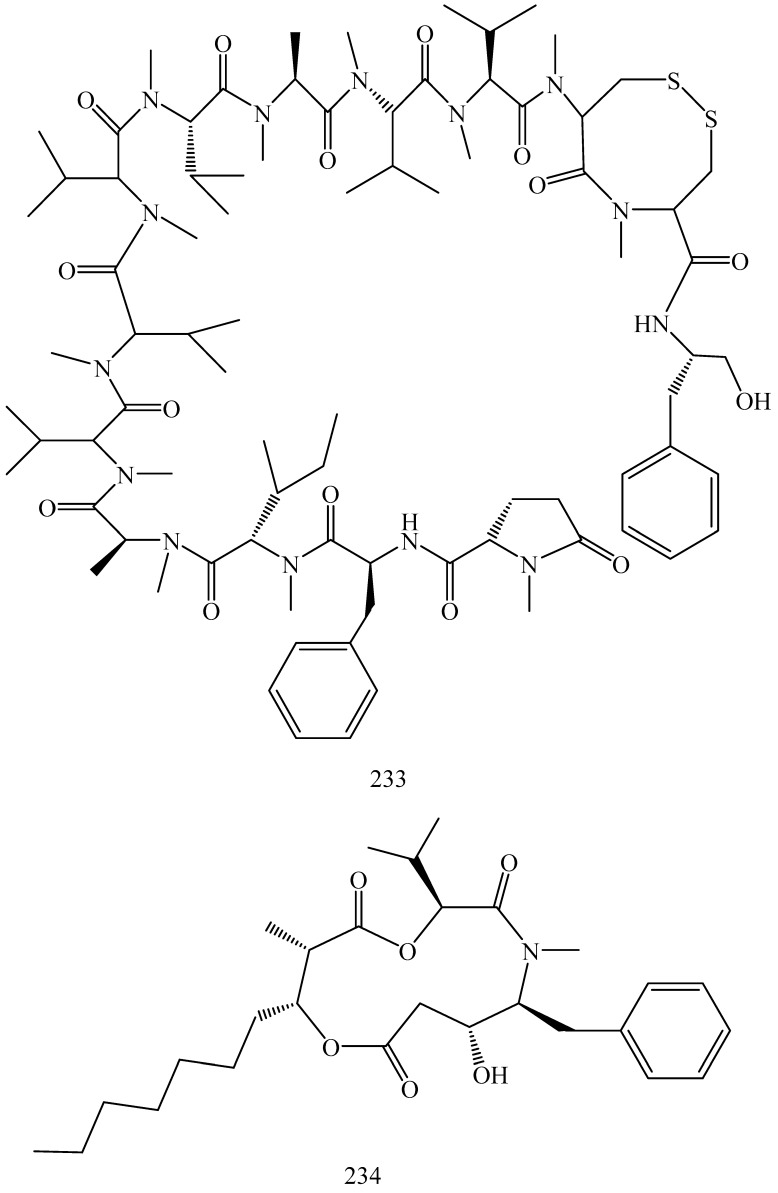

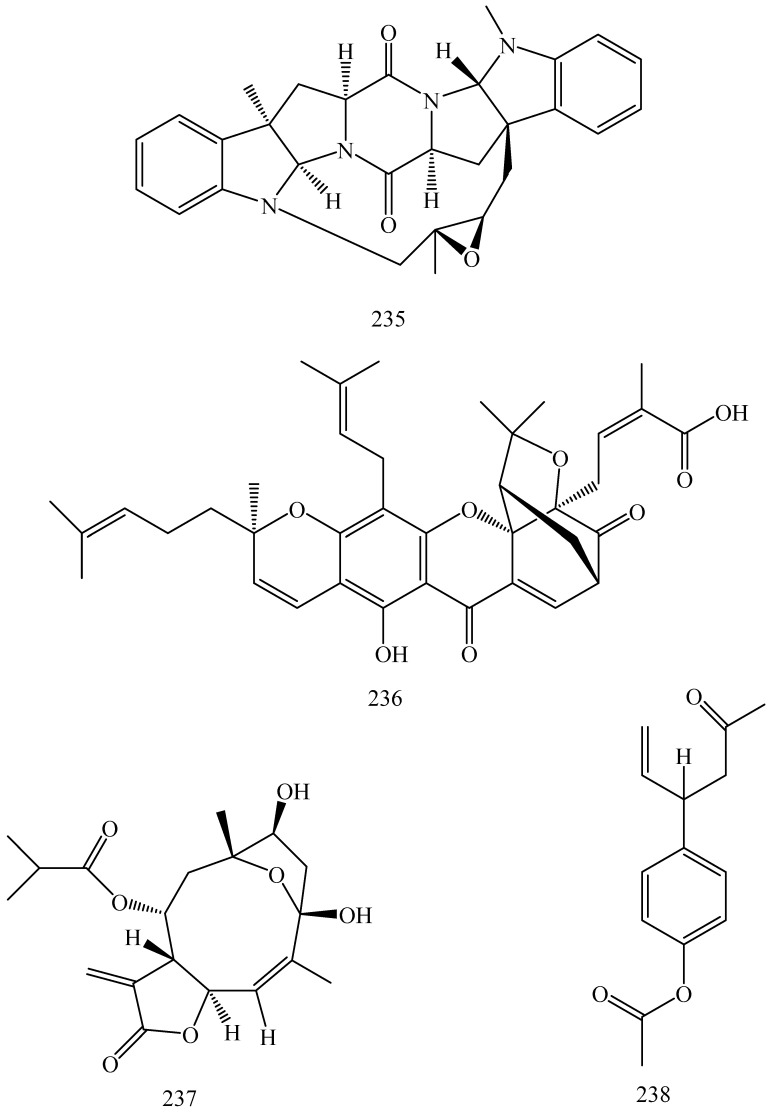

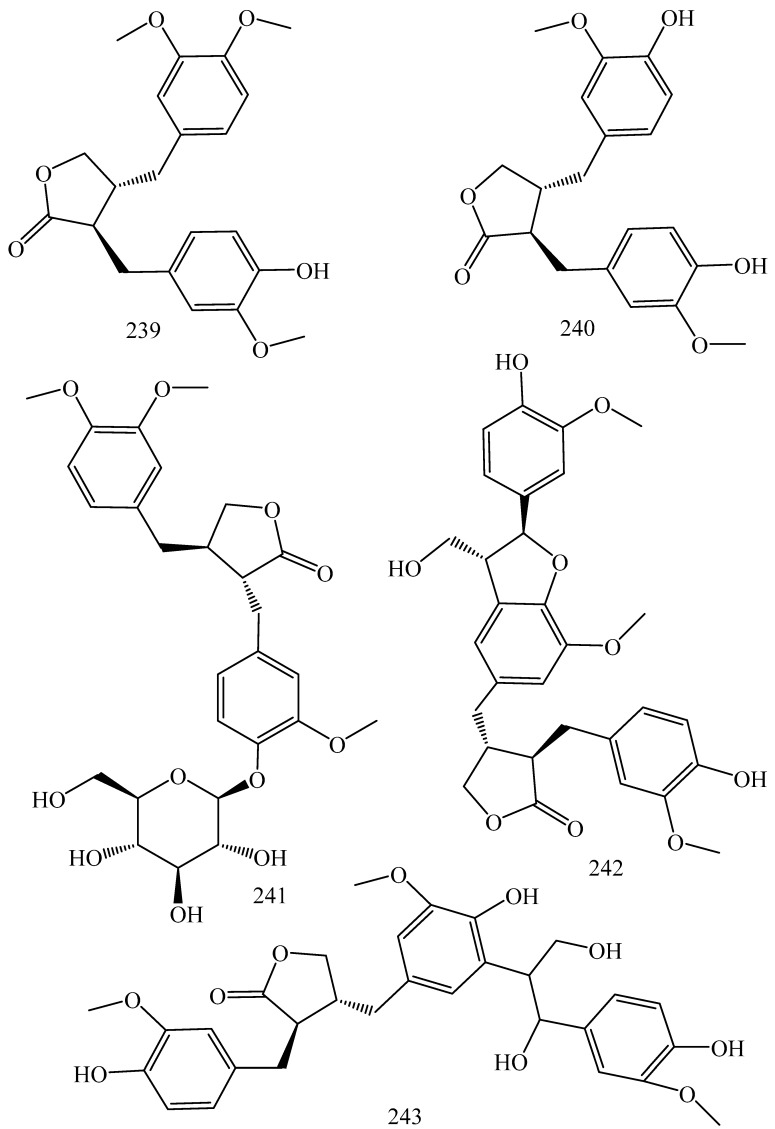

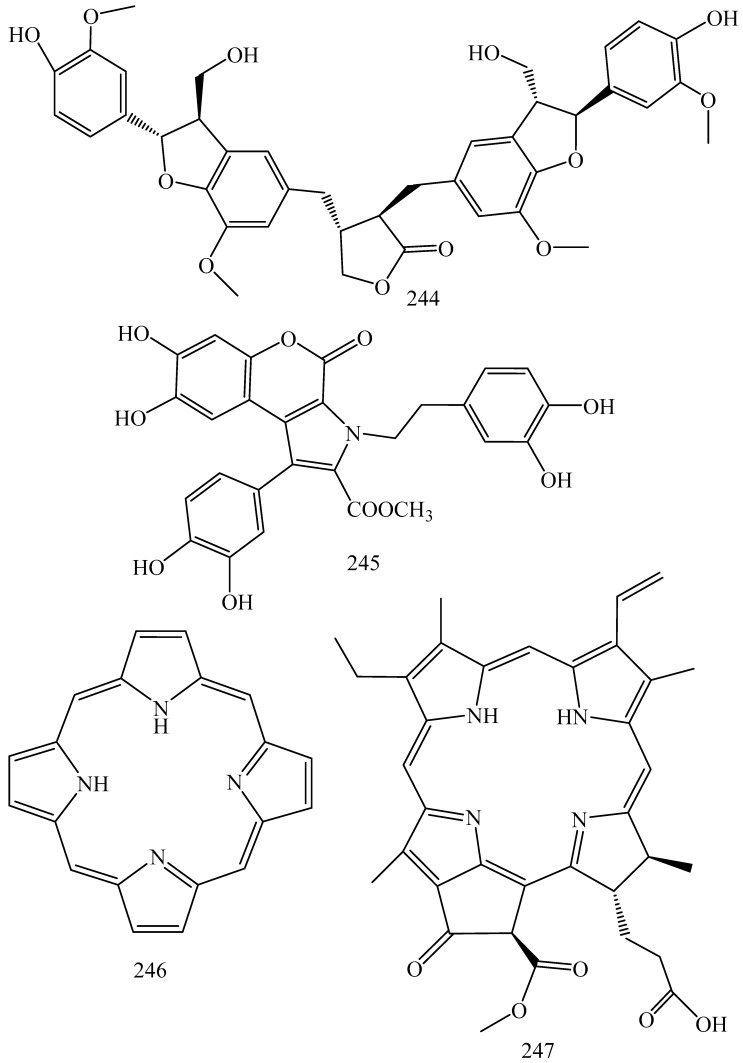

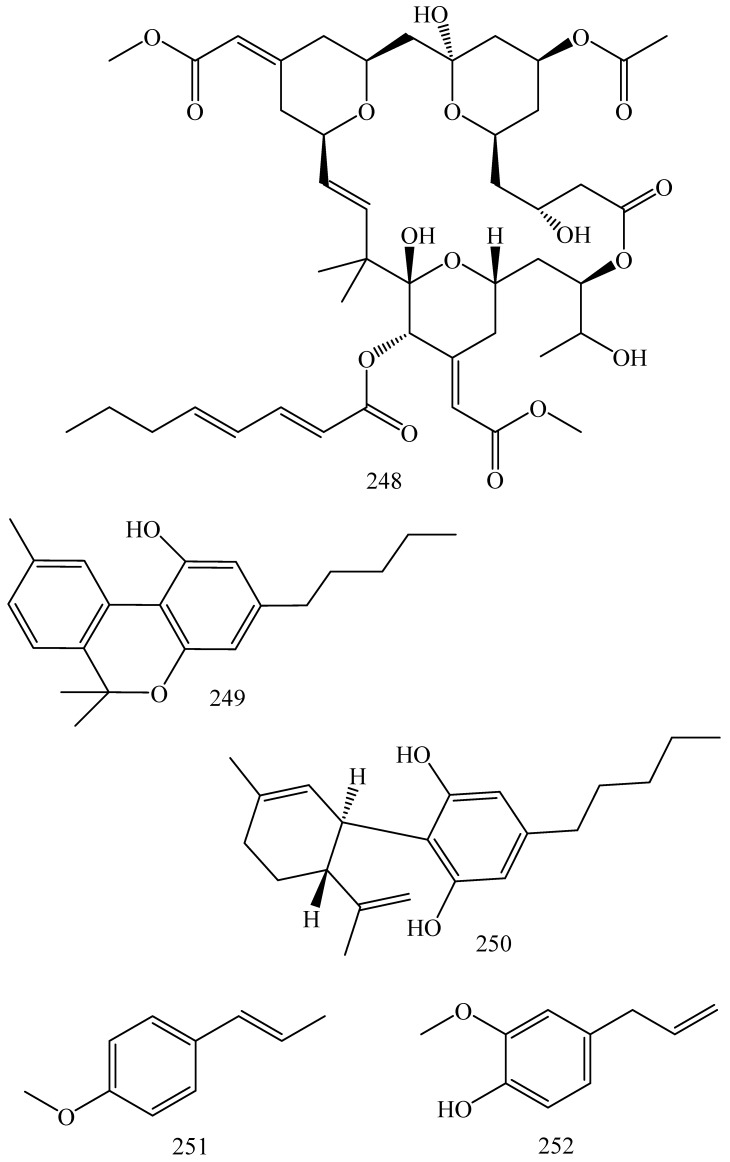

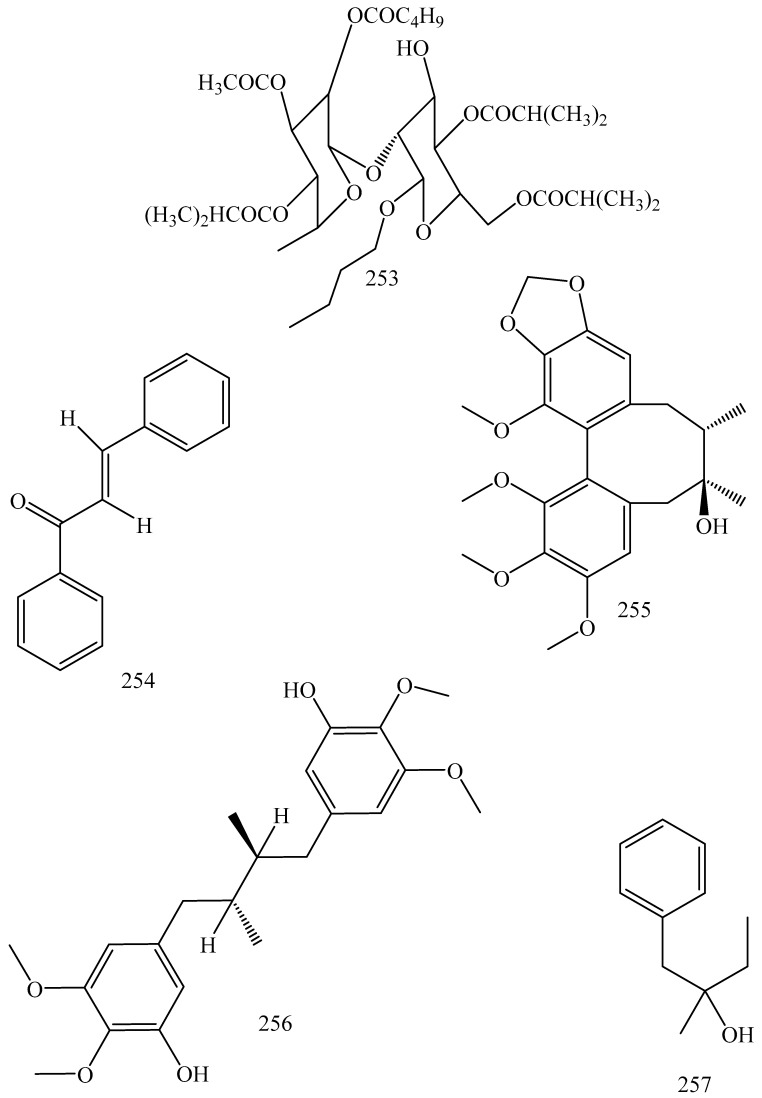
The structures of natural P-gp inhibitors mentioned in Table 3.

The inhibitors of P-gp are obtained from various natural sources in the form of alkaloids, flavonoids, coumarins, resins, saponins, terpenoids and miscellaneous other species [60]. Different P-gp inhibitors from natural sources are elaborately described in Table 3 along with the corresponding chemical structures (Figure 7).

#### 5.1.1. Alkaloids

Alkaloids are group of naturally occurring chemicals containing one or more basic nitrogen atoms. Existing literature have said that, many alkaloids have the ability to interact and prevent P-gp mediated drug efflux. The structural analysis of alkaloids proposed P-gp inhibitory activity due to presence of basic nitrogen atom/s and two planner aromatic rings. Alkaloids have been reported to inhibit P-gp via multiple mechanisms. Glaucine, an isoquinoline alkaloid, blocks P-gp and MRP1 dependent efflux and triggers ATPase action [75]. Later indicates that, it acts as a substrate of P-gp and can competitively inhibit P-gp [75]. Glaucine also helps in suppression of the expression of ABC transporter gene [75]. Pervilleine A, B and C, tropane alkaloids, are reported to exhibit P-gp inhibitory activity via inhibition of P-gp gene expression [80]. Berberine has been reported to act as a substrate for NorA pump and thereby exerting P-gp inhibitory effect in wild-type *Staphylococcus aureus* [69]. Kopsiflorine is known to inhibits mRNA expression of MDR1 gene and enhances the cytotoxic potential of vincristine in drug resistant KB [84,85]. Lobeline has been proven to be effective in inhibiting P-gp activity via substrate competition [90]. Literature revealed that, lobeline potentiates the gradual accumulation of doxorubicin in Caco-2 and CEM ADR5000 cells [90]. Literature revealed that, lobeline potentiates the gradual accumulation of doxorubicin in Caco-2 and CEM ADR5000 cells [90]. Cepharanthine, a bis-benzylisoquinoline alkaloid, reinstates the MDR activity in P-gp over-expressed KB-8-5 cells and enhances chemotherapeutic potential of vincristine [105]. Cepharanthine is predicted to inhibit the function of P-gp by directly interacting with the drug binding site of P-gp [115]. Ibogaine has been reported to inhibit P-gp activity via suppressing MDR1 and BCRP expressions in hMDR1-and hBCRP-transfected HEK293 cells and thereby enhances mitoxantrone accumulation [120]. Theobromine has been reported to inhibit AcrAB-TolC efflux pump, as a consequence the activity of ciprofloxacin is enhanced in some typical bacteria [121]. Steroidal and indole type alkaloids from *Veratrum* species, viz. deoxypeganine, verabenzoamine, veratroilzigadenine, veranigrine, 15-*O*-(2-methylbutyroyl)germine and veralosinine, have been reported to reduce MDR in human MDR1-gene-transfected mouse lymphoma cells (L5178Y) [122].

#### 5.1.2. Flavonoids and Phenolics

Flavonoids are a group of secondary metabolites found in a variety of fruits and vegetables. These are the polyphenolic molecules containing 15 carbon atoms and having a structure similar to that of flavone. Some flavonoids have been reported to possess significant P-gp inhibitory activity via diverse mechanisms. Morin, phloretin, phloridzin are reported to inhibit P-gp ATPase via binding to the ATP-binding site and thereby increase in the accumulation of daunomycin in P-gp overexpressing MCF-7/Adr cells [137]. Rhamnetin has been reported to inhibit Notch-1 signaling pathway and P-gp protein expression and enhances the performance of adriamycin, etoposide, paclitaxel and sorafenib in MDR hepatocellular carcinoma cells (HepG2/ADR) [164]. Plagiochin E is known to inhibit Cdr1p efflux pump and mRNA expression of CDR1 gene [165]. Daidzin stimulates ATPase activity coupled with inhibiting BCRP expression and as a result increases accumulation mitoxantrone and bodipy-FL-prazosin in mitoxantrone selected BCRP-overexpressing epithelial breast cancer cell line (MCF/MR) [135,166]. Procyanidine reverses P-gp associated MDR by inhibiting the function and expression of P-gp through down-regulation of NF-κB activity and MAPK/ERK pathway mediated YB-1 nuclear translocation in MDR human ovarian cancer cell line (A2780/T) [171,172]. Acacetin and robinin is known to stimulate ATPase activity and inhibits MRP1 expression in human erythrocyte [125]. Isorhamnetin has been reported to inhibit P-gp, MRP-2 and BCRP in Caco-2 cells [156]. It also inhibits bacterial TetK efflux pump in *Mycobacterium smegmatis* and thereby enhances the activity of isoniazid [174]. Rotenone, formononetin, afrormosin are reported to Inhibits P-gp via synergism with substrate [125]. Apigenin inhibits BCRP protein expression and thereby prevents mitoxantrone efflux in MCF-7 MS100 cells [138].

#### 5.1.3. Terpenoids

Terpenoids are derived from C_5_H_8_ isoprene units joined in a head to tail manner. They are classified as monoterpenoids (10 carbons), sesquiterpenoids (15 carbons), diterpenoids (20 carbons), and triterpenoids (30 carbons) [247]. Terpenoids have been reported to possess significant P-gp inhibitory activity via several mechanisms. Citral, a monoterpenoid, directly inhibits MRP1 and MRP2 via binding to their active sites in isolated Sf9-MRP1- and Sf9-MRP2-membrane vesicles [187]. Latilagascene B, latilagascene E and latilagascene D inhibit P-gp mediated MDR via directly blocking its active sites and thereby reverse doxorubicin resistance [190]. Paraliane, pepluanin A, jolkinol B, euphoportlandol A, euphoportlandol B, helioscopinolide A, helioscopinolide B, helioscopinolide E, helioscopinolide F, tuckeyanols A, tuckeyanols B and euphotuckeyanol are some of terpenoids, which inhibit P-gp activity via binding with its active sites [191,192,193]. Isopimaric acid inhibits microbial TetK or NorA efflux pumps and potentiates antibiotic activity in *Staphylococcus aureus* [202]. Totarol has been reported to inhibit NorA efflux pump and thereby enhances the effectiveness of antibiotics against *Staphylococcus aureus* [203].

#### 5.1.4. Saponins, Sapogenins and Sterols

Saponins are classified as steroidal and triterpenoidal. Sapogenins are free aglycones of saponins, which may be steroids, sterols, and triterpenoids. These exibit P-gp reversal activities via different mechanisms. Astragaloside II is reported to down-regulates the expression of the P-gp and MDR1 genes and thereby participates in 5-fluorouracil-resistance in human hepatic cancer cells, Bel-7402/FU [207]. Gracillin is known to inhibit P-gp mediated daunorubicin efflux in K567/R7 cells via direct interaction with active binding sites [208]. Tenacissimoside A has been reported to reverses MDR in P-gp overexpressing cancer cells (HepG2/Dox cells) toward doxorubicin, vinblastine, puromycin and paclitaxel via direct interaction with P-gp substrate site [210]. Karavilagenin C inhibits Rv1258c efflux pump and thereby augments antimicrobial activity of ethidium bromide to *Enterococcus faecalis* [211,212]. Balsaminol and balsaminagenin inhibits AcrAB-TolC efflux pump and potentiate actimicrobial activity in *Staphylococcus aureus* and *Escherichia coli* [211,212]. Pinnatasterone shows inhibition of P-gp-mediated daunorubicin efflux in K562/R7 cells via direct interaction with active binding sites [195]. Ginsenoside F_1_ inhibits P-gp ATPase activity and exhibits P-gp inhibitory activity on MDR1-MDCKII and Caco-2 cells [196]. Agosterol A inhibits ATP-dependent drug efflux by P-gp and MRP1 resulting reversal of colchicine resistance in KB-C2 cells [7]. Protopanaxatriol directly inhibits P-gp mediated substrate transport. Ginsenoside F_1_ inhibits P-gp ATPase activity and thereby inhibits P-gp in daunorubicin- and doxorubicin-resistant acute myelogenous leukemia sublines (AML-2/D100 and AML-2/DX100) [213]. 20(*S*)-Ginsenoside F_1_ inhibits P-gp ATPase activity and shows P-gp inhibitory activity on MDR1-MDCKII and Caco-2 cells [196].

#### 5.1.5. Coumarins

Various types of coumarins like furanocoumarins, pyranocoumarins, and sesquiterpenoid coumarins were investigated for their activity as P-gp inhibitors. Coumarins have been reported to inhibit P-gp through multiple mechanisms. Decursinol inhibits P-gp in Caco-2 cells via inhibition of efflux transporters like BCRP and MDP 2 [217]. GUT 70, a tricyclic coumarin, acts on P-gp overexpressing human leukemic cell lines by inhibiting the drug efflux mechanism [218]. Bergaptol inhibits vinblastine efflux from human MDR_1_ cDNA transfected LLC-GA5-COL300 cells via inhibition of MRP2 function [219]. Galbanic acid has been reported to inhibit P-gp via competitive binding with P-gp active sites and also inhibits NorA or NorB efflux pump [222]. Farnesiferol A, farnesiferol B, and farnesiferol C have been reported to inhibit P-gp active substrate binding sites and inhibit doxorubicin resistance in MCF7/Adr cells [221,222]. Cnidiadin enhances vinblastine or vincristine performance in MDCK-MDR1 and KB/VCR cells by acting as chemo-sensitiser for P-gp and inactivates it via blocking its efflux function [226].

#### 5.1.6. Peptides

There are some peptide compounds which act as P-gp inhibitors through different mechanisms. Peptides are the stimulators of protein kinase C (PKC) as well as cytotoxicity enhancer. Discodermolide reverses paclitaxal resistance in colon carcinoma (SW620AD-300) and ovarian carcinoma cell line (A2780AD) cells [227]. Kendarimide has been reported to reverse colchicin resistance in human carcinoma cell line (KB-C2) via direct inhibition of efflux mechanism [228]. Hapalosin reverses MDR in P-gp overexpressing, vinblastine-resistant human ovarian adenocarcinoma cells via direct inhibition of efflux mechanism [229]. Nocardioazine reverses MDR in SW620AD-300 cells via inhibition of membrane bound P-gp efflux protein [7,230].

#### 5.1.7. Resins

Some resins are also tested for their P-gp inhibitory activity. Gambogic acid is reported to enhance the cytotoxicity of two clinically popular anti-cancer drugs, docetaxel and adriamycin in MCF-7/Adm cells via inhibition of ABCB1 through its protein degradation by proteasome pathway [231]. Orizabin reverses norfloxacin resistance in *Staphylococcus aureus* via inhibition of NorA efflux pump [232].

#### 5.1.8. Miscellaneous Natural Compounds

There are some other natural compounds which show significant reversal of MDR activity like lignans, statins, cannabinoids etc. Acetoxy cavicolacetate inhibits NorA efflux pump and thereby potentiates the activity of ethidium bromide in *Staphylococcus aureus* [233]. Arctigenin, matairesinol, arctiin, isolappaol A and lappaol F potentiate doxorubicin mediated cytotoxicity in CaCo2 and CEM/ADR5000 cells [234]. Pheophorbide enhances the activity of ciprofloxacin in *Pseudomonas aeruginosa* through inhibition of MexAB-OprM efflux pump [236]. Porphyrin inhibits NorA efflux and reverses ciprofloxacin and norfloxacin resistance [236]. Cannabinol and cannabidiol have been reported to inhibit P-gp and BCRP mRNA expressions in MCF-7/p-gp cells and enhance cyclosporine A accumulation [238,239]. Polyacylated neohesperidosides and chalcone inhibits NorA Efflux pump and inhibit antibiotic resistance to the microorganisms [242,243]. Gomisin and Pregomisin, the lignans, shows MDR reversal phenomena on human HepG2 hepatoma cell lines through uncompetitive inhibition of P-gp-ATPase activity and alters P-gp substrate interactions [245]. Phenylbutanoid inhibits P-gp mediated MDR expression and promotess daunomycin uptake in breast cancer cells (MCF-7/Adr) [246].

### 5.2. Importance of P-gp Inhibitors in Various Therapies

P-gp shows MDR by affecting the absorption, distribution, excretion and metabolism of drugs that reduces the affectivity of certain drugs like anticancer, antibiotic, antidepressant, antihypertensives, antiarrythmics, calcium channel blockers, immunosuppressant, HIV protease inhibitors, and cardiac glycosides. P-gp mainly shows its effect in MDR in cancer in various human tumors by resisting apoptosis inducing by certain stimuli including TNF, Fas, serum starvation and UV irradiation [248].

In AIDs patients, P-gp expresses its resistance potential against protease inhibitors, such as indinavir, ritonavir, saquinavir, nelfinavir and it also shows MDR in some parasitic diseases which are caused by *Plasmodium falciparum* [249], *Entamoeba histolytica* [250], *Leshmania tropica* [251], etc. P-gp helps to efflux a wide range of xenobiotics that are taken along with nutrients at the apical membrane of secretory cells like adrenal gland, liver, kidney, placenta, and testes. P-gp hinders the accumulation of xenobiotics in the brain and pregnant uterus. P-gp removes xenobiotics through urine, bile and hormones. P-gp also prevents the absorption of molecules as it present in gastrointestinal tract cells after oral administration and also blocks in the brain the entry of antiviral drugs. P-gp inhibitors are used to treat various diseases like cancer, parasitic disease, HIV, epilepsy, and other disorders.

#### 5.2.1. P-gp Inhibitors in Cancer Chemotherapy

The overexpression of P-gp which pumps chemotherapeutic drugs outside the cell via ATP hydrolysis is the major mechanism of drug resistance. By this process, P-gp restricts the intracellular retention and cytotoxicity of chemotherapeutic agents and manifests a MDR phenotype to the tumor. Doxorubicin, a substrate of P-gp is widely used in malignancies. From analysis of the experimental data, we can see that two repeated low doses of doxorubicin induce an oxidative stress-mediated cytotoxicity in drug resistance cancer cells. The MDR1 (ABCB1) gene is present on chromosome 7q21 [4], which occurred by energy dependent transporter.

Natural compounds including flavonoids such as quercetin, epigallocatechin gallate, curcumin and capsaicin could reverse the MDR by inhibiting of efflux of P-gp. The signaling pathways that control NF-κB activation play vital role in controlling inflammation and oncogenesis. By Tumor Necrosis Factor (TNF), bacterial endotoxin, carcinogens activate NF-κB that causes oncogenesis. Phytochemicals obtained from dietary sources such as curcumin, capsaicin, guggulsterone, caffeic acid phenetyl ester (CAPE), anethol, eugenol helps to block the NF-κB activation process and acts as cancer chemopreventive agents. Natural NF-κB inhibitors like CAPE, licochalcone A, anacardic acid, enhanced the cellular buildup of daunorubicin or Rh 123 accumulation in κB/MDR1 cells. These compounds also stimulate ATPase activity of P-gp but lupeol, anethol, eugenol had no effect on the accumulation of daunorubicin, which are reported to suppress NF-κB activation [4]. Natural compounds are beneficial and used safely for increasing the effectiveness of cancer chemotherapy by inhibiting both of the NF-κB activation and anticancer drug efflux transporter. Natural compounds that inhibit NF-κB activation also have interactions with P-gp. The following naturally obtained drugs are used as anticarcinogenic treatments. Molecular modeling of Strychnos alkaloids docked to a homolog of P-gp was employed to optimize ligand protein interactions with increased affinity to P-gp. The compounds, which were evaluated by computational-based design, have more binding efficacy to P-gp and MDR reversal activity compared to verapamil [252]. P-gp inhibitors also show activity in the treatment of breast cancer. Breast cancer is among the most serious threats to women. For the treatment of breast cancer, chemotherapy and endocrine therapy is the predominant treatment approach. Breast cancer treatment may fail or relapse due to the progression of resistance against chemotherapeutic agents. The species which may confer resistance to cancer cells are the ABC transporters, such as P-gp, MRPs and BCRP, which dynamically distinguish and expel drugs from cancer cells. P-gp is the key factor that confers cancer (to apoptosis or programmed cell death) resistance, by attaching to the downstream caspase-3 and caspase-9. From a previous study, we came to know irinotecan, an anticancer agent, which is effective in patients with gastrointestinal malignancies. It is also used to treat unpredictable haematological intestinal or systemic toxicities [253]. Irinotecan detoxification involves the active drug efflux from cell through ABC transporters, like P-gp (ABC1 in human and abcb1a, abcb1b in mice) and MRP2 (Abcc2 in mice and ABCC2 in human) [254]. PSC833 (PSC), a second-generation P-gp inhibitor in vitro [255], in vivo [256] was used as a pharmacological activity of P-gp for irinotecan chronotherapy in female B6D2F_1_ mice [257].

#### 5.2.2. P-gp Inhibitors in the Treatment of HIV

All HIV protease inhibitors are transported via P-gp in the order ritonavir > nelfinavir > indinavir > saquinavir. From experimental studies, we can see that in a MDR-1 knockout mouse, plasma levels of indinavir, saquinavir and nelfinavir were 2–5 times higher compared with control mice. P-gp acts by limiting oral bioavailability and tissue distribution of protease inhibitors, with serious implications for the effectiveness of protease inhibitors. Inhibition of P-gp may be beneficial to facilitate greater intestinal absorption, bioavailability and penetration of protease inhibitors into HIV sanctuary sites as well as reduced excretion. Higher protease inhibitor levels in these sites may cause more suppression of viral replication. P-gp inhibitors like cyclosporine and verapamil inhibited the transport of HIV protease inhibitors.

#### 5.2.3. P-gp Inhibitors in Antimicrobial Therapy

P-gp transporters were identified in micro-organisms including bacteria, fungi, and protozoa [7]. P-gp is one of the most important transporters which is responsible for MDR in most micro-organisms. Many scientists have investigated the influence of P-gp inhibitors from natural sources (vasicine acetate, canthin-6-one, ergotamine, berberine, harmaline, reserpine, theobromine, chelerythrine, isorhamnetin, aegicerin, galbanic acid, orizabin, porphyrin, etc.) on the antimicrobial activity of antimicrobial agents that can increase their accumulation inside the cells and increase the antimicrobial action [61,62,69,72,94,121,124,174,186,224,232,236,258].

### 5.3. Challenges of Selecting Natural Molecules in Place of Existing P-gp Inhibitors

Nature has a wide variety of bioactive molecules and many of these serve as P-gp inhibitors. Natural molecules have structural diversity, which provide a valuable tool in the search of highly target specific P-gp inhibitors. It has been observed that many P-gp inhibitors from natural sources are very non-specific, but less toxic in nature. Therefore, due to their low toxicity level research on natural P-gp inhibitors is presently gaining interest. Challenges of using natural molecules in place of conventional synthetic molecules are stated as structural diversity, non-specific binding with the targets, unwanted pharmacokinetic changes may take place and extensive research is needed to establish the drug-like charecteristics of these molecules [259], but there are ceratain good aspects in using natural products like their variety of structures, less toxicity and the natural products would be helpful in designing and synthesizing new molecules with more selectivity towards P-gp transporters. Conventional P-gp inhibitors have some limitations and always produce toxic effects towards normal cells. However, reserch on newer synthetic molecules are going on and some of them are also certified for human use although there is still a lack of proper investigation regarding toxicitiy. In this aspect, the use of natural molecules is more advantageous due to their low toxicity and high efficacy towords the targets. There are some contradictory statements that increase the challenges of using natural products like quercetin, which reportedly stimulates P-gp mediated efflux and increases the resistance of anticancer drugs in MDR cells [260,261] while another study showed that quercetin inhibited P-gp and decreased the resistance of anticancer drugs [133], so it is necessary to evaluate all the natural molecules by some standerd methods and all research must also be more specific and focused to avoid such contradictions.

### 5.4. Toxicity Due to P-gp Inhibition by Phytochemicals

It is true that inhibition of efflux transporter is essential for the enhancement of the activity of the synthetic and natural compounds to reverse MDR. This is also true that non-specific inhibition may produce unwanted adverse effects on other essential cellular functions. Sometimes, inhibition of P-gp leads to excessive accumulation of cytotoxic drugs and poor excretion rates which in turn produce toxicity to the normal cellular function. Starting from first generation inhibitors, these have the ability to inhibit P-gp but possess high serum concentrations (at the doses that are required to inhibit P-gp) and produce potential toxicity [50,51]. Second generation inhibitors, which include cyclosporin A (valspoder or PSC833) and the *R*-isomer of verapamil (dexverapamil, without any cardiac activity), possess a greater P-gp affinity with no pharmacological effects, second generation inhibitors however have also failed to prove any significant toxicity reduction. These inhibitors inhibit the CYP3A4 enzyme and other ABC transporters and as a result the metabolism rate decreases leading to critical pharmacokinetic alterations. Third generation inhibitors are better than previous generations and these are more specific towards the targets, but problems of excessive drug accumulation are still there. Natural molecules are comparative newcomers in the field of P-gp inhibition with promising results, but there is still a toxicity issue regarding non-specificity for the targets and alteration of the pharmacokinetic parameters of substrates. Compounds like quercetin could competitively inhibit the members of MDR family, P-gp, MRP1 and BCRP [133,134,135,136], and also the metabolizing enzyme, CYP3A4 [262], therefore quercetin can alter pharmacokinetic parameters as well and produce toxicity. There are lots of discovered natural molecules and the activity of many towards ABC transporters has already been tested in different models. The possibilities of success with natural molecules are high, but more research is needed to identify better inhibitors with optimized activity from natural sources.

## 6. Conclusions and Future Prospective

It can be concluded that although MDR involves complex genetic factors, several modern scientific research lines could expedite the drug discovery process because each factor could provide a new target drug. Numerous research studies were carried out on MDR during the last three to four decades since Ling et al., discovered the role of an efflux transporter named P-gp in colchicine resistance in CHO cells [1]. This efflux transporter was found to play a pivotal role in drug pharmacokinetics and eventually interest started to accrue encompassing this transporter. In the earlier sections of this article, we have emphasized that P-gp is highly expressed in various tissues, and it is apparent that P-gp inhibition has great effects on drug pharmacokinetics. Most of the plant-based chemicals mentioned in this review could provide a vivid insight into a wide range of possibilities of using different techniques to improvise and develop effective P-gp inhibitors. Some of the plant-based compounds’ bioactives are reported to involve non-specific P-gp inhibition, and the process could affect other proteins and enzymes. Therefore, it is quite logical to seek to develop effective P-gp inhibitors which would be less toxic, highly specific and follow deffrent mechanisms of action. Some plant-based molecules are also active against microbial efflux systems and some are active in both humans and microbes, so there may be a probability those molecules that are active against microbial efflux systems may affect the efflux system in cancer cells. Further research is needed to prove this hypothesis and find new novel P-gp inhibitors. Finally, modern experimental methodologies and techniques, such as structure-activity relationships (SAR), quantitative structure-activity relationships (QSAR), 3-dimensional structure-activity relationships (3DQSAR), and pharmacophore studies should also be taken into consideration and should be regarded as an important guiding tool for the modern researchers in discovering very selective and potent P-gp inhibitors.

## Figures and Tables

**Table 1:** Examples of classical P-gp inhibitors by generation.

First Generation	Second Generation	Third Generation
Verapamil	(*R*)-Verapamil	Tariquidar (XR9576)
Cyclosporine A	Dexniguldipine	Zosuquidar (LY335979)
Vincristine	Elacridar (GF-120918)	Laniquidar (R101933)
Reserpine	Biricodar	ONT-093 (OC-144-093)
Quinidine	Dofequidar	Mitotane (NSC-38721)
Tamoxifen	Trifluoperazine	Annamycin
Trifluoperazine	Valspodar (PSC-833)	

**Table 2:** Mechanisms of classical P-gp inhibitors.

ATPase Activity		P-gp Expression		Competition for Binding Sites
Inhibitors	Stimulators	Down-Regulators	Up-Regulators	
Valspodar	Verapamil	Verapamil	Vincristine	Verapamil
Tariquidar	Cyclosporine A	Cyclosporine A		Cyclosporine A
Elacridar	Vincristine	Reserpine		Vincristine
ONT-093	Quinidine	Dexverapamil		Reserpine
	Tamoxifen	Toremifene		Quinidine
	Toremifene	Trifluoperazine		Valspodar
	Dexverapamil	Valspodar		Dexniguldipine
	Biricodar			Biricodar
				Elacridar
				Dofequidar

**Table 3:** Different drug efflux pump inhibitors from natural sources.

Sources	Compounds	Mechanisms of Action	Reported Literatures	Inhibitory Concentrations	Reference
Alkaloids					
*Adhatoda vasica* (Family: Acanthaceae)	Vasicine acetate (**1**), 2-acetyl benzylamine (**2**)	Not found.	Inhibits MDR strain of *Mycobacterium tuberculosis*.	Not found.	[61]
*Allium neapolitanum* (Family: Liliaceae)	Canthin-6-one (**3**), 8-hydroxy-canthin-6-one (**4**)	Inhibition of norA gene encoding the NorA MDR efflux protein, TetK tetracycline efflux protein and mecA gene.	Active against a panel of fast growing *Mycobacterium* species and MDR and MRSA strains of *Staphylococcus aureus*.	MIC ranges for *Mycobacterium* and *Staphylococcus aureus* are 8–32 and 8–64 µg/mL, respectively.	[62]
	5(Zeta)-hydroxy-octadeca-6(*E*)-8(*Z*)-dienoic acid (**5**)	Not found.	Active against MDR and MRSA strains of *Staphylococcus aureus*.	MIC range of 16–32 µg/mL.	
*Antizoma miersiana* (Family: Menispermaceae)	Cycleanin (**6**), Insularine (**7**), Insulanoline (**8**)	Inhibition of MDR activity is due to favorable structure activity relationship of these compounds (like presence of-OH group) which provide better solubility and attachment with target proteins.	All three compounds increase intracellular doxorubicin accumulation in MCF-7/Adr cell via reversal of MDR.	10 µM of all three inhibitors produces IC_50_ values for doxorubicin are 0.40, 0.38, 0.65 µM, respectively.	[63]
*Aspergillus fischeri* (Family: Trichocomaceae)	5-*N*-acetylardeemin (**9**)	Inhibition of MDR-[P-gp^+^ and MDR-associated protein (MRP)^+^], MDR-P-gp^+^, lung resistance protein (LRP)^+^-expressions.	Reverses resistance to doxorubicin in lung cancer (NSCLC) cells SW2R160 (MDR^+^) and SW2R120 (LRP^+^). It also reverses vinblastine and taxol resistance to CCRF-CEM/VBL_100_ cell lines via P-gp inhibition.	IC_50_ value for vinblastine and taxol are reduced to 0.00011 and 0.0018 μM, respectively in presence of 5-*N*-acetylardeemin.	[64]
*Aspergillus sydowii, Aspergillus fumigates* (Family: Trichocomaceae)	Fumitremorgin C (**10**)	Inhibits BCRP via competitive manner. This molecule has a planar, multi-ring structure like mitoxantrone and doxorubicin and therefore may compete with other cytotoxic drugs for the binding sites on the transporter.	It almost completely reverses resistance mediated by BCRP in MCF-7 cells transfected with this protein.	Not found.	[65]
*Camptotheca acuminate* (Family: Nyssaceae)	Camptothecin (**11**)	Not found.	Shows activity against P-gp on mouse L1210 leukemia cells.	Not found.	[66,67]
*Capsicum frutescens* (Family: Solanaceae)	Capsaicin (**12**)	Inhibits mRNA expressions of MDR1 and MRP1.	Increases the amount of Rh 123 accumulation in vinblastine-resistant colon carcinoma LS-180 cells via P-gp inhibition.	Not found.	[68]
*Catharanthus roseus* (Family: Apocynaceae)	Vincristine (**13**)	Inhibits P-gp function in BBB.	Acts as a P-gp reversal agent in the BBB tested using Rh 123 uptake in cultured bovine brain capillary endothelial cells (BCEC).	Not found.	[69]
*Cinchona pubescens* (Family: Rubiaceae)	Cinchonine (**14**), Hydrocinchonine (**15**), Quinidine (**16**)	Inhibits mRNA expression of P-gp.	Hydrocinchonine, cinchonine, and quinidine significantly increased the cytotoxicity of paclitaxol in P-gp-positive MES-SA/DX5. Cinchonine potentiated anticancer drug accumulation in vivo in phase I trials.	Not found.	[70,71]
*Claviceps purpurea* (Family: Clavicipitaceae)	Ergotamine (**17**), Ergometrine (**18**)	Directly inactivate P-gp function via acting as P-gp substrates and inhibit MDR1 and mdr1a expressions.	Inhibit human MDR1 and the mouse ortholog MDR1a. Ergotamine inhibited the NorA efflux pump of *Staphylococcus aureus* and potentiated the activity of norfloxacin on it.	Not found.	[72,73]
*Coptis japonica* (Family: Ranunculaceae)	8-Oxocoptisine (**19**)	Not found.	Shows P-gp mediated MDR reversal activity in MES-SA/DX5 and HCT15 cells and enhances cytotoxicity of paclitaxel.	ED_50_ values of paclitaxel are reduced to 0.018 and 0.0005 µg/mL in MES-SA/DX5 and HCT15 cell lines, respectively.	[74]
*Corydalis yanhusuo, Corydalis turtschaninovii* (Family: Papaveraceae)	Glaucine (**20**)	Inhibits P-gp and MRP1-mediated efflux and activates ATPase activities of the transporters. So, acts as a substrate and inhibits P-gp and MRP1 competitively. Suppresses the activity of ABC transporter gene.	Inhibits MRP1 and P-gp mediated efflux tested in human breast cancer cells, MCF-7.	Not found.	[75]
		Inhibits MMP-9 gene expression through the suppression of NF-κB.	Directly inhibits the migration and invasion of human breast cancer cells.	15 and 30 μM inhibited 48% and 63% of cell viabilities, respectively.	[76]
*Cynanchum paniculatum* (Family: Apocynaceae)	(−)-Antofine (**21**)	Down-regulates of P-gp mRNA and protein expressions.	Increases intracellular Rh 123 accumulation in paclitaxel resistant human lung cancer cells (A549-PA).	Not found.	[77]
*Ecteinascidia turbinata* (Family: Perophoridae)	Trabectedin (ET-743) (**22**)	Down-regulates MDR1 gene expression. Inhibits P-gp gene expression.	Shows good anti-cancer activity in vitro against mouse lymphocytic leukemia (L1210) cells. Inhibits P-gp expression in overian cancer and epidermal carcinoma (KB-C2 and KB-8-5, respectively).	0.5 ng/mL. Not found.	[78,79]
*Erythroxylum pervillei* (Family: Erythroxylaceae)	Pervilleine A (**23**)	Inhibits P-gp gene expression.	Restores the vinblastine sensitivity of cultured multidrug resistant KB-VI cells through P-gp inhibition.	0.36 µM.	[80]
			Vinblastine sensitivity is also restored on CEM/VLB_100_ cells.	0.02 µM.	[80]
			Chemosensitivity of KB-8-5 cells to colchicine is restored by pervilleine A.	0.61 µM.	[80]
	Pervilleine B (**24**), Pervilleine C (**25**)	Inhibit of P-gp gene expression.	Both of these are found to restore the vinblastine sensitivity of cultured MDR KB-VI cells.	0.17 µM for each compound.	[80]
*Hepalosiphon welwitschii* (Family: Hepalosiphonaceae)	*N*-methyl welwitindolinon C-isothiocyanate (**26**)	Not found.	Enhances the cytotoxicity of actinomycin D and daunomycin in vinblastine-resistant ovarian carcinoma (SK-VLB-1) cells. Increases the activity of vinblastine, taxol, actinomycin D, colchicine and daunomycin in breast carcinoma (MCF-7/ADR) cells.	Not found.	[81]
*Hydrastis canadensis* (Family: Ranunculaceae)	Berberine (**27**)	Not found. Berberine acts as a substrate for NorA pump.	Increases Rh 123 accumulation in cultured bovine brain capillary endothelial cells (BCEC) via inhibition of P-gp. Berberine inhibits NorA pump (MDR pump) in wild-type *Staphylococcus aureus* RN 4222.	Not found.	[69,82]
*Ipomoea muricata* (Family: Convolvulaceae)	Lysergol (**28**)	ATPase inhibition and down-regulation of MDR ABC transporter ATP-binding yojI gene.	Inhibits the ABC pump YojI of *E. coli* (MTCC1652 and KG4).	Not found.	[83]
*Kopsia dasyrachis* (Family: Apocynaceae)	Kopsiflorine (**29**)	Inhibits mRNA expression of MDR1 gene.	Enhances cytotoxicity of vincristine in MDR KB cells.	2.3 µg/mL.	[84,85]
*Lamellaria* spp. (Family: Velutinidae)	Lamellarin I (**30**)	Directly binds with active drug binding sites of P-gp and reverses its function.	Increases the intracellular concentration of Rh 123 in human colon adeno carcinoma cell line (Lo Vo/Dx). 2 µM lamellarin I has MI (fold decrease in resistance/modulator µM concentration) values of 53, 99 and 105 for doxorubicin, daunorubicin and vinblastine in MDR P388/Schabel cells, respectively. These values are 9 to 16 folds > than those obtained with 2 µM of verapamil.	Mentioned in previous column.	[86]
*Lissoclinum patella* (Family: Didemnidae)	Patellamide D (**31**)	Not found. Acts as a selective antagonist in multidrug resistance.	Directly acts as cytotoxic agent and acts against L1210 murine leukemia cells. Reverses the MDR in the human leukemic cells (CEM/VLB100). Patellamide D at 3.3 µM was compared with 5.1 µM verapamil in modulating drug resistance in vitro.	2–4 µg/mL. IC_50_ for vinblastine, colchicine and adriamycin was reduced from 100 to 1.5 ng/mL, 140 to 50–100 ng/mL and 1000 ng/mL to 110 ng/mL, respectively.	[87,88,89]
*Lobelia inflata* (Family: Campanulaceae)	Lobeline (**32**)	Inhibits P-gp function probably by substrate competition.	Inhibits P-gp activity by sensitize resistant tumor cells at nontoxic concentration. Tested on Caco-2 cells. Also in CEM ADR5000 cells.	168.3 ± 23.68 µM. 219.3 ± 5.59 µM.	[90]
Marine actinomycetes	Arcyriaflavin (**33**), Staurosporin aglycone (**34**)	Directly interact with BCRP and ABCG2 proteins.	Show the most potent effect of BCRP inhibition in the BCRP-transferred HEK-293 cell line, with low toxicity in BCRP-transfected cells, and reduce the relative resistance of ABCG2-transfected cells.	Not found.	[91,92]
*Mirabilis jalapa* (Family: Nyctaginaceae)	*N*-trans-feruloyl 4′-*O*-methyldopamine (**35**)	Inhibits NorA efflux protein and works as a substrate.	Shows efflux transporter inhibitory activity in MDR *Staphylococcus aureus* overexpressing the multidrug efflux transporter NorA, and causes an 8-fold reduction of norfloxacin MIC.	Not found.	[93]
*Peganum harmala* (Family: Nitrariaceae)	Harmaline (**36**)	Inhibits NorA efflux pump.	Inhibits NorA pump in *Staphylococcus aureus* and enhances the activity of antibacterial agent, Ethidium bromide.	Not found.	[94]
	Harmine (**37**)	Inhibits BCRP via acting as substrate.	Removes resistance to the anticancer drugs mitoxantrone and camptothecin.	Not found.	[95,96]
		Decreases mRNA levels of the MDR1 gene.	Shows P-gp reversal activity on Caco-2 and CEM/ADR5000 cells using Rh 123 and calcein as P-gp substrate.	Not found.	
*Peschiera laeta*, *Peschiera fuchsiaefolia* (Family: Apocynaceae)	Coronaridine (**38**), Conoduramine (**39**), Voacamine (**40**)	Inhibits ATP dependent P-gp binding of substrates.	Enhances vinblastine accumulation and cytotoxicity in MDR KB cells.	EC_50_ value for vinblastine is reduced to 1.9, 0.6, 2.0 µM.	[97,98,99]
*Phellodendron amurense* (Family: Rutaceae)	γ-fagarine (**41**)	Inhibits P-gp mediated MDR.	Both of the compounds show MDR reversal activity in P-gp expressed MDR cells, MES-SA/DX5 and HCT15 and enhances cytotoxicity of paclitaxel.	ED_50_ values of paclitaxel with *γ*-fagarine in MES-SA/DX5 and HCT15 cells are 0.264 and 0.0100 µM, respectively.	[100]
	4-methoxy-*N*-methyl-2-quinolone (**42**)			ED_50_ values of paclitaxel with 4-methoxy-*N*-methyl-2-quinolone ED_50_ values of paclitaxel are 0.335 and 0.0170 µM, respectively.	
*Piper nigrum* (Family: Piperaceae)	Piperine (**43**)	In low dose, it inhibits P-gp expression and function but higher dose can enhance P-gp protein and MDR1 mRNA levels.	Inhibits and modulates P-gp function in dose dependent manner. In low dose, it inhibits p-gp and enhances digoxin potential but in higher dose it enhances p-gp function and reduces digoxin uptake observed in Caco-2 cell line. Piperine enhances anti-microbial activity of rifampicin in *Mycobacterium tuberculosis* H37Rv and rifampicin resistant *Mycobacterium tuberculosis* via inhibition of clinically over expressed mycobacterial putative efflux protein (Rv1258c).	Not found.	[101,102]
*Prosopis juliflora* (Family: Fabaceae)	Julifloridine (**44**), Juliflorine (**45**), Juliprosine (**46**)	Inhibits NorA efflux pump by directly inhibiting its function.	Inhibits NorA efflux pump of *Staphylococcus aureus* and potentiated norfloxacin activity.	Not found.	[103]
*Rauwolfia serpentina* (Family: Apocynaceae)	Reserpine (**47**)	Not found. Directly binds with NorA MDR pump and inactivates it.	Produces significant enhancement of doxorubicin sensitivity in CEM-ADR5000 and KB cell cell line via P-gp inhibition.	13.2 ± 1.02 µM and 10 ± 3 µM, respectively.	[104,105,106]
			Active agaist ABC transporter pump (NorA MDR pump) in methicillin-resistant *Staphylococcus aureus* (MRSA) strains and enhances tetracycline sensitivity.	Not found.	
*Ruta graveolens* (Family: Rutaceae)	Arborinine (**48**), Evoxanthine (**49**), Gravacridonediol (**50**)	Reduces mRNA level of P-gp.	Induces intracellular Rh 123 accumulation in L5178 MDR cells via MDR reversal activity at 40 µM concentration. Provides synergistic activity with doxorubicine in L5178 MDR. Arborinine and evoxanthine also provide MDR reversal activity in human MDR1 gene-transfected mouse lymphoma cells at 400 μM.	IC_50_ for gravacridonediol + doxorubicine is 33.97 µM	[107]
*Sanguinaria canadensis* (Family: Papaveraceae)	Sanguinarine (**51**)	Acts via bimodal cell death mechanism or overcome the phenomenon of P-gp-mediated MDR by inducing apoptosis through increasing the Bax/Bcl 2 ratio and activating caspase 3.	Sanguinarine shows P-gp reversal activity on Caco-2 and CEM/ADR5000 cells using Rh 123 and calcein as P-gp substrate. Reversal of P-gp mediated MDR.	Not found.	[108,109]
*Sinomenium acutum* (Family: Menispermaceae)	Dauriporphine (**52**)	Inhibits P-gp mediated MDR.	Inhibits P-gp mediated MDR in MES-SA/DX5 and HCT15 cells and enhances cytotoxicity of paclitaxel.	ED_50_ values with paclitaxel are reduced to 0.03 and 0.00010 µg/mL in MES-SA/DX5 and HCT15 cells, respectively.	[110]
*Solanum lycopersicum* (Family: Solanaceae)	Tomatidine (**53**)	Inhibits P-gp mediated drug transport via direct binding with efflux pump.	Increase uptake of tetramethylrosamine in MDR-NCI Adr R human adenocarcinoma cells via inhibition of P-gp mediated drug transport and MDR.	Not found.	[111]
*Sophora alopecuroides* (Family: Fabaceae)	Matrine (**54**)	Not found.	Enhances the cytotoxicity of vincristine in resistant K562/VCR cells. Increases the intracellular accumulation of doxorubicin in resistant K562/DOX cell line.	Not found.	[112,113]
*Stemona aphylla*, *Stemona burkillii* (Family: Stemonaceae)	Stemocurtisine (**55**), Oxystemokerrine (**56**), Stemofoline (**57**)	Not found.	Act as P-gp reversing agent in KB-V1 cells at a concentration of 50 µM and increase the sensitivity toward the cytotoxic drug vinblastine.	Not found.	[114]
			Act as P-gp reversing agent in KB-V1 cells at the concentrations of 1, 3 and 5 µM and increase sensitivity to cytotoxic drugs vinblastine, paclitaxel and doxorubicin.	Not found	
*Stephania cepharantha* (Family: Menispermacea)	Cepharanthine (**58**)	Inhibits the function of P-gp by directly interacting with the drug binding site of P-gp	Acts on human KB carcinoma cells.	6.7 ± 4.3 µM.	[115]
*Stephania tetrandra* (Family: Menispermaceae)	Tetrandrine (**59**), Fangchinoline (**60**)	Reversal of P-gp-mediated MDR via direct inhibition of P-gp function.	Enhances anticancer activity of daunorubicin, etoposide and cytarabine in acute myeloid leukemia patients via P-gp inhibition. Produce reversal activity on P-gp mediated resistance to paclitaxel in vitro and in vivo in human MDR tumor cell line (KBv200) and resistant KBv200 tumors. Fangchinoline reduces resistance to paclitaxel and actinomycin D in HCT15 cells via MDR-reversal activity. Both the compounds increase the intracellular accumulation of the fluorescent P-gp substrate Rh 123 and inhibited its efflux in Caco-2 and CEM/ADR5000 cells.	Not found.	[116,117,118]
*Tabernanthe iboga* (Family: Apocynaceae)	Heyneanine (**61**), 19-epi-Heyneanine (**62**), Dippinine B (**63**), Dippinine C (**64**)	Not found.	All of these compounds show P-gp mediated MDR reversal activity in vincristine-resistant KB cells (KB/VJ300) in presence of vincristine 0.1 µg/mL.	IC_50_ values of vincristine in the presence of heyneanine, 19-epi-heyneanine, dippinine B and dippinine C (0.1 µg/mL) are 8.5, 3.5, 2 and 4 µg/mL, respectively.	[119]
	Ibogaine (**65**)	It significantly inhibits P-gp activity via suppressing MDR1 and BCRP expressions.	In hMDR1- and hBCRP-transfected HEK293 cells, it enhances mitoxantrone accumulation.	Not found.	[120]
*Theobroma cacao* (Family: Sterculiaceae)	Theobromine (**66**)	Inhibits AcrAB-TolC efflux pump.	Enhances the activity of ciprofloxacin on *Enterobacter cloacae*, *Klebsiella pneumonia, Salmonella typhimurium.*	Not found.	[121]
*Veratrum lobelianum* Bernh. (Family: Liliaceae)	Deoxypeganine (**67**)	Not found.	Reduces MDR in human MDR1-gene-transfected mouse lymphoma cells (L5178Y).	20.76 µg/mL.	[122]
*Veratrum nigrum* (Family: Liliaceae)	Verabenzoamine (**68**), Veratroilzigadenine (**69**), 15-*O*-(2-Methyl butyroyl)germine (**70**), Veralosinine (**71**), Veranigrine (**72**)	Not found.	Reduce MDR in human MDR1-gene-transfected mouse lymphoma cells (L5178Y).	21.76, 26.07, 24.86, 22.69 µg/mL, respectively.	[122]
*Zanthoxylum capense* (Family: Rutaceae)	Oxychelerythrine (**73**), Oxynitidine (**74**)	Inhibit bacterial NorA MDR pump.	Enhance activity of antibacterial agents like erythromycin, ethidium bromide, tetracycline and oxacillin in *Staphylococcus aureus*.	Not found.	[123]
*Zanthoxylum clava-herculis* (Family: Rutaceae)	Chelerythrine (**75**)	Not found.	Reverses the MDR in mdr-MRSA stain of *Staphylococcus aureus* via inhibiting the efflux mechanism.	Not found.	[124]
**Flavonoids and Phenolics**					
*Amorpha fruticosa* (Family: Fabaceae)	Amorphigenin (**76**)	Inhibits P-gp via synergism with substrate.	Potentiates the activity of epirubicin in human MDR1 gene-transfected mouse lymphoma cells.	Not found.	[125]
*Ampelopsis* spp. (Family: Vitaceae)	Ampelopsin (**77**)	Not found.	It reverses the MDR to adriamycin in K562/ADR cells.	Not found.	[126]
*Artemisia absinthium* (Family: Asteraceae)	4′,5′-*O*-dicaffeoyl quinic acid (**78**), 3′,5′-*O*-dicaffeoyl quinic acid (**79**)	Inhibits NorA efflux pump.	Enhances antimicrobial activity of berberine in resistant strains of *Staphylococcus aureus and Enterococcus faecalis.*	MIC of berberine + 4′,5′-*o*-dicaffeoyl quinic acid is 64 µg/mL.	[127]
		Not found	Enhances the activity of antimicrobial effects against resistant strains of microorganisms.	Not found	
*Artemisia annua* (Famiy: Asteraceae)	Chrysosplenol D (**80**), Chrysoplenetin (**81**)	Inhibit NorA MDR pump and plasmodial efflux pump.	Potentiate berberine and norfloxacin activity against resistant strains of *Staphylococcus aureus.* Also potentiate antimicrobial activity of artemisinin against *Plasmodium falciparum*.	Not found.	[128,129]
*Camellia sinensis* (Family: Theaceae)	Epigallocatechin gallate (**82**), Epicatechin gallate (**83**), Catechin gallate (**84**), Epicatechin (**85**)	Not found. Epigallocatechin gallate inhibits TetK efflux pump and potentiate the activity of isoniazide.	All of these compounds inhibit Rh 123 transport in CH^R^C5 cells via P-gp efflux inhibition. Epigallocatechin gallate is more effective than others. It is also effective against P-gp mediated transport of vinblastine in Caco-2 cells.	Not found.	[130,131,132]
			Epigallocatechin gallate inhibits P-gp mediated digoxin transport in Caco-2 cell line and it also inhibited metformin uptake in human embryonic kidney 293 (HEK) cells via inhibiting OATP1B1 (HEK-OATP1B1), OATP1B3 (HEK-OATP1B3), OCT1 (HEK-OCT1), OCT2 (HEK-OCT2), and MATE1 (HEK--MATE1) uptake transporters.	Not found.	
			Inhibits TetK efflux pump in *Mycobacterium intracellulare, Mycobacterium smegmatis, Mycobacterium xenopei,* and *Mycobacterium chelonei* in a combination with isoniazide	Not found.	
	Quercetin (**86**)	Inhibits transport of talinolol via replacing it from P-gp substrate binding sites. Quercetin could competitively inhibit the members of MDR family, P-gp, MRP1 and BCRP and the metabolizing enzyme, CYP3A4. Inhibits efflux function via non-competitive binding with P-gp and MRP1.	Inhibits talinolol transport in Caco-2 cell line via P-gp inhibition.	Observed IC_50_ values are 97 and 41 µM of talinolol in absence and presence of quercetin.	[133,134,135,136]
			Quercetin non-competitively inhibits the function of P-gp in K562/adr and MRP1 in GLC4/adr cells and increases pirarubicin’s cytotoxicity.	IC_50_ value s of pirarubicin are reduced to 23.0 ± 3.0 and 18.0 ± 8.5 µM, respectively in these two cell lines.	
*Cicer pinnatifidum* (Family: Fabaceae)	Biochanin A (**87**)	Modulates P-gp by interacting bi-functionally with the vicinal ATP-binding site and the steroid binding sites as well as inhibition of P-gp ATPase by binding to the ATP-binding site.	Potentiates cytotoxicity of doxorubicine in P-gp positive MDA435/LCC6MDR1 cells. Inhibits the activity of P-gp in recombinant human P-gp membrane	IC_50_ value of doxorubicin in MDA435/LCC6MDR1 cell line is reduced to 0.80 ± 0.20 µM in presence of Biochanin A.	[137,138,139]
		Inhibits BCRP protein expression.	In combination with mitoxantrone shows significant potentiation of cytotoxicity in MCF-7 MS100 cells.	Not found.	
		Inhibits TetK efflux pump.	Potentiates antimicrobial activity of ethidium bromide on *Mycobacterium smegmatis*.	Not found.	
*Citrus aurantium* (Family: Rutaceae)	3,3′,4′,5,6,7,8-heptamethoxy flavones (**88**), Tangeratin (**89**), Nobiletin (**90**)	Not found.	Enhance transport across Caco-2 cell monolayer via P-gp inhibition.	Not found.	[140]
*Citrus reticulata* (Family:Rutaceae)	5,6,7,3′,4′-pentamethoxy flavones (Sinensetin) (**91**)	Reverses P-gp mediated MDR.	Enhances cytotoxicity of vincristine in P-gp over expressing AML-2/D100 cells.	IC_50_ value for vincristine is reduced to 1.14 µM.	[141]
*Citrus paradisi* (Family: Rutaceae)	Kaempferol (**92**), Kaempheride (**93**), Naringenin (**94**)	Kaempferol and Naringenin both inhibit MDR-1 mRNA expression.	Kaempferol and Naringenin both decrease P-gp levels in the human immortalized proximal tubular cells (HK-2).	Not found.	[142,143,144,145,146]
			Naringenin enhances bioavailability of felodipine in whister rats via P-gp inhibition.	Not found.	
			Kaempferol and kaempheride both show P-gp inhibition in K562/BCRP cells.	Not found	
			Kaempferol decreases resistance of vinblastine and doxorubicin in vinblastine resistant KB-VI cell lines.	Kaempferol reduces IC_50_ values for vinblastine and doxorubicin to 1233 ± 202 and 47533 ± 2145 nM, respectively.	
			Naringenin is a potent inhibitor of P-gp observed via talinolol transport across Caco-2 cell monolayer.	Naringenin reduces IC_50_ values of talinolol to 236 _0_ values of mitoxan µM.	
			Kampherol in combination with mitoxantrone in mitoxantrone specific MDR MCF-7 MS100 cell line shows significant inhibition of P-gp function.	IC_50_ value of mixtrantrone (alone) is 199 ± 19.3 µM, while in combination with kampherol IC_50_ value is reduced to 3.36 ± 1.84 µM.	
			Naringenin shows P-gp inhibition in MDR MCF-7 MS100 cell line inhibition with mitoxantrone.	IC_50_ values of mitoxantrone (alone) is 199 ± 19.3 µM, while in combination with naringenin IC_50_ value is reduced to 1.23 ± 0.16 µM.	
*Citrus sinensis* (Family: Rutaceae)	Hesperidin (**95**), Hesperitin (**96**)	Not found.	Hesperidin reverses doxorubicin resistance in Caco-2 Cell line via p-gp inhibition.	IC_50_ value of doxorubicin is reduced to 194.89 ± 43.87 µM in presence of hesperidin.	[147,148]
			Hesperitin enhances vincristine uptake in BBB via P-gp modulation and it was tested in mouse brain capillary endothelial cells (MBEC4 cells).	Not found.	
*Coffea Arabica* (Family: Rubiaceae)	Chlorogenic acid (**97**)	Inhibits P-gp ATPase activity.	Inhibits P-gp function in jejunal mucosa of rats.	Not found.	[149]
*Curcuma ecalcarata* (Family: Zingiberaceae)	Pinocembrine (**98**)	Inhibits P-gp protein expression.	Iinhibits P-gp expression in BBB tested in cultured rat brain microvascular endothelial cells (rBMECs).	Not found.	[150,151]
*Curcuma longa* (Family:Zingiberaceae)	Tetrahydro-curcumin (**99**)	Shows concentration dependent decreased P-gp protein and MDR1 gene expressions.	Inhibits the efflux function of P-gp, MXR and MRP1 in drug resistance KB-V-1, MCF7AdrVp 3000 and MRP1-HEK 293 cell lines and enhances the cytotoxicity of vinblastine, mitoxantrone and etoposide.	IC_50_ values for vinblastine, mitoxantrone and etoposide in combination with Tetrahydro-curcumin are reduced to 0.7 ± 0.2, 14.6 ± 2.8 and 11.9 ± 2.8 µM, respectively.	[152,153]
	Curcumin (**100**)		Curcumin inhibits P-gp function in MDR K562/A02 cells.	Not found.	
*Dalea spinosa* (Family: Fabaceae)	Pterocarpan (**101**)	Inhibits NorA efflux pump.	Enhances antimicrobial activity of berberine in resistant strains of *Staphylococcus aureus*.	Not found.	[154]
*Dorstenia barteri* (Family: Moraceae)	Isobavachalcone (**102**)	Inhibits AcrAB and TolC efflux pumps.	Inhibits MDR efflux pumps in gram negative bacteria.	Not found.	[155]
*Eriodictyon californicum* (Family: Boraginaceae)	Eriodictoyl (**103**)	Not found.	Acts as P-gp inhibitor	Not found.	[5]
*Fragaria ananassa* (Family: Rosaceae), *Dimorphandra mollis* (Family: Fabaceae)	Rutin (**104**)	Inhibits bacterial TetK efflux pump.	Enhances isoniazid activity against *Mycobacterium smegmatis* mc2155.	Not found.	[156]
*Genista tinctoria* (Family: Fabaceae)	Genistein (**105**)	Inhibits BCRP protein expression.	In combination with mitoxantrone shows significant inhibition of mitoxantrone efflux in MCF-7 MS100 cells. Inhibit the labeling of P-gp with its photoactive substrate.	IC_50_ values of mitoxantrone (alone) is 199 ± 19.3 µM, while in combination with genistein IC_50_ value is reduced to 2.29 ± 0.86 µM.	[138,157]
*Ginkgo biloba* (Family: Ginkgoaceae), *Citrus paradisi* (Family: Rutaceae)	Bergamottin (**106**) 6′,7′-dihydroxy bergamottin (**107**)	Not found.	Inhibit the P-gp substrate saquinavir transport in human liver microsomes.	IC_50_ values for saquinavir along with the compounds are 0.74 ± 0.13 µM and 0.33 ± 0.23 µM, respectively.	[158]
*Epimedium grandiflorum* (Family: Barberidaceae)	Icaritin (**108**)	Down-regulates the expression of P‑gp via decreasing the expression of the MDR1 gene.	Significantly increases the cytotoxicity of adriamycin, vincristine, cisplatin and 5‑fluorouracil in MDR HepG2/ADR (liver cancer cell line).	IC_50_ values for adriamycin, vincristine, cisplatin and 5‑fluorouracil are reduced to 0.596 ± 0.063, 0.267 ± 0.034, 1.285 ± 0.125 and 63.092 ± 2.174 µg/L, respectively.	[159]
*Herissantia tiubae* (Family: Malvaceae)	Tiliroside (**109**)	Inhibits NorA efflux protein expression.	Potentiates antimicrobial activities of norfloxacin, ciprofloxacin, lomefloxacin and ofloxacin in *Staphylococcus aureus* (SA1199B).	Not found.	[160]
*Humulus lupulus* (Family: Cannabaceae)	8-prenyl naringenin (**110**)	Inhibits transport of MRP1 in human erythrocytes.	Inhibits MRP1 mediated transport of fluorescent substrate BCECF. It also acts as an effective inhibitor of Rh 123 transport in doxorubicin resistant human adenocarcinoma cell line (LoVo/Dx cells).	IC_50_ value for 8-prenyl naringenin for MRP1 is 5.76 ± 1.80 µM.	[161,162]
*Hypericum perforatum* (Family: Clusiaceae)	Hypericin (**111**)	Not found.	Inhibits P-gp function on human doxorubicin-resistant adenocarcinoma cell line (LoVo DX).	Not found.	[157]
*Kaempferia parviflora* (Family: Zingiberaceae)	5,7-Dimethoxy flavone (**112**)	Inhibits BCRP protein expression.	Intracellular concentration of mitoxantrone is significantly increased in MDCK/Bcrp1 and MDCK/BCRP cells when co administered with 5,7-dimethoxyflavone.	Not found.	[163]
*Larix gmelinii* (Family: Pinaceae), *Sophora japonica* (Family: Fabaceae)	Taxifolin (**113**)	Not found.	Enhances isoniazid activity in *Mycobacterium smegmatis* mc2155 via inhibiting bacterial TetK efflux pump.	Not found.	[156]
*Maclura pomifera* (Family: Moraceae), *Psidium guajava* (Family: Myrtaceae)	Morin (**114**)	Inhibits P-gp ATPase via binding to the ATP-binding site.	Increases accumulation of daunomycin in P-gp overexpressing MCF-7/Adr cells.	Not found.	[137]
*Malus domestica* (Family: Rosaceae)	Phloretin (**115**), Phloridzin (**116**)	Inhibit P-gp ATPase via binding to the ATP-binding site.	Increases accumulation of daunomycin in P-gp overexpressing MCF-7/Adr cells.	Not found.	[137]
*Mangifera indica* (Family: Anacardiaceae)	Rhamnetin (**117**)	Inhibits Notch-1 signaling pathway and P-gp related protein expression.	Enhances the performance of adriamycin, etoposide, paclitaxel and sorafenib in MDR hepatocellular carcinoma cell line (HepG2/ADR).	IC_50_ values of these drugs in presence of rhamanetin are reduced to 1.74 ± 0.14, 0.12 ± 0.03, 0.05 ± 0.01, 0.82 ± 0.15 μM, respectively.	[164]
*Marchantia polymorpha* (Family: Marchantiaceae)	Plagiochin E (**118**)	Inhibits Cdr1p efflux pump and mRNA expression of efflux transporter gene (CDR1).	Inhibits azole resistance in *Candida albicans* and potentiate the antimicrobial activity of fluconazole.	Not found.	[165]
*Mentha piperita* (Family: Lamiaceae)	Spiraeoside (**119**)	Not found.	Inhibits talinolol efflux out of the Caco-2 cell monolayers via P-gp inhibition.	Not found.	[134]
*Momordica dioica* (Family: Cucurbitaceae)	Daidzin (**120**)	Stimulates ATPase activity and inhibits BCRP expression.	Enhances the accumulation of two BCRP substrates, mitoxantrone and bodipy-FL-prazosin in mitoxantrone selected BCRP-overexpressing epithelial breast cancer cell line (MCF/MR) via inhibiting P-gp function.	Not found.	[135,166]
*Myristica fragrans* (Family: Myristiaceae)	Myricetin (**121**)	Stimulates ATPase activity and inhibits MRP1 expression.	Enhances the cellular accumulation of Rh 123 in MCF-7/Adr cells and enhances doxorubicin oral bioavailability in rats.	Not found.	[167,168]
			Inhibits MRP1 mediated BCECF efflux out of human erythrocytes.	IC_50_ value of doxorubicin along with myricetin is reduced to 52.6 ± 2 µM.	
*Osmundea pinnatifida* (Family: Rhodomelaceae)	Scutellarein (**122**), Scutellarein 4′-methyl ether (**123**)	Not found.	Inhibitory effect of scutellarin on P-gp activity were examined on a human metastatic malignant melanoma cell line, WM-266-4, by calcein-AM fluorometry screening assay.	Not found.	[168,169,170]
*Passiflora caerulea* (Family: Passifloraceae)	Chrysin (**124**)	Inhibits BCRP protein expression.	In combination with mitoxantrone shows significant P-gp inhibition in MCF-7MS-100 cells.	IC_50_ values of mitoxantrone (alone) is 199 ± 19.3µM, while in combination with chrysin IC_50_ value is reduced to 1.13 ± 1.11 µM.	[138]
*Pinus massoniana* (Family: Pinaceae), *Citrus paradise* (Family: Rutaceae)	Procyanidine (**125**)	Inhibits P-gp ATPase in BBB. Reverses P-gp associated MDR by inhibiting the function and expression of P-gp through down-regulation of NF-κB activity and MAPK/ERK pathway mediated YB-1 nuclear translocation.	Inhibits P-gp in BBB and acts on cerebral tumors.	Not found.	[171,172]
			Procyanidine potentiates paclitaxel and adriamycin concentration in MDR human ovarian cancer cell line (A2780/T).	IC_50_ values of paclitaxel and adriamycin are reduced to 11.36 ± 1.13 and 6.30 ± 0.38 µM, respectively.	
*Prunus armeniaca* (Family: Rosaceae)	Isoquercetin (**126**)	Reduces mRNA expression of P-gp.	Reduces P-gp expression.	Not found.	[5]
*Robinia pseudoacacia* (Family: Fabaceae)	Acacetin (**127**), Robinin (**128**)	Stimulate ATPase activity and inhibits MRP1 expression. Also act as natural substrates of BCRP and competitively inhibits BCRP-mediated drug efflux. Inhibits P-gp via synergism with substrate.	Inhibit MRP1 mediated BCECF efflux in human erythrocytes. Show P-gp inhibition in K562/BCRP cell line and potentiate mitoxantrone, SN-38, topotecan accumulation. Potentiate activity of epirubicin in MDR protein-expressing human breast cancer cell line (MDA-MB-231).	IC_50_ value for acacetin in human erythrocyte is 6.5 ± 4 µM/L. ID_50_ values for robinin + epirubicin is 0.02 µg/mL	[125,168,173]
*Salicornia herbacea* (Family: Amaranthaceae), *Hippophae rhamnoides* (Family: Elaeagnacae)	Isorhamnetin (**129**)	Co-transporting of isorhamnetin across Caco-2 cells monolayer may cause competitive substrate inhibition of P-gp, MRP-2 and BCRP. Also inhibits bacterial TetK efflux pump.	It inhibited P-gp function in Caco-2 cells. It also enhanced isoniazid activity in *Mycobacterium smegmatis* mc2155	Not found.	[156,174]
*Sasa borealis* (Family: Poaceae)	Tricin (**130**)	Not found.	Shows inhibitory effects on the P-gp in adriamycin-resistant human breast cancer cells, MCF-7/Adr.	Not found.	[175]
Several plant species under Fabaceae family	Rotenone (**131**), Formononetin (**132**), Afrormosin (**133**)	Inhibit P-gp via synergism with substrate.	Rotenone potentiates the activity of epirubicin in human mdr1 gene-transfected mouse lymphoma cell line.	ID_50_ value for rotenone + epirubicin is 0.006 µg/mL.	[125]
			Formononetin potentiates the activity of epirubicin in MRP-expressing human breast cancer cell line (MDA-MB-231).	ID_50_ value for formononetin + epirubicin is 0.02 µg/mL.	
			Afrormosin potentiates the activity of epirubicin in MRP-expressing human breast cancer cell line (MDA-MB-231).	ID_50_ value for afrormosin + epirubicin is 0.06 µg/mL.	
*Scutellaria baicalensis* (Family: Lamiaceae)	Wogonin (**134**)	Not found.	Potentiates antitumor action of etoposide through inhibition of its efflux via P-gp tranporters in Jurkat cells and A549 cells.	Not found.	[176]
	Baicalein (**135**)	Not found.	Inhibits P-gp efflux pump in the small intestine.	Not found.	[139,177]
		Inhibits TetK efflux pump.	Potentiates antimicrobial activity of ethidium bromide on. *Mycobacterium smegmatis.*	Not found.	
*Silybum marianum* (Family: Asteraceae)	Silymarin (**136**), Silybin (**137**)	Not found.	Silymarin increases daunomycin accumulation in P-gp positive cells.	Not found.	[137,178,179]
			Silymarin also inhibits P-gp mediated digoxin and vinblastine transport in Caco-2 cell line.	Not found.	
			Doxorubicin cytotoxicity in MDA435/LCC6MDR1 cell line was increased by silymarin via inhibition of P-gp.	IC_50_ value of doxorubicin is reduced to 8.74 ± 5.88 µM in MDA435/LCC6MDR1 cells.	
			In human prostate carcinoma DU145 cells, silybin potentiates doxorubicin induced toxicity.	Not found.	
*Thalassia testudinum* (Family: Hydrocharitaceae)	Luteolin (**138**), Luteolin-4′-*O*-glucoside (**139**)	Not found.	Show P-gp inhibition in K562/BCRP cells and potentiate mitoxantrone, SN-38, topotecan accumulation.	Not found.	[173]
Various plant species	Apigenin (**140**)	Inhibits BCRP protein expression.	Shows significant inhibition of mitoxantrone efflux in MCF-7 MS100 cells.	In combination IC_50_ value of mitoxantrone is 1.73 ± 1.42 µM	[138]
*Vicia orobus* (Family: Fabaceae)	Diosmetin (**141**)	Not found.	Inhibits P-gp in BBB and is determined in PBCECs (porcine brain capillaries and capillary endothelial cells) by the calcein assay. It also produces cytotoxicity in CEM/Adr5000 cell line via P-gp inhibition.	EC_50_ value of calcein in PBCEC is reduced to 16.3 ± 8.2 µg/L.IC_50_ value in CEM/ADR5000 cell is reduced to 3.5 ± 1.3 µg/L.	[180]
*Vitis vinífera* (Family: Vitaceae)	Resveratrol (**142**)	Not found.	Promotes fexofenadin absorption in rat intestine and bioavailability of nicardipine via P-gp inhibition. It increases accumulation of daunorubicin in KB-C2 cells. It inhibits the P-gp activity in P-gp overexpressing MCF-7/Adr cells. Cytotoxicity of vincristine, adriamycin and paclitaxel was enhanced by reveratrol on KBv200 cells.	Not found.	[181,182,183,184]
**Terpenoids**					
*Bipolaris leersiae* (Family: Pleosparaceae)	Ophiobolin A (**143**)	Not found.	Potentially Inhibites P-gp mediated efflux of [3H] digoxin in LLCGA5-COL150 cells.	Not found.	[6]
*Cecropia lyratiloba* (Family: Moraceae)	Euscaphic acid (**144**), tormentic acid (**145**), 2-alpha-acetyl tormentic acid (**146**), 3-beta-acetyl tormentic acid (**147**)	Inhibit expression of P-gp and reverse MDR.	Show reversal of MDR activity in MDR leukemia cells (K562/VCR).	Not found.	[185]
*Clavija procera* (Family: Theophrastaceae)	Aegicerin (**148**)	Not found.	Shows reversal of MDR activity in resistant *Mycobacterium tuberculosis*.	Not found.	[186]
*Crocus sativus* (Family: Iridaceae)	Safranal (**149**)	Not found.	Potentially Inhibits P-gp mediated efflux of [3H] digoxin in LLCGA5-COL150 cells.	Not found.	[6]
*Cymbopogon citrus* (Family: Poaceae)	Citral (**150**)	Directly inhibits MRP1 and MRP2 via binding with their active sites.	A significant inhibition is observed in both MRP1 and MRP2 in isolated Sf9-MRP1- and Sf9-MRP2-membrane vesicles.	Not found.	[187]
*Eucalyptus dives* (Family: Myrtaceae)	Piperitone (**151**)	Not found.	Inhibits P-gp mediated efflux of [3H] digoxin in LLCGA5-COL150 cells.	Not found.	[6]
*Euphorbia dendroides* (Family: Euphorbiaceae)	Euphodendroidin D (**152**)	Inhibits P-gp activity via binding with its active sites.	Prevents daunomycin efflux from K562/R7 human leukemic cells via P-gp inhibition.	Not found.	[188]
*Euphorbia lagascae* (Family: Euphorbiaceae)	Jolkinol D (**153**)	Inhibits P-gp activity via binding with its active sites.	Enhances doxorubicin cytotoxicity synergistically on human MDR-1 gene transfected mouse lymphoma cells.	IC_50_ value of doxorubicin is reduced to 0.26 ± 0.05 µM.	[189]
*Euphorbia lagascae* (Family: Euphorbiaceae)	Latilagascene B (**154**), Latilagascene E (**155**), Latilagascene D (**156**)	All compounds inhibit P-gp mediated MDR via directly blocking its active sites.	Latilagascene B, latilagascene E and latilagascene D show synergistic activity with doxorubicine on MDR-1 gene-transfected L1210 mouse lymphoma cells.	Not found.	[190]
*Euphorbia mellifera* (Family: Euphorbiaceae)	Euphomelliferine A (**157**), Euphomelliferine (**158**)	Inhibit P-gp activity via binding with its active sites.	Successfully inhibit P-gp activity on human MDR colon adenocarcinoma (MDR COLO-320) and human MDR1 gene transferred mouse (L5178Y MDR) cells.	Not found.	[191]
*Euphorbia paralias* (Family: Euphorbiaceae)	Paraliane (**159**)	Inhibits P-gp activity via binding with its active sites.	Shows P-gp inhibitory action on MDR-1 gene-transfected L1210 mouse lymphoma cells.	Not found.	[192]
*Euphorbia peplus* (Family: Euphorbiaceae)	Pepluanin A (**160**)	Inhibits P-gp activity via binding with its active sites.	Shows P-gp inhibitory action on MDR-1 gene-transfected L1210 mouse lymphoma cells; promotes Rh 123 and epirubicin accumulation. Pepluanin A prevents daunomycin efflux from K562/R7 human leukemic cells via P-gp inhibition.	Not found.	[188,192]
*Euphorbia piscatoria* (Family: Euphorbiaceae)	Jolkinol B (**161**)	Inhibits P-gp activity via binding with its active sites.	Shows P-gp inhibition on human MDR-1 gene transfected and parenteral L5178 mouse lymphoma cells.	Not found.	[190]
*Euphorbia portlandica* (Family: Euphorbiaceae)	Euphoportlandol A (**162**), Euphoportlandol B (**163**)	Inhibit P-gp activity via binding with its active sites.	Reversal of MDR was evaluated via Rh 123 exclusion in L5178 mouse lymphoma cells transfected with the pHa MDR1/A gene.	Not found.	[193]
*Euphorbia* spp. (Family: Euphorbiaceae)	Helioscopinolide A (**164**), Helioscopinolide B (**165**), Helioscopinolide E (**166**), Helioscopinolide F (**167**)	Inhibit P-gp activity via binding with its active sites.	Exhibit high anti-neoplastic activity against human MDR-1 gene-transfected mouse lymphoma cells.	Not found.	[194]
*Euphorbia tuckeyana* (Family: Euphorbiaceae)	Tuckeyanols A (**168**), Tuckeyanols B (**169**), Euphotuckeyanol (**170**)	Inhibit P-gp activity via binding with its active sites.	All show P-gp inhibition on human MDR-1 gene transfected and parenteral L5178 mouse lymphoma cells and potentiate epirubicin action.	Not found.	[195]
*Gentiana verna* (Family: Gentianaceae)	Loganine (**171**)	Not found.	Inhibits P-gp mediated efflux of [3H] digoxin in LLCGA5-COL150 cells.	Not found.	[6]
*Glycyrrhiza glabra* (Family: Fabaceae)	Glycyrrhizin (**172**),	Inhibit P-gp ATPase activity.	Inhibits P-gp in MDR1-MDCKII and Caco-2 cell.	21.78 µM	[196]
		Not found.	Potentially Inhibits P-gp mediated efflux of [3H] digoxin in LLCGA5-COL150 cells.	Not found.	[6]
*Laurencia fliformis* (Family: Rhodomelaceae)	Parguerene I (**173**), Parguerene II (**174**)	Inhibits both P-gp and MRP1.	Both show reversal of vinblastine, doxorubicin and paclitaxel resistance in SW620 AD-300, HEK293/ABCB1, CEM/VLB100 cells.	Not found.	[7]
*Licania tomentosa*, *Chrysobalanus icaco* (Family: Chrysobalanaceae)	Betulinic acid (**175**), pomolic acid (**176**)	Not found.	Inhibit the proliferation of a vincristine-resistant derivative of K562 cells and reduced MDR activity.	Not found.	[197]
*Maytenus* spp. (Family: Celastraceae)	Dihydro-β-agarofuran (**177**)	Similar to verapamil.	It is found to inhibit leucine uptake in LNCaP cells. It also shows higher P-gp reversal activity on human MDR1-transfected NIH-3T3 cells. Dihydro-β-agarofuran was observed as an inhibitor of a P-gp like transporter in multidrug-resistant *Leishmania tropica.*	Not found.	[198,199,200]
*Olea europea* (Family: Oleaceae)	Oleanolic acid (**178**)	Not found.	Potentially inhibits P-gp mediated efflux of [3H] digoxin in LLCGA5-COL150 cells.	Not found.	[6]
*Origanum vulgare* (Family: Lamiaceae)	Carvacrol (**179**)	Not found.	Potentially inhibits P-gp mediated efflux of [3H] digoxin in LLCGA5-COL150 cells.	Not found.	[6]
*Pastinaca sativa* (Family: Apiaceae)	Terpinolene (**180**)	Not found.	Potentially inhibits P-gp mediated efflux of [3H] digoxin in LLCGA5-COL150 cells.	Not found.	[6]
*Phellodendron amurense* (Family: Rutaceae)	Obacunone (**181**)	Shows P-gp mediated MDR inhibition activity.	Shows P-gp inhibition on MES-SA/DX5 (human MDR uterine sarcoma cells) and HCT15 cells (Human colorectal cancer cell line).	ED_50_ values are 0.028 and 0.0011 µg/mL, respectively.	[201]
*Phellodendron amurense* (Family: Rutaceae)	Limonin (**182**)	Shows P-gp mediated MDR inhibition activity.	Shows P-gp inhibition on MES-SA/DX5 (human MDR uterine sarcoma cells) and HCT15 cells (Human colorectal cancer cell line)	ED_50_ values are 0.021 and 0.392 µg/mL, respectively.	[100,201]
*Pinus nigra* (Family: Pinaceae)	Isopimaric acid (**183**)	Inhibits microbial TetK or NorA efflux pumps.	Potentiate antibiotic activity in *Staphylococcus aureus.*	Not found.	[202]
	β-myrcene (**184**)	Not found.	Potentially inhibits P-gp mediated efflux of [3H] digoxin in LLCGA5-COL150 cells.	Not found.	[6]
*Podocarpus totara* (Family: Podocarpaceae)	Totarol (**185**)	Inhibits NorA efflux pump.	Inhibits *Staphylococcus aureus* NorA efflux pump.	Not found.	[203]
*Sinocalycanthus chinensis* (Family: Calycanthaceae)	Sinocalycanchinensin E (**186**)	Not found.	Shows reversal of MDR activity in MDR KB cells and enhanced colchicines induced cytotoxicity.	Not found.	[204]
*Siphonochalina siphonella* (Family: Callyspongidae)	Sipholenol A (**187**), Sipholenol L (**188**)	Show P-gp mediated MDR inhibition activity.	Enhance cytotoxicity of P-gp substrate in KB C2 cells and human cervix carcinoma subclone derived from KB-3 1cells via P-gp inhibition.	Not found.	[205,206]
*Tamarindus indica* (Family: Fabaceae)	Lupeol (**189**)	Not found.	Potentially inhibits P-gp mediated efflux of [3H] digoxin in LLCGA5-COL150 cells.	Not found.	[6]
*Thymus vulgaris* (Family: Lamiaceae)	Thymol (**190**)	Not found.	Potentially inhibits P-gp mediated efflux of [3H] digoxin in LLCGA5-COL150 cells.	Not found.	[6]
*Zanthoxylum piperitum* (Family: Rutaceae)	Citronellal (**191**), Citronellol (**192**)	Not found.	Potentially inhibits P-gp mediated efflux of [3H] digoxin in LLCGA5-COL150 cells.	Not found.	[6]
**Saponins, Sapogenins and Sterols**					
*Astragalus membranaceus* (Family: Fabaceae)	Astragaloside II (**193**)	Downregulates the expression of the P-gp and MDR1 genes.	Astragaloside II in low concentration shows strong potency to increase 5-fluorouracil cytotoxicity toward 5-fluorouracil-resistant human hepatic cancer cells Bel-7402/FU. It also downregulates the P-gp and MDR1 genes.	Not found.	[207]
*Citrus jambhiri*, *Citrus pyriformis* (Family: Rutaceae)	Stigmasterol (**194**), β-sitosterol-*O*-glucoside (**195**)	Not found.	Inhibit P-gp in Caco-2 cells and enhance accumulation of Rh 123.	Not found.	[148]
*Cucurbita andreana, Hemsleya endecaphylla, Ecballium elaterium, Citrullus colocynthis* etc. (Family: Cucurbitaceae)	Cucurbitacin I (**196**)	Not found.	Potentially inhibits P-gp mediated efflux of [3H] digoxin in LLCGA5-COL150 cells.	Not found.	[6]
*Dioscorea opposite* (Family: Dioscoreaceae)	Gracillin (**197**)	Inhibits P-gp via direct interaction with active binding sites.	Shows inhibition of P-gp mediated daunorubicin efflux in K567/R7 cells (human leukemic).	Not found.	[208]
*Isis hippuris* (Family: Isididae)	Gorgosterol (**198**), Hippuristanol (**199**)	Not found.	Show P-gp inhibitory activity on KB-C2 cells.	Not found.	[7]
*Labisia pumila* (Family: Primulaceae)	Primulanin (**200**)	Not found.	Inhibits P-gp mediated drug efflux, tested in hMDR1-MDCKII cells using ^3^H-digoxin.	IC_50_ value is 6.4 ± 2.3 µM.	[209]
*Marsdenia tenacissima* (Family: Apocynaceae)	Tenacissimoside A (**201**)	Modulates P-gp mediated MDR through direct interaction with P-gp substrate site.	Reverses MDR in P-gp overexpressing MDR cancer cells (HepG2/Dox). The sensitivity of HepG2/Dox cells to antitumor drugs doxorubicin, vinblastine, puromycin and paclitaxel was increased by 18-, 10-, 11- and 6-fold by 20 µg/mL (or 25 µM) in presence of tenacissimoside A.	Not found.	[210]
*Momordica balsamina* (Family: Cucurbitaceae)	Karavilagenin C (**202**), Balsaminol (**203**), balsaminagenin B (**204**), balsaminoside A (**205**)	Karavilagenin C inhibits Rv1258c efflux pump. Karavilagenin, balsaminol and balsaminagenin inhibit AcrAB-TolC efflux pump. Show synergistic interactions with P-gp substrate and inhibit P-gp function	Karavilagenin C enhances antimicrobial activity of ethidium bromide *on Enterococcus faecalis*. Karavilagenin, balsaminol and balsaminagenin potentiate actimicrobial activity in *S. aureus* and *E. coli*. Balsaminagenin B, balsaminoside A and karavilagenin C show MDR reversing activity on human MDR1 gene transfected mouse lymphoma cells and enhance cytotoxicity of doxorubicin.	Not found.	[211,212]
*Panax ginseng* (Family: Araliaceae)	Protopanaxatriol (**206**), 20(*S*)-ginsenoside F_1_ (**207**)	Protopanaxatriol directly inhibits P-gp mediated substrate transport. Ginsenoside F_1_ inhibits P-gp ATPase activity.	Directly inhibit P-gp in daunorubicin- and doxorubicin-resistant acute myelogenous leukemia sublines (AML-2/D100 and AML-2/DX100) and enhance daunorubicin concentration inside cell. 20(*S*)-ginsenoside F_1_ shows P-gp inhibitory activity on MDR1-MDCKII and Caco-2 cells.	Not found.	[196,213]
*Paris polyphylla* (Family: Melianthaceae)	Pennogenine (**208**)	Inhibits P-gp via direct interaction with active binding sites.	Inhibits P-gp-mediated daunorubicin efflux in K562/R7 cells.	Not found.	[208]
*Tacca chantrieri* (Family: Dioscoreace)	Teccalonolides A (**209**), Teccalonolides E (**210**), Teccalonolides B (**211**), Teccalonolides N (**212**)	Not found.	All of the teccalonolides are active against P-gp expressed and MRP7 transfacted MDR cancer cells. Taccalonolides A and E are highly active in vivo against a doxorubicin- and paclitaxel- resistant P-gp-expressing tumor (Mam17/ADR) and also bind with tubuline.	Not found.	[214]
*Spongia* spp. (Family: Spongiidae)	Agosterol A (**213**)	Inhibits ATP-dependent drug efflux by P-gp and MRP1.	Reverses the resistance to colchicine in KB-C2 cells and also reverses the resistance to vincristine in KBCV60 cells via P-gp and MRP inhibition.	Not found.	[7,215]
*Trillium tschonoskii* (Family: Trilliaceae)	Paris saponin VII (**214**)	Inhibits P-gp ATPase activity.	It reverses MDR in adriamycin-resistant MCF-7/ADR cells and intracellular Rh 123 accumulation is increased via P-gp inhibition.	Not found.	[195]
Vegetables oils, legumes, nut, seeds	β-sitosterol (**215**), *Z*-guggulsterone (**216**)	Not found.	β-sitosterol shows P-gp inhibitory activity in multidrug resistant NCI/ADR-RES cell line. Z-guggulsterone enhances accumulation of daunorubicin or Rh 123 in P-gp-overexpressing human carcinoma KB-C2 cells and human MRP1 gene-transfected KB/MRP cells via P-gp inhibition.	Not found.	[216]
*Vitex scabra* (Family: Verbenaceae)	Pinnatasterone (**217**)	Inhibits P-gp via direct interaction with active binding sites.	Shows inhibition of P-gp-mediated daunorubicin efflux in K562/R7 (human leukemic) cells.	Not found.	[195]
**Coumarins**					
*Angelica gigas* (Family: Apiaceae)	Decursinol (**218**)	Inhibits P-gp, MRP-2 and BCRP via acting as substrate.	Acts on Caco-2 cell monolayer and inhibites efflux transporter like BCRP/MDP 2.	Not found.	[217]
*Calophyllum brasillense* (Family: Clusiaceae)	GUT 70 (**219**)	Inhibits P-gp expression.	Acts by inhibiting the P-gp activity at human leukemia cells.	2–5 µM	[218]
*Citrus hybrids* (Family: Rutaceae)	Bergamottin (**220**), 6′,7′-dihydroxy bergamottin (**221**), 6′,7′-epoxy bergamottin (**222**)	Inhibit P-gp expression.	Inhibit P-gp (ABCB1) mediated transport of talinolol in Caco-2 cells.	Not found.	[143,144]
*Citrus paradisi* (Family: Rutaceae)	Bergaptol (**223**)	Specific inhibitor of P-gp and/or MRP2 function.	Inhibited [^3^H]-Vinblastine efflux from LLC-GA5-COL300 cells (a transformant cell line derived by transfecting LLC-PK1 with human MDR_1_ cDNA isolated from normal adrenal gland). Also inhibites P-gp function in human breast cancer cells.	Not found.	[219,220]
*Ferula persica* (Family: Umbelliferae)	Farnesiferol A (**224**), Farnesiferol B (**225**), Farnesiferol C (**226**), Lehmferin (**227**)	Inhibit P-gp active substrate binding sites.	Farnesiferol A is observed as a potential P-gp inhibitor tested via Rh 123 efflux assay in doxorubicin resistant breast cancer cell line (MCF7/Adr). Farnesiferol B, farnesiferol C and lehmferin inhibit P-gp efflux pump and enhanced performance of doxorubicin on breast cancer cell line (MCF7/ADR).	IC_50_ values for doxorubicin + farnesiferol B is 10.68 μM, doxorubicin + farnesiferol C is 6.72 μM and doxorubicin + lehmferin is 5.08 μM	[221,222]
*Ferula schtschurowskiana* (Family: Umbelliferae)	Conferone (**228**)	Inhibits P-gp via competitive binding with P-gp active sites.	Inhibits efflux of vinblastine in MDCK-MDR1 cells.	Not found.	[223]
*Ferula szowitsiana* (Family: Umbelliferae)	Galbanic acid (**229**)	Inhibits P-gp via competitive binding with P-gp active sites. Also inhibits NorA or NorB efflux pump	Galbanic acid is observed as a potential P-gp inhibitor tested via Rh 123 efflux assay in doxorubicin resistant breast cancer cells (MCF7/Adr). Enhances the performance of Ethidium bromide in *Staphylococcus aureus*.	Not found.	[222,224]
*Peucedanum praeruptorium* (Family: Oenanthe)	Praeruptorin A (**230**)	Inhibits P-gp fuction via depleting ATP and/or suppressing P-gp gene expression.	Inhibits P-gp mediated drug resistance for doxorubicin, paclitaxel, puromycin and vincristine in MDR human oral epidermoid carcinoma cells (KB-V1).	Not found.	[225]
*Tordylium opulum* (Family: Apiaceae)	Cnidiadin (**231**)	Acts as chemo-sensitiser for P-gp and inactivates it via blocking its efflux function.	Enhances vinblastine or vincristine performance in two cell lines overexpressing P-gp namely, MDCK-MDR1 and KB/VCR cells.	Not found.	[226]
**Peptides**					
*Discodermia dissoluta* (Family: Theonellidae)	Discodermolide (**232**)	Not found.	Reverses the resistance of paclitaxal in ovarian carcinoma cells (A2780AD).	580 nM.	[227]
			Reverses paclitaxal resistance in colon carcinoma cells (SW620AD-300).	70 nM.	
*Haliclona caerulea* (Family: Chalinidae)	Kendarimide (**233**)	Reverses P-gp mediated MDR.	Reverses the resistance to colchicin in human carcinoma cells (KB-C2).	Not found.	[228]
*Hapalosiphon welwitschii* (Family: Hapalosiphonaceae)	Hapalosin (**234**)	Reverses P-gp mediated efflux via direct inhibition of efflux mechanism.	Reverses MDR in P-gp overexpressing, vinblastine-resistant human ovarian adenocarcinoma cell line with higher effect than the known P-gp inhibitor verapamil.	Not found.	[229]
*Nocardiopsis* spp. (Family: Nocardiopsaceae)	Nocardioazine A (**235**)	Inhibits membrane bound P-gp efflux protein.	Reverses the resistance to doxorubicin in SW620AD-300 cells.	Not found.	[7,230]
**Resins**					
*Garcinia hamburyi* (Family: Clusiaceae)	Gambogic acid (**236**)	Dose dependently inhibits ABCB1 activity. It directly inhibits ABCB1 and protein degradation of ABCB1 via the proteasome pathway.	In the MCF-7/Adm cells, gambogic acid enhances the cytotoxicities of docetaxel and adriamycin.	Not found.	[231]
*Ipomoea violacea* (Family: Convolvulaceae)	Orizabin (**237**)	Inhibits NorA efflux pump.	Reverses norfloxacin resistance in *Staphylococcus aureus.*	Not found.	[232]
**Miscellaneous**					
*Alpinia galangal* (Family: Zingiberaceae)	Acetoxy cavicolacetate (**238**)	Inhibits NorA efflux pump.	Potentiates the activity of ethidium bromide in *Staphylococcus aureus.*	Not found.	[233]
*Arctium lappa* (Family:Astereceae)	Arctigenin (**239**), Matairesinol (**240**), Arctiin (**241**), Isolappaol A (**242**), Lappaol C (**243**), Lappaol F (**244**)	Show synergistic activity with the cytotoxic drugs.	Potentiate doxorubicin mediated cytotoxicity in CaCo2 and CEM/ADR5000 cell lines.	Not found.	[234]
Ascidian of genus *Didemnun* (Family: Didemnidae)	Ningalin B (**245**)	Not found.	Inhibits P-gp function in P-gp overexpressing human colorectal carcinoma (HCT116/VM46) cells and increases sensitivity to vinblastine and doxorubicin.	Not found.	[235]
*Berberis aetnensis* (Family: Berberidaceae)	Porphyrin (**246**), pheophorbide A (**247**)	Pheophorbide A inhibits MexAB-OprM efflux pump. Porphyrin and pheophorbide also inhibit NorA efflux.	Pheophorbide A enhances the activity of Ciprofloxacin in *Pseudomonas aeruginosa.* Porphyrin and pheophorbide enhance the activities of ciprofloxacin and norfloxacin.	Not found.	[236]
*Bugula neritina* (Family: Bugulidae)	Bryostatin 1 (**248**)	Not found.	Reverses resistance of colchinin in KB-1 and human epitheliod cervix carcinoma cells.	Not found.	[237]
*Cannabis sativa* (Family: Cannabaceae)	Cannabinol (**249**), Cannabidiol (**250**)	Inhibit P-gp ATPase activity. Alter P-gp and BCRP mRNA expression.	Both stimulate the activity of ATPase activity of P-gp. On other hand, cannabidiol inhibited the verapamil stimulated ATPase activity of P-gp. Rh 123 and cyclosporine A accumulation is increased by cannabidiol in MCF-7/P-gp and choriocarcinoma cells.	Not found.	[238,239]
*Cinnamonum camphora* (Family: Lauraceae)	Anethole (**251**)	Not found.	Inhibites P-gp efflux in hepatocellular carcinoma cells.	Not found.	[234,240]
*Eugenia caryophyllus* (Family: Myrtaceae)	Eugenol (**252**)	Not found.	Inhibites P-gp efflux in hepatocellular carcinoma cells.	Not found.	[241]
*Geranium caespitosum* (Family: Geraniaceae)	Polyacylated neohesperidosides (**253**)	Inhibits NorA Efflux pump.	Enhances the activities of ciprofloxacin, norfloxacin, rhein, berberine in *Staphylococcus aureus.*	Not found.	[242]
*Nicotiana tabacum* (Family: solanaceae), *Dalea versicolor* (Family: Fabaceae)	Chalcone (**254**)	Inhibits NorA efflux pump.	Reverses resistance of berberine, erythromycin and tetracycline in *Bacillus cereus* and *Staphylococcus aureus.*	Not found.	[243]
*Prunella vulgaris* (Family: Lamiaceae)	Crude extract	Not found.	Inhibits *Ebola* virus glycoprotein (GP)-mediated virus entry and infection in different cell lines like HUVEC and macrophage.	Not found.	[244]
*Schisandra chinensis* (Family: Schisandraceae)	Gomisin (**255**), Pregomisin (**256**)	Act as uncompetitive inhibitor for P-gp-ATPase activity.Alter P-gp substrate interactions, noncompetitively	Show MDR phenomenon on human hepG2 hepatoma cells.	Not found.	[245]
*Zingiber officinalis* (Family: Zingiberaceae)	Phenylbutanoids (**257**)	Inhibits P-gp mediated MDR expression.	Shows potent P-gp inhibitory effect on breast cancer cell line (MCF-7/ADR) and enhances daunomycin uptake.	Not found.	[246]

## References

[1] Ling V., Thompson L.H. (1974). Reduced permeability in CHO cells as a mechanism of resistance to colchicine. J. Cell. Physiol..

[2] Linton K.J., Higgins C.F. (2007). Structure and function of ABC transporters: The ATP switch provides flexible control. Eur. J. Physiol..

[3] Ikegawa T., Ushigome F., Koyabu N., Morimoto S., Shoyama Y., Naito M., Tsuruo T., Ohtani H., Sawada Y. (2000). Inhibition of P-glycoprotein by orange juice components, polymethoxyflavones in adriamycin-resistant human myelogenous leukemia (K562/ADM) cells. Cancer Lett..

[4] Nabekura T., Hiroi T., Kawasaki T., Uwai Y. (2015). Effects of natural nuclear factor-kappa β inhibitors on anticancer drug efflux transporter human P-glycoprotein. Biomed. Pharmacother..

[5] Bansal T., Jaggi M., Khar R., Talegaonkar S. (2009). Emerging significance of flavonoids as P-glycoprotein inhibitors in cancer chemotherapy. J. Pharm. Pharm. Sci..

[6] Yoshida N., Koizumi M., Adachi I., Kawakami J. (2006). Inhibition of P-glycoprotein-mediated transport by terpenoids contained in herbal medicines and natural products. Food Chem. Toxicol..

[7] Lopez D., Martinez-Luis S. (2014). Marine natural products with P-glycoprotein inhibitor properties. Mar. Drugs.

[8] Fischer V., Einolf H., Cohen D. (2005). Efflux transporters and their clinical relevance. Mini Rev. Med. Chem..

[9] Van Helvoort A., Smith A.J., Sprong H., Fritzsche I., Schinkel A.H., Borst P., van Meer G. (1996). Mdr1 P-glycoprotein is a lipid translocase of broad specificity, while MDR3 P-glycoprotein specifically translocates phosphatidylcholine. Cell.

[10] Smith A.J., van Helvoort A., van Meer G., Szabó K., Welker E., Szakács G., Váradi A., Sarkadi B., Borst P. (2000). MDR3 P-glycoprotein, a phosphatidylcholine translocase, transports several cytotoxic drugs and directly interacts with drugs as judged by interference with nucleotide trapping. J. Biol. Chem..

[11] Bellamy W.T., Dalton W.S. (1993). Multidrug resistance in the laboratory and clinic. Adv. Clin. Chem..

[12] Georges E., Bradley G., Gariepy J., Ling V. (1990). Detection of P-glycoprotein isoforms by gene-specific monoclonal antibodies. Proc. Natl. Acad. Sci. USA.

[13] Lamy T., Drenou B., Grulois I., Fardel O., Jacquelinet C., Goasguen J., Dauriac C., Amiot L., Bernard M., Fauchet R. (1995). Multi-drug resistance (MDR) activity in acute leukemia determined by rhodamine 123 efflux assay. Leukemia.

[14] Kramer R., Weber T., Morse B., Arceci R., Staniunas R., Steele G.J., Summerhayes I. (1993). Constitutive expression of multidrug resistance in human colorectal tumours and cell lines. Br. J. Cancer.

[15] Bourhis J., Bénard J., Hartmann O., Boccon-Gibod L., Lemerle J., Riou G. (1989). Correlation of mdr1 gene expression with chemotherapy in neuroblastoma. J. Natl. Cancer Inst..

[16] List A.F., Spier C.M., Cline A., Doll D.C., Garewal H., Morgan R., Sandberg A.A. (1991). Expression of the multidrug resistance gene product (P-glycoprotein) in myelodysplasia is associated with a stem cell phenotype. Br. J. Haematol..

[17] Drenou B., Fardel O., Amiot L., Fauchet R. (1993). Detection of p glycoprotein activity on normal and leukemic cd34+ cells. Leuk. Res..

[18] Lai S.-L., Goldstein L.J., Gottesman M.M., Pastan I., Tsai C.-M., Johnson B.E., Mulshine J.L., Ihde D.C., Kayser K., Gazdar A.F. (1989). mdr1 gene expression in lung cancer. J. Natl. Cancer Inst..

[19] Moscow J.A., Fairchild C.R., Madden M.J., Ransom D.T., Wieand H.S., O’Brien E.E., Poplack D.G., Cossman J., Myers C.E., Cowan K.H. (1989). Expression of anionic glutathione-s-transferase and P-glycoprotein genes in human tissues and tumors. Cancer Res..

[20] Marie J.-P., Zittoun R., Sikic B.I. (1991). Multidrug resistance (mdr1) gene expression in adult acute leukemias: Correlations with treatment outcome and in vitro drug sensitivity. Blood.

[21] Cheng A.-L., Su I.-J., Chen Y.-C., Lee T.-C., Wang C.-H. (1993). Expression of P-glycoprotein and glutathione-s-transferase in recurrent lymphomas: The possible role of Epstein-Barr virus, immunophenotypes, and other predisposing factors. J. Clin. Oncol..

[22] Varma M.V., Ashokraj Y., Dey C.S., Panchagnula R. (2003). P-glycoprotein inhibitors and their screening: A perspective from bioavailability enhancement. Pharmacol. Res..

[23] Thiebaut F., Tsuruo T., Hamada H., Gottesman M.M., Pastan I., Willingham M.C. (1987). Cellular localization of the multidrug-resistance gene product P-glycoprotein in normal human tissues. Proc. Natl. Acad. Sci. USA.

[24] Kobayashi Y., Yamashiro T., Nagatake H., Yamamoto T., Watanabe N., Tanaka H., Shigenobu K., Tsuruo T. (1994). Expression and function of multidrug resistance P-glycoprotein in a cultured natural killer cell-rich population revealed by mrk16 monoclonal antibody and AHC-52. Biochem. Pharmacol..

[25] Chaudhary P.M., Roninson I.B. (1991). Expression and activity of P-glycoprotein, a multidrug efflux pump, in human hematopoietic stem cells. Cell.

[26] Bendayan R., Ronaldson P.T., Gingras D., Bendayan M. (2006). In situ localization of P-glycoprotein (ABCB1) in human and rat brain. J. Histochem. Cytochem..

[27] Chang G. (2003). Multidrug resistance ABC transporters. FEBS Lett..

[28] Lage H. (2003). ABC-transporters: Implications on drug resistance from microorganisms to human cancers. Int. J. Antimicrob. Agents.

[29] Higgins C.F. (2001). ABC transporters: Physiology, structure and mechanism—An overview. Res. Microbiol..

[30] Manson J.M., Keis S., Smith J.M., Cook G.M. (2004). Acquired bacitracin resistance in *Enterococcus faecalis* is mediated by an ABC transporter and a novel regulatory protein, bcrr. Antimicrob. Agents Chemother..

[31] Lynch A.S. (2006). Efflux systems in bacterial pathogens: An opportunity for therapeutic intervention? An industry view. Biochem. Pharmacol..

[32] Pagès J.-M., Masi M., Barbe J. (2005). Inhibitors of efflux pumps in gram-negative bacteria. Trends Mol. Med..

[33] Saier M.H., Beatty J.T., Goffeau A., Harley K.T., Heijne W., Huang S.-C., Jack D.L., Jahn P., Lew K., Liu J. (1999). The major facilitator superfamily. J. Mol. Microbiol. Biotechnol..

[34] Bodor M., Kelley E.J., Ho R.J. (2005). Characterization of the human mdr1 gene. AAPS J..

[35] Loo T.W., Clarke D.M. (2005). Do drug substrates enter the common drug-binding pocket of P-glycoprotein through “gates”?. Biochem. Biophys. Res. Commun..

[36] Hrycyna C.A., Airan L.E., Germann U.A., Ambudkar S.V., Pastan I., Gottesman M.M. (1998). Structural flexibility of the linker region of human P-glycoprotein permits ATP hydrolysis and drug transport. Biochemistry.

[37] Hung L.-W., Wang I.X., Nikaido K., Liu P.-Q., Ames G.F.-L., Kim S.-H. (1998). Crystal structure of the ATP-binding subunit of an ABC transporter. Nature.

[38] Booth C.L., Pulaski L., Gottesman M.M., Pastan I. (2000). Analysis of the properties of the n-terminal nucleotide-binding domain of human P-glycoprotein. Biochemistry.

[39] Tombline G., Bartholomew L., Gimi K., Tyndall G.A., Senior A.E. (2004). Synergy between conserved abc signature ser residues in P-glycoprotein catalysis. J. Biol. Chem..

[40] Yoshida N., Takagi A., Kitazawa H., Kawakami J., Adachi I. (2005). Inhibition of P-glycoprotein-mediated transport by extracts of and monoterpenoids contained in *zanthoxyli fructus*. Toxicol. Appl. Pharmacol..

[41] Ambudkar S.V., Dey S., Hrycyna C.A., Ramachandra M., Pastan I., Gottesman M.M. (1999). Biochemical, cellular, and pharmacological aspects of the multidrug transporter 1. Annu. Rev. Pharmacol. Toxicol..

[42] Ueda K., Taguchi Y., Morishima M. (1997). How does P-glycoprotein recognize its substrates?. Semin. Cancer Biol..

[43] Loo T.W., Bartlett M.C., Clarke D.M. (2005). The dileucine motif at the cooh terminus of human multidrug resistance P-glycoprotein is important for folding but not activity. J. Biol. Chem..

[44] Loo T.W., Bartlett M.C., Clarke D.M. (2004). Disulfide cross-linking analysis shows that transmembrane segments 5 and 8 of human P-glycoprotein are close together on the cytoplasmic side of the membrane. J. Biol. Chem..

[45] Loo T.W., Bartlett M.C., Clarke D.M. (2004). Val133 and cys137 in transmembrane segment 2 are close to ARG935 and GLY939 in transmembrane segment 11 of human P-glycoprotein. J. Biol. Chem..

[46] Loo T., Clarke D. (2005). Recent progress in understanding the mechanism of P-glycoprotein-mediated drug efflux. J. Membr. Biol..

[47] Hennessy M., Spiers J. (2007). A primer on the mechanics of P-glycoprotein the multidrug transporter. Pharmacol. Res..

[48] Higgins C.F., Gottesman M.M. (1992). Is the multidrug transporter a flippase?. Trends Biochem. Sci..

[49] Srivalli K.M.R., Lakshmi P. (2012). Overview of P-glycoprotein inhibitors: A rational outlook. Braz. J. Pharm. Sci..

[50] Lomovskaya O., Bostian K.A. (2006). Practical applications and feasibility of efflux pump inhibitors in the clinic—A vision for applied use. Biochem. Pharmacol..

[51] Lomovskaya O., Zgurskaya H.I., Totrov M., Watkins W.J. (2007). Waltzing transporters and ‘the dance macabre ‘between humans and bacteria. Nat. Rev. Drug Discov..

[52] Kuppens I.E., Witteveen E.O., Jewell R.C., Radema S.A., Paul E.M., Mangum S.G., Beijnen J.H., Voest E.E., Schellens J.H. (2007). A phase I, randomized, open-label, parallel-cohort, dose-finding study of elacridar (gf120918) and oral topotecan in cancer patients. Clin. Cancer Res..

[53] Pusztai L., Wagner P., Ibrahim N., Rivera E., Theriault R., Booser D., Symmans F.W., Wong F., Blumenschein G., Fleming D.R. (2005). Phase ii study of tariquidar, a selective P-glycoprotein inhibitor, in patients with chemotherapy-resistant, advanced breast carcinoma. Cancer.

[54] Krishna R., Mayer L.D. (2000). Multidrug resistance (MDR) in cancer: Mechanisms, reversal using modulators of mdr and the role of MDR modulators in influencing the pharmacokinetics of anticancer drugs. Eur. J. Pharm. Sci..

[55] Binkhathlan Z., Lavasanifar A. (2013). P-glycoprotein inhibition as a therapeutic approach for overcoming multidrug resistance in cancer: Current status and future perspectives. Curr. Cancer Drug Targets.

[56] Ieiri I. (2012). Functional significance of genetic polymorphisms in P-glycoprotein (MDR1, ABCB1) and breast cancer resistance protein (BCRP, ABCG2). Drug Metab. Pharmacokinet..

[57] Piao Y., Shin S.C., Choi J.S. (2008). Effects of oral kaempferol on the pharmacokinetics of tamoxifen and one of its metabolites, 4-hydroxytamoxifen, after oral administration of tamoxifen to rats. Biopharm. Drug Dispos..

[58] Chavez M.L., Jordan M.A., Chavez P.I. (2006). Evidence-based drug–herbal interactions. Life Sci..

[59] Zhou S.-F., Zhou Z.-W., Li C.-G., Chen X., Yu X., Xue C.C., Herington A. (2007). Identification of drugs that interact with herbs in drug development. Drug Discov. Today.

[60] Foster B.C., Foster M.S., Vandenhoek S., Krantis A., Budzinski J.W., Arnason J.T., Gallicano K.D., Choudri S. (2001). An in vitro evaluation of human cytochrome p450 3a4 and P-glycoprotein inhibition by garlic. J. Pharm. Pharm. Sci..

[61] Ignacimuthu S., Shanmugam N. (2010). Antimycobacterial activity of two natural alkaloids, vasicine acetate and 2-acetyl benzylamine, isolated from Indian shrub *Adhatoda vasica* Ness. leaves. J. Biosci..

[62] O’Donnell G., Gibbons S. (2007). Antibacterial activity of two canthin-6-one alkaloids from *Allium neapolitanum*. Phytother. Res..

[63] Tian H., Pan O. (1997). Modulation of multidrug resistance by three bisbenzyl-isoquinolines in comparison with verapamil. Acta Pharmacol. Sin..

[64] Chou T.-C., Depew K.M., Zheng Y.-H., Safer M.L., Chan D., Helfrich B., Zatorska D., Zatorski A., Bornmann W., Danishefsky S.J. (1998). Reversal of anticancer multidrug resistance by the ardeemins. Proc. Natl. Acad. Sci. USA.

[65] Rabindran S.K., Ross D.D., Doyle L.A., Yang W., Greenberger L.M. (2000). Fumitremorgin c reverses multidrug resistance in cells transfected with the breast cancer resistance protein. Cancer Res..

[66] Nakatsu S., Kondo S., Kondo Y., Yin D., Peterson J.W., Kaakaji R., Morimura T., Kikuchi H., Takeuchi J., Barnett G.H. (1997). Induction of apoptosis in multi-drug resistant (mdr) human glioblastoma cells by sn-38, a metabolite of the camptothecin derivative cpt-11. Cancer Chemother. Pharmacol..

[67] Gupta M., Fan S., Zhan Q., Kohn K.W., O’Connor P.M., Pommier Y. (1997). Inactivation of p53 increases the cytotoxicity of camptothecin in human colon HCT116 and breast MCF-7 cancer cells. Clin. Cancer Res..

[68] Okura T., Ibe M., Umegaki K., Shinozuka K., Yamada S. (2010). Effects of dietary ingredients on function and expression of P-glycoprotein in human intestinal epithelial cells. Biol. Pharm. Bull..

[69] He L., Liu G.-Q. (2002). Effects of various principles from Chinese herbal medicine on rhodamine123 accumulation in brain capillary endothelial cells. Acta Pharmacol. Sin..

[70] Lee S.Y., Rhee Y.H., Jeong S.J., Lee H.J., Lee H.J., Jung M.H., Kim S.H., Lee E.O., Ahn K.S., Ahn K.S. (2011). Hydrocinchonine, cinchonine, and quinidine potentiate paclitaxel-induced cytotoxicity and apoptosis via multidrug resistance reversal in MES-SA/DX5 uterine sarcoma cells. Environ. Toxicol..

[71] Solary E., Mannone L., Moreau D., Caillot D., Casasnovas R., Guy H., Grandjean M., Wolf J., Andre F., Fenaux P. (2000). Phase I study of cinchonine, a multidrug resistance reversing agent, combined with the chvp regimen in relapsed and refractory lymphoproliferative syndromes. Leukemia.

[72] Yasuda K., Lan L.B., Sanglard D., Furuya K., Schuetz J.D., Schuetz E.G. (2002). Interaction of cytochrome P450 A inhibitors with P-glycoprotein. J. Pharmacol. Exp. Ther..

[73] Gibbons S. (2008). Phytochemicals for bacterial resistance-strengths, weaknesses and opportunities. Planta Med..

[74] Min Y.D., Yang M.C., Lee K.H., Kim K.R., Choi S.U., Lee K.R. (2006). Protoberberine alkaloids and their reversal activity of P-gp expressed multidrug resistance (MDR) from the rhizome of *Coptis japonica* Makino. Arch. Pharm. Res..

[75] Lei Y., Tan J., Wink M., Ma Y., Li N., Su G. (2013). An isoquinoline alkaloid from the Chinese herbal plant *Corydalis yanhusuo* wt wang inhibits P-glycoprotein and multidrug resistance-associate protein 1. Food Chem..

[76] Kang H., Jang S.-W., Pak J.H., Shim S. (2015). Glaucine inhibits breast cancer cell migration and invasion by inhibiting mmp-9 gene expression through the suppression of NF-κB activation. Mol. Cell. Biochem..

[77] Kim E.-H., Min H.-Y., Chung H.-J., Song J., Park H.-J., Kim S., Lee S.K. (2012). Anti-proliferative activity and suppression of P-glycoprotein by (−)-antofine, a natural phenanthroindolizidine alkaloid, in paclitaxel-resistant human lung cancer cells. Food Chem. Toxicol..

[78] Wright A.E., Forleo D.A., Gunawardana G.P., Gunasekera S.P., Koehn F.E., McConnel O. (1990). Antitumor tetrahydroisoquinoline alkaloids from the colonial ascidian *Ecteinascidia turbinata*. J. Org. Chem..

[79] Kanzaki A., Takebayashi Y., Ren X.-Q., Miyashita H., Mori S., Akiyama S.-I., Pommier Y. (2002). Overcoming multidrug drug resistance in P-glycoprotein/mdr1-overexpressing cell lines by ecteinascidin 743. Mol. Cancer Ther..

[80] Mi Q., Cui B., Silva G.L., Lantvit D., Lim E., Chai H., You M., Hollingshead M.G., Mayo J.G., Kinghorn A.D. (2001). Pervilleine A, a novel tropane alkaloid that reverses the multidrug-resistance phenotype. Cancer Res..

[81] Smith C.D., Zilfou J.T., Stratmann K., Patterson G., Moore R.E. (1995). Welwitindolinone analogues that reverse P-glycoprotein-mediated multiple drug resistance. Mol. Pharmacol..

[82] Severina I.I., Muntyan M.S., Lewis K., Skulachev V.P. (2001). Transfer of cationic antibacterial agents berberine, palmatine, and benzalkonium through bimolecular planar phospholipid film and *Staphylococcus aureus* membrane. IUBMB Life.

[83] Maurya A., Dwivedi G.R., Darokar M.P., Srivastava S.K. (2013). Antibacterial and synergy of clavine alkaloid lysergol and its derivatives against nalidixic acid-resistant *Escherichia coli*. Chem. Biol. Drug Dis..

[84] Rho M.-C., Toyoshima M., Hayashi M., Koyano T., Subramaniam G., Kam T.-S., Komiyama K. (1999). Reversal of multidrug resistance by kopsiflorine isolated from *Kopsia dasyrachis*. Planta Med..

[85] Kam T.-S., Subramaniam G., Sim K.-M., Yoganathan K., Koyano T., Toyoshima M., Rho M.-C., Hayashi M., Komiyama K. (1998). Reversal of multidrug resistance (MDR) by aspidofractinine-type indole alkaloids. Bioorg. Med. Chem. Lett..

[86] Quesada A., Grávalos M.G., Puentes J.F. (1996). Polyaromatic alkaloids from marine invertebrates as cytotoxic compounds and inhibitors of multidrug resistance caused by P-glycoprotein. Br. J. Cancer.

[87] Williams A.B., Jacobs R.S. (1993). A marine natural product, patellamide d, reverses multidrug resistance in a human leukemic cell line. Cancer Lett..

[88] Fu X., Do T., Schmitz F.J., Andrusevich V., Engel M.H. (1998). New cyclic peptides from the ascidian *Lissoclinum patella*. J. Nat. Prod..

[89] García-Reynaga P., VanNieuwenhze M.S. (2008). A new total synthesis of patellamide A. Org. Lett..

[90] Ma Y., Wink M. (2008). Lobeline, a piperidine alkaloid from *Lobelia* can reverse P-gp dependent multidrug resistance in tumor cells. Phytomedicine.

[91] Cherigo L., Lopez D., Martinez-Luis S. (2015). Marine natural products as breast cancer resistance protein inhibitors. Mar. Drugs.

[92] Robey R.W., Shukla S., Steadman K., Obrzut T., Finley E.M., Ambudkar S.V., Bates S.E. (2007). Inhibition of ABCG2-mediated transport by protein kinase inhibitors with a bisindolylmaleimide or indolocarbazole structure. Mol. Cancer Ther..

[93] Michalet S., Cartier G., David B., Mariotte A.-M., Dijoux-franca M.-G., Kaatz G.W., Stavri M., Gibbons S. (2007). *N*-caffeoylphenalkylamide derivatives as bacterial efflux pump inhibitors. Bioorg. Med. Chem. Lett..

[94] Mohtar M., Johari S.A., Li A.R., Isa M.M., Mustafa S., Ali A.M., Basri D.F. (2009). Inhibitory and resistance-modifying potential of plant-based alkaloids against methicillin-resistant *Staphylococcus aureus* (mrsa). Curr. Microbiol..

[95] Ma Y., Wink M. (2010). The beta-carboline alkaloid harmine inhibits bcrp and can reverse resistance to the anticancer drugs mitoxantrone and camptothecin in breast cancer cells. Phytother. Res..

[96] Eid S.Y., El-Readi M.Z., Eldin E.E.M.N., Fatani S.H., Wink M. (2013). Influence of combinations of digitonin with selected phenolics, terpenoids, and alkaloids on the expression and activity of P-glycoprotein in leukaemia and colon cancer cells. Phytomedicine.

[97] You M., Ma X., Mukherjee R., Farnsworth N.R., Cordell G.A., Kinghorn A.D., Pezzuto J.M. (1994). Indole alkaloids from *peschiera laeta* that enhance vinblastine-mediated cytotoxicity with multidrug-resistant cells. J. Nat. Prod..

[98] Meschini S., Marra M., Calcabrini A., Federici E., Galeffi C., Arancia G. (2003). Voacamine, a bisindolic alkaloid from *peschiera fuchsiaefolia*, enhances the cytotoxic effect of doxorubicin on multidrug-resistant tumor cells. Int. J. Oncol..

[99] Meschini S., Marra M., Condello M., Calcabrini A., Federici E., Dupuis M., Cianfriglia M., Arancia G. (2005). Voacamine, an alkaloid extracted from *peschiera fuchsiaefolia*, inhibits P-glycoprotein action in multidrug-resistant tumor cells. Int. J. Oncol..

[100] Min Y.D., Kwon H.C., Yang M.C., Lee K.H., Choi S.U., Lee K.R. (2007). Isolation of limonoids and alkaloids from *Phellodendron amurense* and their multidrug resistance (MDR) reversal activity. Arch. Pharm. Res..

[101] Han Y., Tan T.M.C., Lim L.-Y. (2008). In vitro and in vivo evaluation of the effects of piperine on P-gp function and expression. Toxicol. Appl. Pharmacol..

[102] Sharma S., Kumar M., Sharma S., Nargotra A., Koul S., Khan I.A. (2010). Piperine as an inhibitor of rv1258c, a putative multidrug efflux pump of mycobacterium tuberculosis. J. Antimicrob. Chemother..

[103] Stavri M., Piddock L.J., Gibbons S. (2007). Bacterial efflux pump inhibitors from natural sources. J. Antimicrob. Chemother..

[104] Abdelfatah S.A., Efferth T. (2015). Cytotoxicity of the indole alkaloid reserpine from *Rauwolfia serpentina* against drug-resistant tumor cells. Phytomedicine.

[105] Eliason J.F., Ramuz H., Yoshikubo T., Ishikawa T., Yamamoto T., Tsuruo T. (1995). Novel dithiane analogues of tiapamil with high activity to overcome multidrug resistance in vitro. Biochem. Pharmacol..

[106] Gibbons S., Udo E. (2000). The effect of reserpine, a modulator of multidrug efflux pumps, on the in vitro activity of tetracycline against clinical isolates of methicillin resistant *Staphylococcus aureus* (MRSA) possessing the tet (k) determinant. Phytother. Res..

[107] Rethy B., Hohmann J., Minorics R., Varga A., Ocsovszki I., Molnar J., Juhász K., Falkay G., Zupko I. (2008). Antitumour properties of acridone alkaloids on a murine lymphoma cell line. Anticancer Res..

[108] Ding Z., Tang S.-C., Weerasinghe P., Yang X., Pater A., Liepins A. (2002). The alkaloid sanguinarine is effective against multidrug resistance in human cervical cells via bimodal cell death. Biochem. Pharmacol..

[109] Weerasinghe P., Hallock S., Tang S.-C., Trump B., Liepins A. (2006). Sanguinarine overcomes P-glycoprotein-mediated multidrug-resistance via induction of apoptosis and oncosis in CEM-VLB 1000 cells. Exp. Toxicol. Pathol..

[110] Min Y.D., Choi S.U., Lee K.R. (2006). Aporphine alkaloids and their reversal activity of multidrug resistance (MDR) from the stems and rhizomes of *sinomenium acutum*. Arch. Pharm. Res..

[111] Lavie Y., Harel-Orbital T., Gaffield W., Liscovitch M. (2001). Inhibitory effect of steroidal alkaloids on drug transport and multidrug resistance in human cancer cells. Anticancer Res..

[112] Ding Y., Xie X., Zhao J., Yang P. (2004). Reversal of adriamycin resistance by matrine in leukemia multidrug resistance cell line k562/adm. J. Dalian Med. Univ..

[113] Li X., Zhang S., Zheng S. (2001). Cellular biological effects of matrine on k562 and k562/vin cells. Chin. J. Pathophysiol..

[114] Chanmahasathien W., Ampasavate C., Greger H., Limtrakul P. (2011). Stemona alkaloids, from traditional Thai medicine, increase chemosensitivity via P-glycoprotein-mediated multidrug resistance. Phytomedicine.

[115] Ikeda R., Che X.F., Yamaguchi T., Ushiyama M., Zheng C.L., Okumura H., Takeda Y., Shibayama Y., Nakamura K., Jeung H.C. (2005). Cepharanthine potently enhances the sensitivity of anticancer agents in k562 cells. Cancer Sci..

[116] Sun Y.F., Wink M. (2014). Tetrandrine and fangchinoline, bisbenzylisoquinoline alkaloids from stephania tetrandra can reverse multidrug resistance by inhibiting P-glycoprotein activity in multidrug resistant human cancer cells. Phytomedicine.

[117] Wang F.-P., Wang L., Yang J.-S., Nomura M., Miyamoto K.-I. (2005). Reversal of P-glycoprotein-dependent resistance to vinblastine by newly synthesized bisbenzylisoquinoline alkaloids in mouse leukemia p388 cells. Biol. Pharm. Bull..

[118] Choi S.-U., Park S.-H., Kim K.-H., Choi E.-J., Kim S., Park W.-K., Zhang Y.-H., Kim H.-S., Jung N.-P., Lee C.-O. (1998). The bis benzylisoquinoline alkaloids, tetrandine and fangchinoline, enhance the cytotoxicity of multidrug resistance-related drugs via modulation of P-glycoprotein. Anticancer Drugs.

[119] Kam T.-S., Sim K.-M., Pang H.-S., Koyano T., Hayashi M., Komiyama K. (2004). Cytotoxic effects and reversal of multidrug resistance by ibogan and related indole alkaloids. Bioorg. Med. Chem. Lett..

[120] Tournier N., Chevillard L., Megarbane B., Pirnay S., Scherrmann J.-M., Decleves X. (2010). Interaction of drugs of abuse and maintenance treatments with human P-glycoprotein (ABCB1) and breast cancer resistance protein (ABCG2). Int. J. Neuropsychopharmacol..

[121] Piddock L.J., Garvey M.I., Rahman M.M., Gibbons S. (2010). Natural and synthetic compounds such as trimethoprim behave as inhibitors of efflux in gram-negative bacteria. J. Antimicrob. Chemother..

[122] Ivanova A., Serly J., Christov V., Stamboliyska B., Molnar J. (2011). Alkaloids derived from genus *Veratrum* and *Peganum* of mongolian origin as multidrug resistance inhibitors of cancer cells. Fitoterapia.

[123] Cabral V., Luo X., Junqueira E., Costa S.S., Mulhovo S., Duarte A., Couto I., Viveiros M., Ferreira M.-J.U. (2015). Enhancing activity of antibiotics against *staphylococcus aureus*: *Zanthoxylum capense* constituents and derivatives. Phytomedicine.

[124] Gibbons S., Leimkugel J., Oluwatuyi M., Heinrich M. (2003). Activity of *Zanthoxylum clava-herculis* extracts against multi-drug resistant methicillin-resistant *Staphylococcus aureus* (MDR-MRSA). Phytother. Res..

[125] Gyemant N., Tanaka M., Antus S., Hohmann J., Csuka O., Mandoky L., Molnar J. (2005). In vitro search for synergy between flavonoids and epirubicin on multidrug-resistant cancer cells. In Vivo.

[126] Ye J., Zheng Y., Liu D. (2009). Reversal effect and its mechanism of ampelopsin on multidrug resistance in K562/ADR cells. Zhongguo Zhong Yao Za Zhi.

[127] Fiamegos Y.C., Kastritis P.L., Exarchou V., Han H., Bonvin A.M., Vervoort J., Lewis K., Hamblin M.R., Tegos G.P. (2011). Antimicrobial and efflux pump inhibitory activity of caffeoylquinic acids from *Artemisia absinthium* against gram-positive pathogenic bacteria. PLoS ONE.

[128] Stermitz F.R., Scriven L.N., Tegos G., Lewis K. (2002). Two flavonols from *Artemisa annua* which potentiate the activity of berberine and norfloxacin against a resistant strain of *Staphylococcus aureus*. Planta Med..

[129] Liu K.C., Yang S.-L., Roberts M.F., Elford B.C., Phillipson J.D. (1992). Antimalarial activity of *Artemisia annua* flavonoids from whole plants and cell cultures. Plant Cell Rep..

[130] Jodoin J., Demeule M., BeÂliveay R. (2002). Inhibition of the multidrug resistance P-glycoprotein activity by green tea polyphenols. Biochim. Biophys. Acta.

[131] Knop J., Misaka S., Singer K., Hoier E., Müller F., Glaeser H., König J., Fromm M.F. (2015). Inhibitory effects of green tea and (–)-epigallocatechin gallate on transport by OATP1B1, OATP1B3, OCT1, OCT2, MATE1, MATE2-K and P-glycoprotein. PLoS ONE.

[132] Mossa J.S., El-Feraly F.S., Muhammad I. (2004). Antimycobacterial constituents from *Juniperus procera*, *Ferula communis* and *Plumbago zeylanica* and their in vitro synergistic activity with isonicotinic acid hydrazide. Phytother. Res..

[133] Rodgers E.H., Grant M.H. (1998). The effect of the flavonoids, quercetin, myricetin and epicatechin on the growth and enzyme activities of MCF7 human breast cancer cells. Chem. Biol. Interact..

[134] Ofer M., Wolffram S., Koggel A., Spahn-Langguth H., Langguth P. (2005). Modulation of drug transport by selected flavonoids: Involvement of P-gp and OCT?. Eur. J. Pharm. Sci..

[135] Cooray H.C., Janvilisri T., van Veen H.W., Hladky S.B., Barrand M.A. (2004). Interaction of the breast cancer resistance protein with plant polyphenols. Biochem. Biophys. Res. Commun..

[136] Van Zanden J.J., Wortelboer H.M., Bijlsma S., Punt A., Usta M., Bladeren P.J., Rietjens I.M., Cnubben N.H. (2005). Quantitative structure activity relationship studies on the flavonoid mediated inhibition of multidrug resistance proteins 1 and 2. Biochem. Pharmacol..

[137] Zhang S., Morris M.E. (2003). Effects of the flavonoids biochanin a, morin, phloretin, and silymarin on P-glycoprotein-mediated transport. J. Pharmacol. Exp. Ther..

[138] Zhang S., Yang X., Morris M.E. (2004). Flavonoids are inhibitors of breast cancer resistance protein (ABCG2)-mediated transport. Mol. Pharmacol..

[139] Lechner D., Gibbons S., Bucar F. (2008). Plant phenolic compounds as ethidium bromide efflux inhibitors in *Mycobacterium smegmatis*. J. Antimicrob. Chemother..

[140] Mertens-Talcott S.U., De Castro W.V., Manthey J.A., Derendorf H., Butterweck V. (2007). Polymethoxylated flavones and other phenolic derivates from citrus in their inhibitory effects on P-glycoprotein-mediated transport of talinolol in Caco-2 cells. J. Agric. Food Chem..

[141] Choi C.-H., Sun K.-H., An C.-S., Yoo J.-C., Hahm K.-S., Lee I.-H., Sohng J.-K., Kim Y.-C. (2002). Reversal of P-glycoprotein-mediated multidrug resistance by 5,6,7,3′,4′-pentamethoxyflavone (sinensetin). Biochem. Biophys. Res. Commun..

[142] Romiti N., Tramonti G., Donati A., Chieli E. (2004). Effects of grapefruit juice on the multidrug transporter P-glycoprotein in the human proximal tubular cell line hk-2. Life Sci..

[143] Surya Sandeep M., Sridhar V., Puneeth Y., Ravindra Babu P., Naveen Babu K. (2014). Enhanced oral bioavailability of felodipine by naringenin in wistar rats and inhibition of P-glycoprotein in everted rat gut sacs in vitro. Drug Dev. Ind. Pharm..

[144] Khantamat O., Chaiwangyen W., Limtrakul P.-N. (2004). Screening of flavonoids for their potential inhibitory effect on P-glycoprotein activity in human cervical carcinoma kb cells. Chiang Mai Med. Bull..

[145] De Castro W.V., Mertens-Talcott S., Derendorf H., Butterweck V. (2007). Grapefruit juice–Drug interactions: Grapefruit juice and its components inhibit P-glycoprotein (ABCB1) mediated transport of talinolol in Caco-2 cells. J. Pharm. Sci..

[146] De Castro W.V., Mertens-Talcott S., Derendorf H., Butterweck V. (2008). Effect of grapefruit juice, naringin, naringenin, and bergamottin on the intestinal carrier-mediated transport of talinolol in rats. J. Agric. Food Chem..

[147] Mitsunaga Y., Takanaga H., Matsuo H., Naito M., Tsuruo T., Ohtani H., Sawada Y. (2000). Effect of bioflavonoids on vincristine transport across blood–brain barrier. Eur. J. Pharmacol..

[148] El-Readi M.Z., Hamdan D., Farrag N., El-Shazly A., Wink M. (2010). Inhibition of P-glycoprotein activity by limonin and other secondary metabolites from citrus species in human colon and leukaemia cell lines. Eur. J. Pharmacol..

[149] Najar I., Sachin B., Sharma S., Satti N., Suri K., Johri R. (2010). Modulation of P-glycoprotein atpase activity by some phytoconstituents. Phytother. Res..

[150] Rameshkumar K., Alan Sheeja D., Nair M.S., George V. (2015). *Curcuma ecalcarata*–New natural source of pinocembrin and piperitenone. Nat. Prod. Res..

[151] Yang Z.-H., Sun X., Qi Y., Mei C., Sun X.-B., Du G.-H. (2012). Uptake characteristics of pinocembrin and its effect on P-glycoprotein at the blood–brain barrier in in vitro cell experiments. J. Asian Nat. Prod. Res..

[152] Limtrakul P., Anuchapreeda S., Buddhasukh D. (2004). Modulation of human multidrug-resistance MDR-1 gene by natural curcuminoids. BMC Cancer.

[153] Lu J.-J., Cai Y.-J., Ding J. (2012). The short-time treatment with curcumin sufficiently decreases cell viability, induces apoptosis and copper enhances these effects in multidrug-resistant k562/a02 cells. Mol. Cell. Biochem..

[154] Belofsky G., Carreno R., Lewis K., Ball A., Casadei G., Tegos G.P. (2006). Metabolites of the “smoke tree”, *Dalea spinosa*, potentiate antibiotic activity against multidrug-resistant *Staphylococcus aureus*. J. Nat. Prod..

[155] Kuete V., Ngameni B., Tangmouo J.G., Bolla J.-M., Alibert-Franco S., Ngadjui B.T., Pagès J.-M. (2010). Efflux pumps are involved in the defense of gram-negative bacteria against the natural products isobavachalcone and diospyrone. Antimicrob. Agents Chemother..

[156] Lechner D., Gibbons S., Jachak S., Srivastava A., Bucar F. In Curcuminoids as Efflux Pump Inhibitors (EPIS) in Mycobacterium Smegmatis mc2155. Proceedings of the 7th Joint Meeting of GA, AFERP, ASP, PSI & SIF.

[157] Saczko J., Kulbacka J., Chwilkowska A., Pola A., Lugowski M., Marcinkowska A., Malarska A., Banas T. (2007). Cytosolic superoxide dismutase activity after photodynamic therapy, intracellular distribution of photofrin ii and hypericin, and P-glycoprotein localization in human colon adenocarcinoma. Folia Histochem. Cytobiol..

[158] Eagling V., Profit L., Back D. (1999). Inhibition of the CYP3A4-mediated metabolism and P-glycoprotein-mediated transport of the hiv-1 protease inhibitor saquinavir by grapefruit juice components. Br. J. Clin. Pharmacol..

[159] Sun L., Chen W., Qu L., Wu J., Si J. (2013). Icaritin reverses multidrug resistance of HepG2/ADR human hepatoma cells via downregulation of mdr1 and p‑glycoprotein expression. Mol. Med. Rep..

[160] Falcão-Silva V.S., Silva D.A., Souza M.d.F.V., Siqueira-Junior J.P. (2009). Modulation of drug resistance in *staphylococcus aureus* by a kaempferol glycoside from *Herissantia tiubae* (Malvaceae). Phytother. Res..

[161] Yarla N., Ganapaty S. (2013). Bioactive flavonoids as ABC transporters inhibitors for reversion of multidrug resistance in cancer. J. Mar. Sci. Res. Dev..

[162] Wesołowska O., Wiśniewski J., Środa K., Krawczenko A., Bielawska-Pohl A., Paprocka M., Duś D., Michalak K. (2010). 8-prenylnaringenin is an inhibitor of multidrug resistance-associated transporters, P-glycoprotein and MRP1. Eur. J. Pharmacol..

[163] An G., Wu F., Morris M.E. (2011). 5,7-dimethoxyflavone and multiple flavonoids in combination alter the ABCG2-mediated tissue distribution of mitoxantrone in mice. Pharm. Res..

[164] Jia H., Yang Q., Wang T., Cao Y., Jiang Q.-Y., Sun H.-W., Hou M.-X., Yang Y.-P., Feng F. (2016). Rhamnetin induces sensitization of hepatocellular carcinoma cells to a small molecular kinase inhibitor or chemotherapeutic agents. Biochim. Biophys. Acta (BBA)-Gen. Subj..

[165] Guo X.L., Leng P., Yang Y., Yu L.G., Lou H.X. (2008). Plagiochin E, a botanic-derived phenolic compound, reverses fungal resistance to fluconazole relating to the efflux pump. J. Appl. Microbiol..

[166] Kale M.S., Laddha K. (2012). Isolation, characterization and quantification of isoflavone in *Momordica dioica* roxb. Ex wild (cucurbitaceae) fruits. Int. J. Appl. Res. Nat. Prod..

[167] Choi S.-J., Shin S.-C., Choi J.-S. (2011). Effects of myricetin on the bioavailability of doxorubicin for oral drug delivery in rats: Possible role of CYP3A4 and P-glycoprotein inhibition by myricetin. Arch. Pharm. Res..

[168] Wesołowska O., Hendrich A.B., Łania-Pietrzak B., Wiśniewski J., Molnar J., Ocsovszki I., Michalak K. (2009). Perturbation of the lipid phase of a membrane is not involved in the modulation of MRP1 transport activity by flavonoids. Cell. Mol. Biol. Lett..

[169] Sabina H., Aliya R. (2009). Seaweed as a new source of flavone, scutellarein 4′-methyl-ether. Pak. J. Bot..

[170] Han Y.-L., Li D., Yang Q.-J., Zhou Z.-Y., Liu L.-Y., Li B., Lu J., Guo C. (2014). In vitro inhibitory effects of scutellarin on six human/rat cytochrome p450 enzymes and P-glycoprotein. Molecules.

[171] He L., Zhao C., Yan M., Zhang L.Y., Xia Y.Z. (2009). Inhibition of P-glycoprotein function by procyanidine on blood–brain barrier. Phytother. Res..

[172] Zhao B.-X., Sun Y.-B., Wang S.-Q., Duan L., Huo Q.-L., Ren F., Li G.-F. (2013). Grape seed procyanidin reversal of P-glycoprotein associated multi-drug resistance via down-regulation of NF-κB and MAPK/ERK mediated YB-1 activity in a2780/t cells. PLoS ONE.

[173] Imai Y., Tsukahara S., Asada S., Sugimoto Y. (2004). Phytoestrogens/flavonoids reverse breast cancer resistance protein/ABCG2-mediated multidrug resistance. Cancer Res..

[174] Lan K., Tian Y., Tan F., Jiang X.H., Wang L. (2008). Intra-herb pharmacokinetics interaction between quercetin and isorhamentin1. Acta Pharmacol. Sin..

[175] Jeong Y.H., Chung S.Y., Han A.R., Sung M.K., Jang D.S., Lee J., Kwon Y., Lee H.J., Seo E.K. (2007). P-glycoprotein inhibitory activity of two phenolic compounds, (−)-syringaresinol and tricin from *Sasa borealis*. Chem. Biodivers..

[176] Enomoto R., Koshiba C., Suzuki C., Lee E. (2011). Wogonin potentiates the antitumor action of etoposide and ameliorates its adverse effects. J. Cancer Chemother. Pharmacol..

[177] Li C., Kim M., Choi H., Choi J. (2011). Effects of baicalein on the pharmacokinetics of tamoxifen and its main metabolite, 4-hydroxytamoxifen, in rats: Possible role of cytochrome P450 3A4 and P-glycoprotein inhibition by baicalein. Arch. Pharm. Res..

[178] Nguyen H., Zhang S., Morris M.E. (2003). Effect of flavonoids on MRP1-mediated transport in panc-1 cells. J. Pharm. Sci..

[179] Tyagi A.K., Singh R.P., Agarwal C., Chan D.C., Agarwal R. (2002). Silibinin strongly synergizes human prostate carcinoma DU145 cells to doxorubicin-induced growth inhibition, G2-M arrest, and apoptosis. Clin. Cancer Res..

[180] Mahringer A., Karamustafa S., Klotz D., Kahl S., Konkimalla V.B., Wang Y., Wang J., Liu H.-Y., Boechzelt H., Hao X. (2010). Inhibition of P-glycoprotein at the blood–brain barrier by phytochemicals derived from traditional chinese medicine. Cancer Genom. Proteom..

[181] Choi J.-S., Choi B.-C., Kang K.W. (2009). Effect of resveratrol on the pharmacokinetics of oral and intravenous nicardipine in rats: Possible role of P-glycoprotein inhibition by resveratrol. Die Pharm. Int. J. Pharm. Sci..

[182] Nabekura T., Kamiyama S., Kitagawa S. (2005). Effects of dietary chemopreventive phytochemicals on P-glycoprotein function. Biochem. Biophys. Res. Commun..

[183] Quan F., Pan C., Ma Q., Zhang S., Yan L. (2008). Reversal effect of resveratrol on multidrug resistance in kbv200 cell line. Biomed. Pharmacother..

[184] Bedada S.K., Yellu N.R., Neerati P. (2016). Effect of resveratrol on the pharmacokinetics of fexofenadine in rats: Involvement of P-glycoprotein inhibition. Pharmacol. Rep..

[185] da Graça Rocha G., Simoes M., Lúcio K.A., Oliveira R.R., Kaplan M.A.C., Gattass C.R. (2007). Natural triterpenoids from *Cecropia lyratiloba* are cytotoxic to both sensitive and multidrug resistant leukemia cell lines. Bioorg. Med. Chem..

[186] Rojas R., Caviedes L., Aponte J.C., Vaisberg A.J., Lewis W.H., Lamas G., Sarasara C., Gilman R.H., Hammond G.B. (2006). Aegicerin, the first oleanane triterpene with wide-ranging antimycobacterial activity, isolated from *Clavija procera*. J. Nat. Prod..

[187] Wortelboer H.M., Usta M., van Zanden J.J., van Bladeren P.J., Rietjens I.M., Cnubben N.H. (2005). Inhibition of multidrug resistance proteins MRP1 and MRP2 by a series of α,β-unsaturated carbonyl compounds. Biochem. Pharmacol..

[188] Corea G., Di Pietro A., Dumontet C., Fattorusso E., Lanzotti V. (2009). Jatrophane diterpenes from euphorbia spp. As modulators of multidrug resistance in cancer therapy. Phytochem. Rev..

[189] Reis M., Ferreira R.J., Santos M.M., Dos Santos D.J., Molnár J., Ferreira M.-J.U. (2013). Enhancing macrocyclic diterpenes as multidrug-resistance reversers: Structure–Activity studies on jolkinol D derivatives. J. Med. Chem..

[190] Duarte N., Varga A., Cherepnev G., Radics R., Molnár J., Ferreira M.-J.U. (2007). Apoptosis induction and modulation of P-glycoprotein mediated multidrug resistance by new macrocyclic lathyrane-type diterpenoids. Bioorg. Med. Chem..

[191] Valente I.S., Reis M., Duarte N.L., Serly J., Molnár J.P., Ferreira M.-J.U. (2012). Jatrophane diterpenes from *Euphorbia mellifera* and their activity as P-glycoprotein modulators on multidrug-resistant mouse lymphoma and human colon adenocarcinoma cells. J. Nat. Prod..

[192] Molnár J., Gyémánt N., Tanaka M., Hohmann J., Bergmann-Leitner E., Molnár P., Deli J., Didiziapetris R., Ferreira M.J. (2006). Inhibition of multidrug resistance of cancer cells by natural diterpenes, triterpenes and carotenoids. Curr. Pharm. Des..

[193] Madureira A.M., Gyémánt N., Ascenso J.R., Abreu P.M., Molnár J., Ferreira M.-J.U. (2006). Euphoportlandols a and b, tetracylic diterpene polyesters from *euphorbia portlandica* and their anti-mdr effects in cancer cells. J. Nat. Prod..

[194] Ferreira M.-J.U., Gyemant N., Madureira A.M., Molnar J. (2005). Inhibition of P-glycoprotein transport activity in a resistant mouse lymphoma cell line by diterpenic lactones. Anticancer Res..

[195] Duarte N., Járdánházy A., Molnár J., Hilgeroth A., Ferreira M.-J.U. (2008). Synergistic interaction between P-glycoprotein modulators and epirubicine on resistant cancer cells. Bioorg. Med. Chem..

[196] Li X., Hu J., Wang B., Sheng L., Liu Z., Yang S., Li Y. (2014). Inhibitory effects of herbal constituents on P-glycoprotein in vitro and in vivo: Herb–drug interactions mediated via P-gp. Toxicol. Appl. Pharmacol..

[197] Fernandes J., Castilho R.O., da Costa M.R., Wagner-Souza K., Kaplan M.A.C., Gattass C.R. (2003). Pentacyclic triterpenes from *Chrysobalanaceae* sp.: Cytotoxicity on multidrug resistant and sensitive leukemia cell lines. Cancer Lett..

[198] Wibowo M., Wang Q., Holst J., White J.M., Hofmann A., Davis R.A. (2016). Dihydro-β-agarofurans from the australian endemic rainforest plant *Denhamia pittosporoides* inhibit leucine transport in prostate cancer cells. Asian J. Org. Chem..

[199] Torres-Romero D., Muñoz-Martínez F., Jiménez I.A., Castanys S., Gamarro F., Bazzocchi I.L. (2009). Novel dihydro-β-agarofuran sesquiterpenes as potent modulators of human P-glycoprotein dependent multidrug resistance. Org. Biomol. Chem..

[200] Cortés-Selva F., Campillo M., Reyes C.P., Jiménez I.A., Castanys S., Bazzocchi I.L., Pardo L., Gamarro F., Ravelo A.G. (2004). Sar studies of dihydro-β-agarofuran sesquiterpenes as inhibitors of the multidrug-resistance phenotype in a *Leishmania tropica* line overexpressing a P-glycoprotein-like transporter. J. Med. Chem..

[201] Han Y.L., Yu H.L., Li D., Meng X.L., Zhou Z.Y., Yu Q., Zhang X.Y., Wang F.J., Guo C. (2011). Inhibitory effects of limonin on six human cytochrome P450 enzymes and P-glycoprotein in vitro. Toxicol. In Vitro.

[202] Smith E., Williamson E., Zloh M., Gibbons S. (2005). Isopimaric acid from pinus nigra shows activity against multidrug-resistant and emrsa strains of *Staphylococcus aureus*. Phytother. Res..

[203] Smith E.C., Kaatz G.W., Seo S.M., Wareham N., Williamson E.M., Gibbons S. (2007). The phenolic diterpene totarol inhibits multidrug efflux pump activity in *Staphylococcus aureus*. Antimicrob. Agents Chemother..

[204] Kashiwada Y., Nishimura K., Kurimoto S., Takaishi Y. (2011). New 29-nor-cycloartanes with a 3,4-seco-and a novel 2,3-seco-structure from the leaves of *Sinocalycanthus chinensis*. Bioorg. Med. Chem..

[205] Jain S., Abraham I., Carvalho P., Kuang Y.-H., Shaala L.A., Youssef D.T., Avery M.A., Chen Z.-S., El Sayed K.A. (2009). Sipholane triterpenoids: Chemistry, reversal of ABCB1/P-glycoprotein-mediated multidrug resistance, and pharmacophore modeling. J. Nat. Prod..

[206] Jain S., Laphookhieo S., Shi Z., Fu L.-W., Akiyama S.-I., Chen Z.-S., Youssef D.T., van Soest R.W., El Sayed K.A. (2007). Reversal of P-glycoprotein-mediated multidrug resistance by sipholane triterpenoids. J. Nat. Prod..

[207] Huang C., Xu D., Xia Q., Wang P., Rong C., Su Y. (2012). Reversal of P-glycoprotein-mediated multidrug resistance of human hepatic cancer cells by astragaloside II. J. Pharm. Pharmacol..

[208] Nguyen V.T.B., Darbour N., Bayet C., Doreau A., Raad I., Phung B.H., Dumontet C., Di Pietro A., Dijoux-Franca M.G., Guilet D. (2009). Selective modulation of P-glycoprotein activity by steroidal saponines from *Paris polyphylla*. Fitoterapia.

[209] Manda V.K., Dale O.R., Awortwe C., Ali Z., Khan I.A., Walker L.A., Khan S.I. (2014). Evaluation of drug interaction potential of *Labisia pumila* (kacip fatimah) and its constituents. Front. Pharmacol..

[210] Hu Y.-J., Shen X.-L., Lu H.-L., Zhang Y.-H., Huang X.-A., Fu L.-C., Fong W.-F. (2008). Tenacigenin B derivatives reverse P-glycoprotein-mediated multidrug resistance in HepG2/dox cells. J. Nat. Prod..

[211] Ramalhete C., Spengler G., Martins M., Viveiros M., Mulhovo S., Ferrira M.J.V., Amaral L. (2011). Inhibition of efflux pumps in Methicillin-resistant *Staphylococcus aureus* and *Enterococcus faecalis* resistant strains by triterpenoids from *Momordica balsamina*. Int. J. Antimicrob. Agents.

[212] Ramalhete C., Molnár J., Mulhovo S., Rosário V.E., Ferreira M.-J.U. (2009). New potent P-glycoprotein modulators with the cucurbitane scaffold and their synergistic interaction with doxorubicin on resistant cancer cells. Bioorg. Med. Chem..

[213] Choi C.-H., Kang G., Min Y.-D. (2003). Reversal of P-glycoprotein-mediated multidrug resistance by protopanaxatriol ginsenosides from Korean red ginseng. Planta Med..

[214] Risinger A.L., Jackson E.M., Polin L.A., Helms G.L., LeBoeuf D.A., Joe P.A., Hopper-Borge E., Ludueña R.F., Kruh G.D., Mooberry S.L. (2008). The taccalonolides: Microtubule stabilizers that circumvent clinically relevant taxane resistance mechanisms. Cancer Res..

[215] Aoki S., Yoshioka Y., Miyamoto Y., Higuchi K., Setiawan A., Murakami N., Chen Z.-S., Sumizawa T., Akiyama S.-I., Kobayashi M. (1998). Agosterol A, a novel polyhydroxylated sterol acetate reversing multidrug resistance from a marine sponge of *Spongia* sp.. Tetrahedron Lett..

[216] Rubis B., Polrolniczak A., Knula H., Potapinska O., Kaczmarek M., Rybczynska M. (2010). Phytosterols in physiological concentrations target multidrug resistant cancer cells. Med. Chem..

[217] Song J.S., Chae J.-W., Lee K.-R., Lee B.H., Choi E.J., Ahn S.H., Kwon K.-I., Bae M.A. (2011). Pharmacokinetic characterization of decursinol derived from *Angelica gigas* Nakai in rats. Xenobiotica.

[218] Kimura S., Ito C., Jyoko N., Segawa H., Kuroda J., Okada M., Adachi S., Nakahata T., Yuasa T., Furukawa H. (2005). Inhibition of leukemic cell growth by a novel anti-cancer drug (GUT-70) from *Calophyllum brasiliense* that acts by induction of apoptosis. Int. J. Cancer.

[219] Ohnishi A., Matsuo H., Yamada S., Takanaga H., Morimoto S., Shoyama Y., Ohtani H., Sawada Y. (2000). Effect of furanocoumarin derivatives in grapefruit juice on the uptake of vinblastine by caco-2 cells and on the activity of cytochrome p450 3a4. Br. J. Pharmacol..

[220] Deferme S., Augustijns P. (2003). The effect of food components on the absorption of P-gp substrates: A review. J. Pharm. Pharmacol..

[221] Kasaian J., Mosaffa F., Behravan J., Masullo M., Piacente S., Ghandadi M., Iranshahi M. (2015). Reversal of P-glycoprotein-mediated multidrug resistance in MCF-7/ADR cancer cells by sesquiterpene coumarins. Fitoterapia.

[222] Hanafi-Bojd M.Y., Iranshahi M., Mosaffa F., Tehrani S.O., Kalalinia F., Behravan J. (2011). Farnesiferol A from *Ferula persica* and galbanic acid from *Ferula szowitsiana* inhibit P-glycoprotein-mediated rhodamine efflux in breast cancer cell lines. Planta Med..

[223] Barthomeuf C., Demeule M., Grassi J., Saidkhodjaev A., Beliveau R. (2006). Conferone from *Ferula schtschurowskiana* enhances vinblastine cytotoxicity in MDCK-MDR1 cells by competitively inhibiting P-glycoprotein transport. Planta Med..

[224] Bazzaz B.S.F., Memariani Z., Khashiarmanesh Z., Iranshahi M., Naderinasab M. (2010). Effect of galbanic acid, a sesquiterpene coumarin from *Ferula szowitsiana*, as an inhibitor of efflux mechanism in resistant clinical isolates of *staphylococcus aureus*. Braz. J. Microbiol..

[225] Sarkhail P., Shafiee A., Sarkheil P. (2013). Biological activities and pharmacokinetics of praeruptorins from Peucedanum species: A systematic review. BioMed. Res. Int..

[226] Barthomeuf C., Grassi J.M., Demeule M., Fournier C., Boivin D., Beliveau R. (2005). Inhibition of P-glycoprotein transport function and reversion of MDR1 multidrug resistance by cnidiadin. Cancer Chemother. Pharmacol..

[227] Kowalski R.J., Giannakakou P., Gunasekera S.P., Longley R.E., Day B.W., Hamel E. (1997). The microtubule-stabilizing agent discodermolide competitively inhibits the binding of paclitaxel (taxol) to tubulin polymers, enhances tubulin nucleation reactions more potently than paclitaxel, and inhibits the growth of paclitaxel-resistant cells. Mol. Pharmacol..

[228] Aoki S., Cao L., Matsui K., Rachmat R., Akiyama S.I., Kobayashi M. (2004). Kendarimide A, a novel peptide reversing P-glycoprotein-mediated multidrug resistance in tumor cells, from a marine sponge of *Haliclona* sp.. Tetrahedron.

[229] Stratmann K., Burgoyne D.L., Moore R.E., Patterson G.M., Smith C.D. (1994). Hapalosin, a cyanobacterial cyclic depsipeptide with multidrug-resistance reversing activity. J. Org. Chem..

[230] Raju R., Piggott A.M., Huang X.-C., Capon R.J. (2011). Nocardioazines: A novel bridged diketopiperazine scaffold from a marine-derived bacterium inhibits P-glycoprotein. Org. Lett..

[231] Wang X., Deng R., Lu Y., Xu Q., Yan M., Ye D., Chen W. (2013). Gambogic acid as a non-competitive inhibitor of ATP-binding cassette transporter B1 reverses the multidrug resistance of human epithelial cancers by promoting ATP-binding cassette transporter B1 protein degradation. Basic Clin. Pharmacol. Toxicol..

[232] Pereda-Miranda R., Kaatz G.W., Gibbons S. (2006). Polyacylated oligosaccharides from medicinal Mexican morning glory species as antibacterials and inhibitors of multidrug resistance in *Staphylococcus aureus*. J. Nat. Prod..

[233] Kourtesi C., Ball A.R., Huang Y.-Y., Jachak S.M., Vera D.M.A., Khondkar P., Gibbons S., Hamblin M.R., Tegos G.P. (2013). Microbial efflux systems and inhibitors: Approaches to drug discovery and the challenge of clinical implementation. Open Microbiol. J..

[234] Su J., Lai H., Chen J., Li L., Wong Y.-S., Chen T., Li X. (2013). Natural borneol, a monoterpenoid compound, potentiates selenocystine-induced apoptosis in human hepatocellular carcinoma cells by enhancement of cellular uptake and activation of ROS-mediated DNA damage. PLoS ONE.

[235] Soenen D.R., Hwang I., Hedrick M.P., Boger D.L. (2003). Multidrug resistance reversal activity of key ningalin analogues. Bioorg. Med. Chem. Lett..

[236] Musumeci R., Speciale A., Costanzo R., Annino A., Ragusa S., Rapisarda A., Pappalardo M., Iauk L. (2003). Berberis aetnensis C. Presl. extracts: Antimicrobial properties and interaction with ciprofloxacin. Int. J. Antimicrob. Agents.

[237] Spitaler M., Utz I., Hilbe W., Hofmann J., Grunicke H. (1998). Pkc-independent modulation of multidrug resistance in cells with mutant (v185) but not wild-type (g185) P-glycoprotein by bryostatin 1. Biochem. Pharmacol..

[238] Zhu H.-J., Wang J.-S., Markowitz J.S., Donovan J.L., Gibson B.B., Gefroh H.A., DeVane C.L. (2006). Characterization of P-glycoprotein inhibition by major cannabinoids from marijuana. J. Pharmacol. Exp. Ther..

[239] Feinshtein V., Erez O., Ben-Zvi1 Z., Erez N., Eshkoli T., Sheizaf B., Sheiner E., Huleihel M., Holcberg G. (2013). Cannabidiol changes P-gp and BCRP expression in trophoblast cell lines. Peer J..

[240] Fan X., Chai L., Zhang H., Wang Y., Zhang B., Gao X. (2015). Borneol Depresses P-Glycoprotein Function by a NF-κB Signaling Mediated Mechanism in a Blood Brain Barrier In Vitro Model. Int. J. Mol. Sci..

[241] Saab A.M., Guerrini A., Sacchetti G., Maietti S., Zeino M., Arend J., Gambari R., Bernardi F., Efferth T. (2012). Phytochemical analysis and cytotoxicity towards multidrug-resistant leukemia cells of essential oils derived from lebanese medicinal plants. Planta Med..

[242] Stermitz F.R., Cashman K.K., Halligan K.M., Morel C., Tegos G.P., Lewis K. (2003). Polyacylated neohesperidosides from *Geranium caespitosum*: Bacterial multidrug resistance pump inhibitors. Bioorg. Med. Chem. Lett..

[243] Hiramatsu K., Cui L., Kuroda M., Ito T. (2001). The emergence and evolution of methicillin-resistant *Staphylococcus aureus*. Trends Microbiol..

[244] Zhang X., Ao Z., Bello A., Ran X., Liu S., Wigle J., Kobinger G., Yao X. (2016). Characterization of the inhibitory effect of an extract of *Prunella vulgaris* on ebola virus glycoprotein (gp)-mediated virus entry and infection. Antivir. Res..

[245] Wan C.-K., Zhu G.-Y., Shen X.-L., Chattopadhyay A., Dey S., Fong W.-F. (2006). Gomisin A alters substrate interaction and reverses P-glycoprotein-mediated multidrug resistance in HepG2-DR cells. Biochem. Pharmacol..

[246] Chung S.Y., Han A.R., Sung M.K., Jung H.J., Nam J.W., Seo E.K., Lee H.J. (2009). Potent modulation of P-glycoprotein activity by naturally occurring phenylbutenoids from *Zingiber cassumunar*. Phytother. Res..

[247] Dewick P.M. (2002). Medicinal Natural Products: A Biosynthetic Approach.

[248] Cordon-Cardo C., O’Brien J.P., Casals D., Rittman-Grauer L., Biedler J.L., Melamed M.R., Bertino J.R. (1989). Multidrug-resistance gene (P-glycoprotein) is expressed by endothelial cells at blood-brain barrier sites. Proc. Natl. Acad. Sci. USA.

[249] Wilson C.M., Volkman S.K., Thaithong S., Martin R.K., Kyle D.E., Milhous W.K., Wirth D.F. (1993). Amplification of pfmdr1 associated with mefloquine and halofantrine resistance in *Plasmodium falciparum* from thailand. Mol. Biochem. Parasitol..

[250] Descoteaux S., Ayala P., Samuelson J., Orozco E. (1995). Increase in mRNA of multiple EH P-gp genes encoding P-glycoprotein homologues in emetine-resistant *Entamoeba histolytica* parasites. Gene.

[251] Gamarro F., Chiquero M.J., Amador M.V., Légaré D., Ouellette M., Castanys S. (1994). P-glycoprotein overexpression in methotrexate-resistant *Leishmania tropica*. Biochem. Pharmacol..

[252] Munagala S., Sirasani G., Kokkonda P., Phadke M., Krynetskaia N., Lu P., Sharom F.J., Chaudhury S., Abdulhameed M.D.M., Tawa G. (2014). Synthesis and evaluation of strychnos alkaloids as MDR reversal agents for cancer cell eradication. Bioorg. Med. Chem..

[253] Innocenti F., Ratain M.J. (2003). Irinotecan treatment in cancer patients with ugt1a1 polymorphisms. Oncology.

[254] Okyar A., Piccolo E., Ahowesso C., Filipski E., Hossard V., Guettier C., La Sorda R., Tinari N., Iacobelli S., Lévi F. (2011). Strain-and sex-dependent circadian changes in ABCC2 transporter expression: Implications for irinotecan chronotolerance in mouse ileum. PLoS ONE.

[255] Johnson B.M., Charman W.N., Porter C.J. (2003). Application of compartmental modeling to an examination of in vitro intestinal permeability data: Assessing the impact of tissue uptake, P-glycoprotein, and CYP3A. Drug Metab. Dispos..

[256] Kemper E.M., van Zandbergen A.E., Cleypool C., Mos H.A., Boogerd W., Beijnen J.H., van Tellingen O. (2003). Increased penetration of paclitaxel into the brain by inhibition of P-glycoprotein. Clin. Cancer Res..

[257] Filipski E., Berland E., Ozturk N., Guettier C., van der Horst G.T., Lévi F., Okyar A. (2014). Optimization of irinotecan chronotherapy with P-glycoprotein inhibition. Toxicol. Appl. Pharmacol..

[258] Seral C., Michot J.-M., Chanteux H., Mingeot-Leclercq M.-P., Tulkens P.M., Van Bambeke F. (2003). Influence of P-glycoprotein inhibitors on accumulation of macrolides in J774 murine macrophages. Antimicrob. Agents Chemother..

[259] Yu J., Zhou P., Asenso J., Yang X.-D., Wang C., Wei W. (2016). Advances in plant-based inhibitors of Pglycoprotein. J. Enzyme Inhib. Med. Chem..

[260] Phang J.M., Poore C.M., Lopaczynska J., Yeh G.C. (1993). Flavonol stimulated efflux of 7,12-dimethylbenz(*a*)-anthracene in multidrug-resistant breast cancer cells. Cancer Res..

[261] Critchfield J.W., Welsh C.J., Phang J.M., Yeh G.C. (1994). Modulation of Adriamycin accumulation and efflux by flavonoids in HCT-15 colon cells: Activation of Pglycoprotein as a putative mechanism. Biochem. Pharmacol..

[262] Wink M., Ashour M.L., El-Readi M.Z. (2012). Secondary metabolites from plants inhibiting ABC transporters and reversing resistance of cancer cells and microbes to cytotoxic and antimicrobial agents. Front. Microbiol..

